# Histone deacetylase inhibition leads to regulatory histone mark alterations and impairs meiosis in oocytes

**DOI:** 10.1186/s13072-021-00413-8

**Published:** 2021-08-12

**Authors:** Louis Legoff, Ouzna Dali, Elena De La Mata Santaella, Christian Jaulin, Shereen Cynthia D’Cruz, Fatima Smagulova

**Affiliations:** Univ. Rennes, EHESP, Inserm, Irset (Institut de Recherche en Santé, environnement et travail) - UMR_S 1085, 35000 Rennes, France

**Keywords:** Panobinostat, HDAC, Epigenetics, RNA-seq, Ovary, Histone modifications

## Abstract

**Background:**

Panobinostat (PB), a histone deacetylase (HDAC) inhibitor drug, is clinically used in the treatment of cancers. We investigated the effects of PB on murine ovarian functions in embryos and adult animals.

**Methods:**

C57BL/6J mice were treated with 5 mg/kg PB on alternate days from embryonic day (E) 6.5 to E15.5. We analysed the effects of PB on the ovaries by using immunofluorescence, gene expression analysis and DNA methylation analysis techniques.

**Results:**

At E15.5, we observed increases in histone H3K9Ac, H4Ac and H3K4me3 marks, while the level of the silencing H3K9me3 mark decreased. Synaptonemal complex examination at E15.5, E17.5 and E18.5 showed a delay in meiotic progression characterized by the absence of synaptonemal complexes at E15.5 and the persistence of double-strand breaks (DSBs) at E17.5 and E18.5 in PB-exposed oocytes. We found that exposure to PB led to changes in the expression of 1169 transcripts at E15.5. Genes regulated by the male-specific factors SRY-Box Transcription Factor 9 (SOX9) and Doublesex and Mab-3-related Transcription factor 1 (DMRT1) were among the most upregulated genes in the ovaries of PB-exposed mice. In contrast, PB treatment led to decreases in the expression of genes regulated by the WNT4 pathway. Notably, we observed 119 deregulated genes encoding Zn-finger proteins. The observed alterations in epigenetic marks and gene expression correlated with decreases in the numbers of germ cells at E15.5. After birth, PB-exposed ovaries showed increased proliferation of primary and secondary follicles. We also observed decreases in the numbers of primordial, primary and secondary follicles in adult ovaries from mice that were exposed to PB in utero. Finally, epigenetic alterations such as decreased H3K4me3 and increased H4 acetylation levels were also detected in somatic cells surrounding fully grown oocytes.

**Conclusion:**

Our data suggest that inhibition of histone deacetylase by PB during a critical developmental window affects reprogramming and germ cell specification via alteration of epigenetic marks.

**Supplementary Information:**

The online version contains supplementary material available at 10.1186/s13072-021-00413-8.

## Introduction

Germ cells are formed during development by a process known as somatic-to-germline transition (SGT). During SGT, global epigenetic reprogramming occurs wherein DNA methylation and histone post-translational marks are reset to create a new lineage of germ cells. The coordinated actions of transcription, chromatin remodelling and histone-modifying factors are critical for the establishment of the germ cell population lineage [1, 2]. During this period, germ cells lose their DNA methylation marks. The demethylation of *Ddx4*, *Dazl* and *Hormad1* is critical for germ cell development [3]. In addition, normal expression of the genes encoding somatic factors *Rspo1, Wnt4* and *Foxl2* is required for the development of female gonads [4]. In oogonia, the production of retinoic acid (RA) by mesonephric cells induces *Stra8* expression that in turn triggers oogonia towards meiosis onset [5]. A critical event that takes place during meiosis is homologous recombination (HR)-mediated exchange of genetic information between parental chromosomes. HR is initiated with the introduction of DSBs by the topoisomerase-like enzyme SPO11 [6] at discrete places called recombination hotspots scattered throughout the genome [7]. Next, the recombinase proteins DMC1 and RAD51 initiate homologous chromosome searching, and the maternal and paternal chromosomes become paired. All DSBs are eventually repaired, and the homologous chromosomes remain connected at crossover locations [8]. At the end of prophase I, oocytes are arrested at the diplotene stage [9].

Epigenetic mechanisms control all the steps of germ cell development, from lineage establishment and meiotic progression [10, 11] to oocyte growth [12] and maturation [13, 14]. For example, during meiosis, histone H3 trimethylation at lysine 4 and lysine 36 [15] and histone H3K9Ac [16] marks are highly enriched at recombination hotspots. Pericentromeric epigenetic marks (H3K9me2 and H3K9me3) are also important for proper chromosome orientation during synapse formation and maintenance [17]. The role of histone acetylation has been studied in many somatic cell models. For example, it has been shown that histone acetylation plays an important role in DNA repair [18, 19] and chromatin remodelling [20]. H4 acetylation at lysine 16 (H4K16) is also detected at DSB sites [21, 22]. In response to DNA damage, male-absent-on-the-firs (MOF) directly interacts with ATM and activates it [23]. Cells mutated for TIP60 (KAT5), which has histone H4 acetyltransferase activity, show defects in DSB repair [24]. However, another histone mark, H3K56Ac, plays an important role in the DNA damage response [25]. Histone acetyltransferases (HATs) catalyse acetylation reactions, whereas acetyl groups are removed by histone deacetylases (HDACs). The activities of HATs and HDACs are tightly controlled through targeted recruitment, protein–protein interactions, and post-translational modifications, as reviewed in [26].

It is suggested that HDACs are also important for germ cell development. Mice haplodeficient for *Hdac1* and harbouring *Hdac2-*deficient oocytes are infertile. It has been proposed that histone deacetylation that occurs during oocyte maturation is critical for proper chromosome segregation [27]. Exposure to the HDAC inhibitor trichostatin A (TSA) leads to histone hyperacetylation, which correlates with chromosome misalignment. TSA treatment causes abnormal axial chromatid condensation that results in the formation of elongated chromosomes, and these defects are associated with hyperacetylation of histone H4 at lysines 5 and 16 [28].

In this study, we investigated the consequences of exposure to the powerful HDAC inhibitor panobinostat (PB) on oocyte meiosis. We hypothesized that inhibition of histone deacetylases during a critical reprogramming window could alter the epigenetic status of germ cell-specific gene networks and affect gonad development and oogenesis.

We report that in utero PB exposure leads to alterations in several histone modifications, deregulates sex-specific gene expression and decreases germ cell numbers in the embryonic ovaries. In adult murine ovaries, we observed alterations in histone marks, gene expression and follicle numbers.

## Results

### Experimental design

This study aimed to reveal the effects of histone acetylation perturbation on female germ cell development with a focus on embryonic events in which meiosis, the chromosome segregation process allowing the formation of haploid gametes, is initiated. Inbred C57BL/6J mice were treated with PB during the somatic-to-germline transition window, which corresponds to embryonic day (E) 6.5 to E15.5. Mice were dissected at several time points: E15.5, E17.5, E18.5, post-natal day (PND) 5 and 5 months. A schematic presentation of the experiments is presented in Additional file 1: Fig. S1. To analyse the effects of PB on female germ cells, we treated mice with a dose of PB equal to 5 mg/kg every other day. We observed a significant 13% increase in the body weight of the embryos at E15.5 but not at E17.5 (Additional file 1: Fig. S2). We did not observe significant changes in body or ovary weights in 5-day-old and adult mice, suggesting that the dose used was not grossly toxic (Additional file 1: Figs. S2, S3). No alterations in gestation parameters, such as litter size and gestation time, were observed in PB-treated animals compared to controls (Additional file 1: Fig. S4).

### Increases in H3K9Ac, H4Ac, and H3K4me3 and a decrease in H3K9me3 after PB exposure

**Fig. 1:**
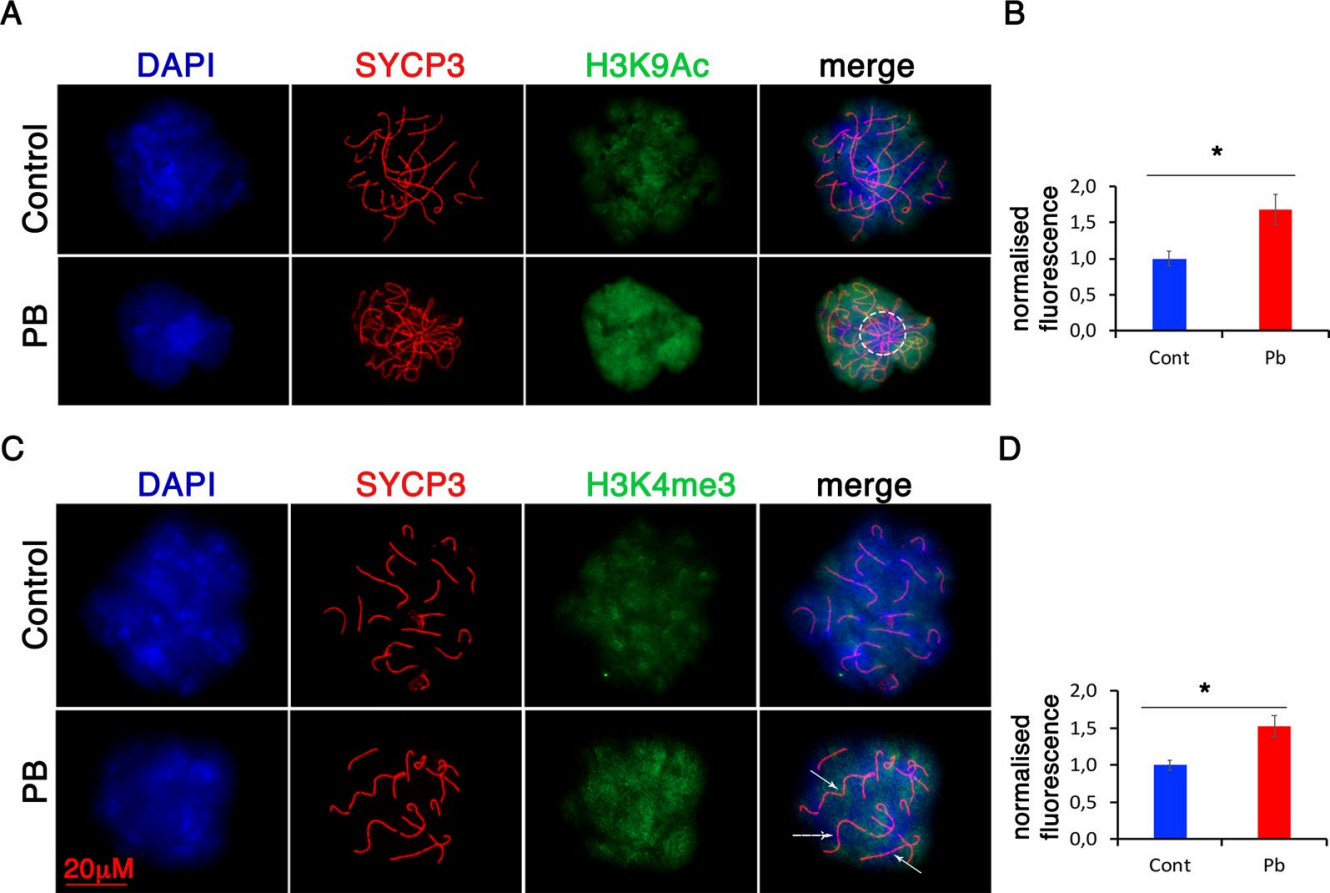
Meiosis defects are associated with increases in histone acetylation and H3K4me3. **A** Surface spreads from E18.5 ovaries from control (first row) and Pb-exposed mice (second row) were immunostained with anti-H3K9Ac (green) and anti-SYCP3 (red) antibodies (63X magnification). The dotted circles represent telomere connections. **B** Quantitative analysis of immunofluorescence intensity. The data are presented as the averaged fluorescence compared to the control ± SEM; *n* = 4 control, *n* = 4 PB group; *p < 0.05, nonparametric Mann–Whitney test. **C** Surface spreads from E185 ovaries from control (first row) and PB-exposed mice (second row) were immunostained with anti-H3K4me3 (green) and anti-SYCP3 (red) antibodies; the arrows represent telomere end-to-end connections. **D** Quantitative analysis of anti-H3K4me3 intensities. The analysis was performed on at least 20 oocytes from four replicates for each group; *n* = 4 for each group; *p < 0.05, nonparametric Mann–Whitney test

To reveal whether gestational exposure to PB affects histone modifications, we analysed the histone marks important for meiosis, H4Ac, H3K9Ac, H3K4me3 and H3K9me3. Defects in histone acetylation regulation may have an impact on histone methylation since methylation and acetylation are mutually exclusive on the lysine epsilon amino group. Therefore, histone acetylation and methylation on lysines must be coordinated, with methylation being able to take place only on deacetylated lysines, and vice versa. Since analyses of histone acetylation levels can provide clues on the efficiency of PB exposure, we immunostained ovarian surface spreads for H3K9Ac and costained the slides with SYCP3 to visualize the synaptonemal complex (SC). H3K9Ac normally appears as strong staining around all chromosomes except at compact heterochromatin (Fig. 1A). Quantitative analysis of H3K9Ac staining showed that in PB-treated oocytes, the intensity of H3K9Ac was increased 1.7-fold (Fig. 1B).

H4 acetylation has been reported to be important for both DNA repair and chromatin remodelling events [29]. Structurally preserved nuclei were immunostained with an anti-H4Ac antibody (Penta, acetylated at residues K5, K8, K12, and K16) (Additional file 1: Fig. S5A) in E15.5 ovaries. Quantitative analysis showed a 1.32-fold increase in H4Ac intensity in PB-exposed oocytes at E15.5 (Additional file 1: Fig. S5B).

We also examined H3K4me3, which is essential for HR, as it is required for the initiation of double-strand breaks. Meiotic spreads were immunostained for H3K4me3 and SYCP3 (Fig. 1C). Quantitative analysis showed that H3K4me3 intensity was increased 1.5-fold in embryonic oocytes from treated mice (Fig. 1D).

H3K9me3 plays a major role in the formation of heterochromatin where telomeres attach during meiosis. Quantification of this histone modification revealed a 1.2-fold decrease in the levels of H3K9me3 in embryonic ovaries (*p* = 0.051) (Additional file 1: Fig. S6A, B).

Thus, our data suggested that epigenetic marks associated with meiosis and DNA repair were altered upon PB exposure in embryonic oocytes.

### The histone demethylase KDM5A is decreased in PB-exposed ovaries

**Fig. 2:**
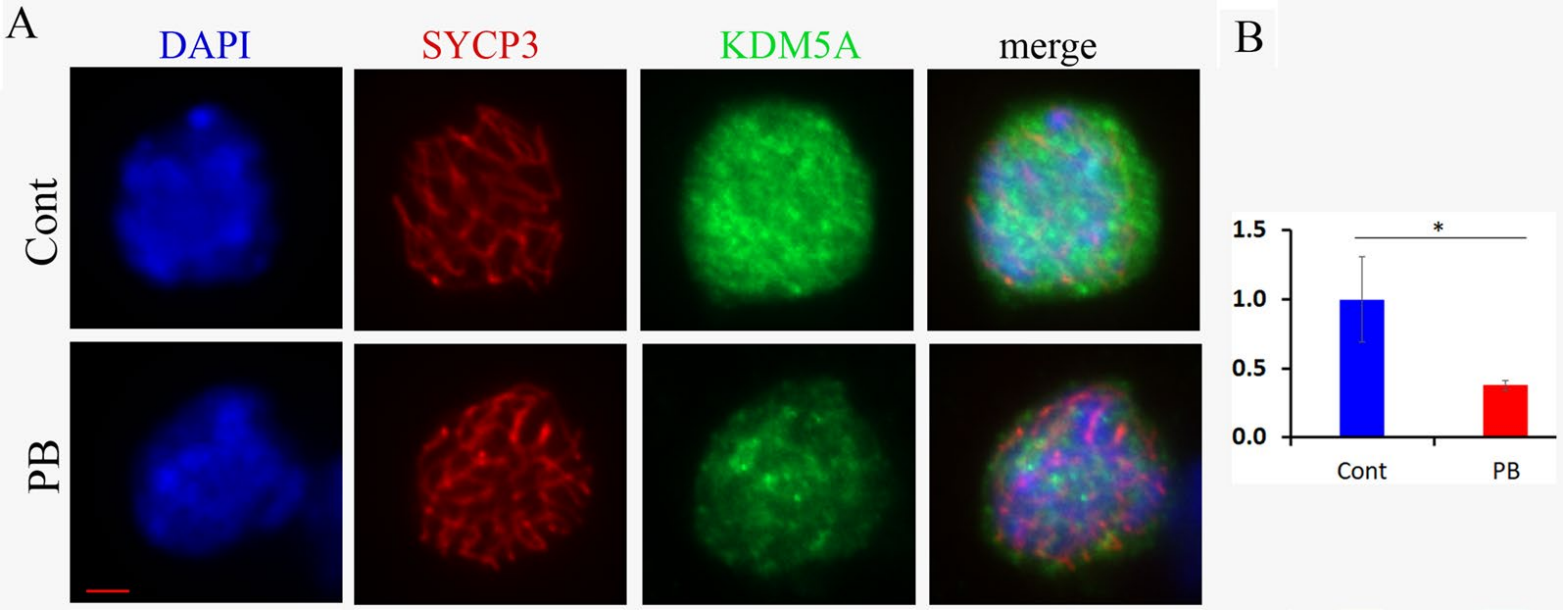
KDM5A is decreased in exposed oocytes. Structurally preserved nuclei from E18,5 ovaries from the control (top row) and PB-exposed (bottom row) groups were immunostained for KDM5A and SYCP3. The combined total corrected fluorescence for KDM5A was calculated for each cell and normalized to the DAPI signal. The average normalized CTCF values are presented in MS-Excel plot ± SD; *p < 0.05, nonparametric Mann–Whitney test. The bar represents 2 μm

Since it has been shown that the demethylase KDM5A is functionally linked to two histone deacetylase complexes [30], we investigated whether it could play a role in PB-exposed ovaries. We immunostained embryonic oocyte cells with antibodies against KDM5A and SYCP3 (Fig. 2A) and performed a quantitative analysis of the z-stack images. Our analysis revealed that KDM5A had strong nuclear staining in the control group but 2.7-fold lower intensity in the PB-exposed groups (Fig. 2B).

### Meiotic defects are increased in PB-exposed ovaries

**Fig. 3:**
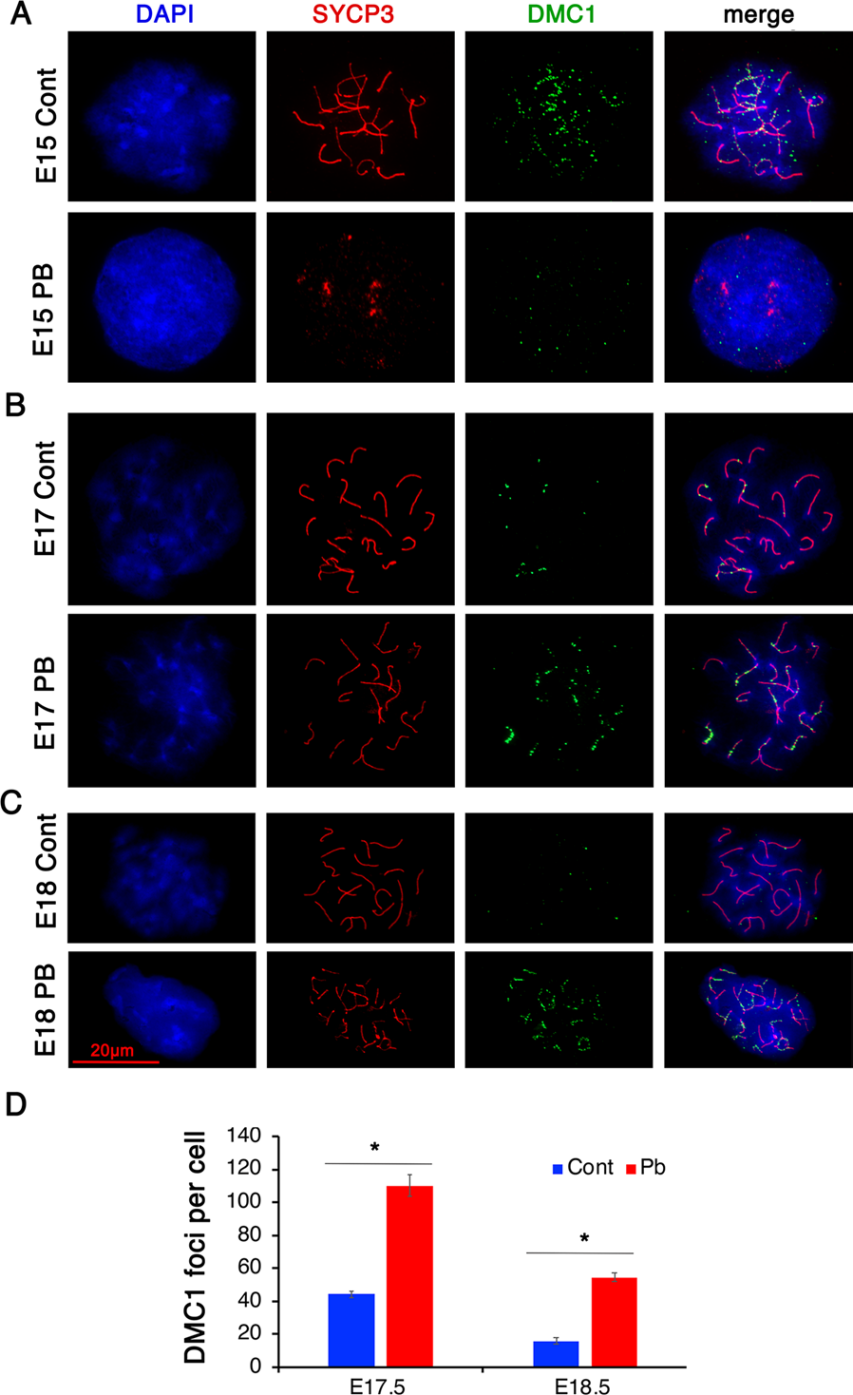
A critical meiotic step is perturbed in embryonic ovaries exposed to PB. Surface spreads from E15.5 control (**A**, top row) and PB-treated ovaries (**A**, bottom row) were immunostained with anti-DMC1 (green) and anti-SYCP3 (red) antibodies (63X magnification). **B** Surface spreads from E17.5 control (**B**, top row) and PB-treated ovaries (**B**, bottom row). **C** Surface spreads from E18.5 control (**C**, top row) and PB-treated ovaries (**C**, bottom row). **D** Quantitative analysis of the number of DMC1 foci per cell performed on at least twenty E17.5 and E18.5 oocytes from four replicates from different litters; *n* = 4 for each group; *p < 0.05, nonparametric Mann–Whitney test

Since we observed alterations in major epigenetic marks involved in DNA repair at recombination hotspots and during meiosis, we investigated whether these epigenetic alterations could influence meiotic DSB repair efficiency. To this end, we immunostained E15.5 surface spreads for the DNA-binding recombination protein DMC1. SYCP3 was also labelled to visualize paired chromosomes (Fig. 3). DSB formation and repair are dynamic processes; new DSBs are formed and repaired simultaneously, so the number of DMC1-stained foci varies from cell to cell. We were not able to observe DSBs at embryonic day 15.5 in PB-exposed cells since the synaptonemal complex (SC) was not fully formed in exposed oocytes and the lateral component of SC, SYCP3, appeared as dots in a few cells (Fig. 3A). At E17.5 and E18.5, DMC1 foci were visible in PB-exposed oocytes (Fig. 3B, C). To evaluate the efficiency of meiotic DNA repair, we measured the average number of DMC1 foci per oocyte in the control and treated groups. We found that the number of DSB foci at E17.5 in the treated group (110 ± 6) was higher than that in the control group (44 ± 2, *p* < 0.05) (Fig. 3D). On embryonic day E18.5, most of the breaks were repaired in untreated control mice (16 ± 2, DMC1 foci per cell); however, in the PB-treated group, the number of DMC1 foci remained high (54 ± 3, *p* < 0.05) (Fig. 3D).

Thus, exposure to PB leads to a delay in the formation of the synaptonemal complex and the persistence of DSBs in embryonic ovaries.

### Exposure to PB leads to gene expression alterations in embryonic ovaries

Next, to determine whether in utero PB exposure affects gene expression, we performed transcriptomics analysis by using strand-specific RNA sequencing. We identified 1169 transcripts whose expression was changed in comparison to the expression in control ovaries (FC ≥ 2, FDR ≤ 0.05). These transcripts represented 2.45% of the total transcriptome (47,623 transcripts expressed in all three biological replicates in the control group) (Additional file 1: Table S1). Of the differentially expressed genes (DEGs), the numbers of upregulated and downregulated genes were nearly equal (Additional file 1: Fig. S7).

**Fig. 4:**
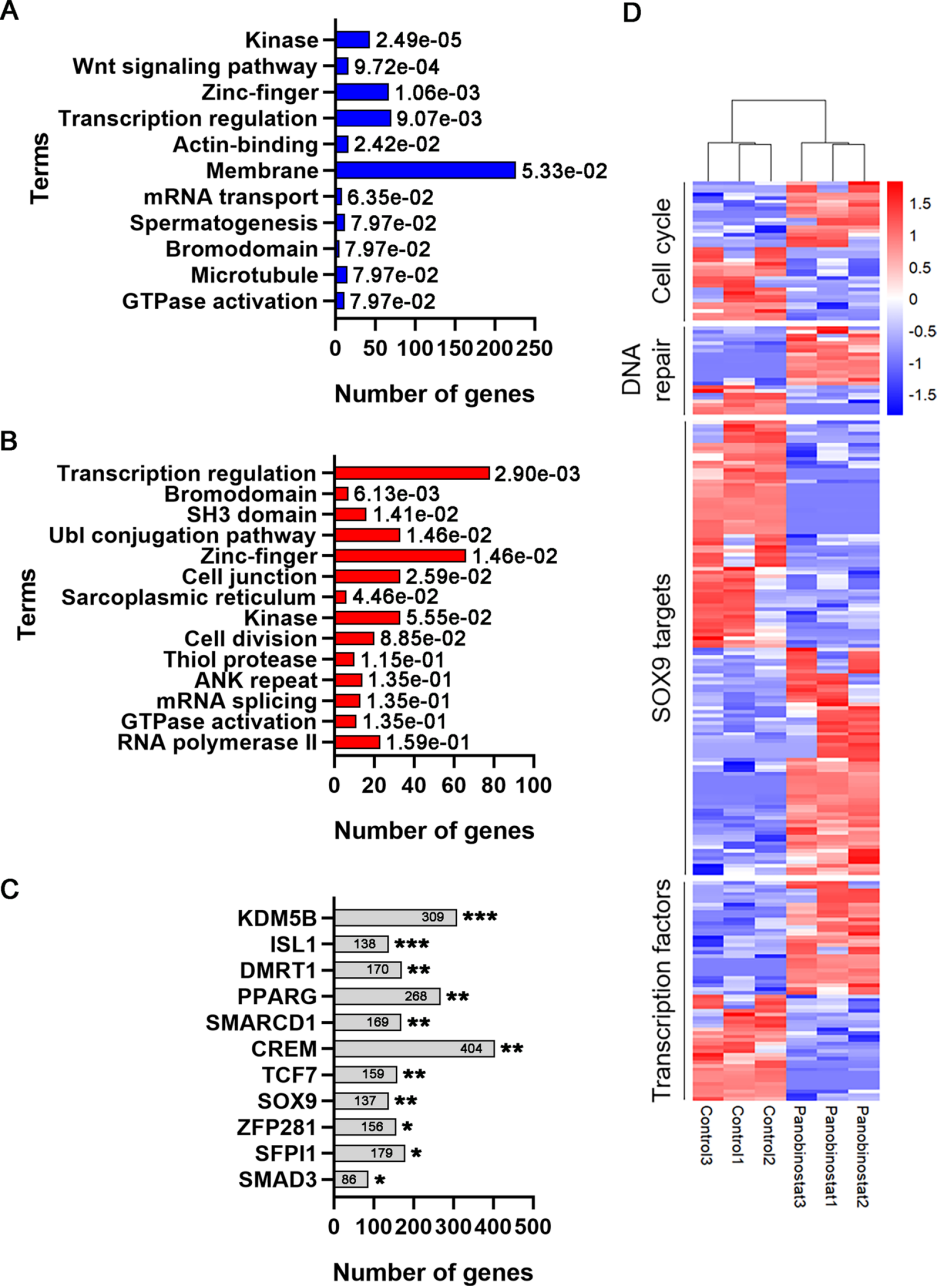
Gestational exposure to PB alters developmental genes, transcription factors and genes encoding Zn-finger proteins. Gene expression analysis was performed by RNA transcriptomic analysis using 3 biological replicates from control and PB-exposed E15.5 ovaries. Gene Ontology function analysis of the downregulated (**A**) and upregulated genes **B** was performed. **C** Analysis using the CHEA2016 dataset from Enrichr showed that many DEGs are regulated by gonad differentiation-specific genes (DMRT1, SOX9). **D** Heatmap illustrating the subset of DEGs encoding the cell cycle, DNA repair, and transcription factors

To reveal the effects of PB treatment on molecular pathways, we performed functional annotation of differentially expressed genes (DEGs) using the DAVID tool. Several functions were overrepresented for the downregulated genes. Notably, we identified enrichment for 16 genes encoding WNT signalling pathway proteins (e.g., *Cxx4*, *Dixdc1*, *Sox17*, *Axin2*, *Hic1*, *Lgr4*, *Rnf146*, *Wnt2*) and spermatogenesis-related proteins (12 genes, e.g., *Sohlh1, Tdrd9, Zfx, Rnf114, Nek1*) (Fig. 4A). For the upregulated genes, we found several functions/characteristics that were overrepresented, including transcriptional regulation (70 genes), Zn-fingers (67 genes) and kinases (43 genes) (Fig. 4B). Thus, our analysis of DEGs showed that genes encoding Zn-finger proteins were among the most altered genes, suggesting that PB exposure mainly affects the expression of these genes.

We also performed an alternative functional annotation using the *ChEA2016* database from *Enrichr*, and we detected upregulated genes that are known to be regulated by male-specific factors. According to the *ChEA2016* database, 137 out of 1384 differentially expressed genes are regulated by SOX9, and 170 out of 2072 are regulated by DMRT1 (Fig. 4C).

Next, we also analysed DEGs using g:Profiler, a functional annotation tool. This analysis showed that a large group of genes were annotated to organism development processes. These genes fell into the cell cycle, DNA repair, and transcription factor categories (Fig. 4D).

Our RNA-seq data did not reveal any alterations in HDAC mRNA expression, except for that of *Hdac7* (transcript variant X6, XM_006521208.3), which was expressed only in PB-exposed mice (FC = 875.93, FDR = 0.056), suggesting that the phenotypes observed following PB exposure were a consequence of direct HDAC inhibition rather than impaired transcriptional regulation of HDAC genes.

To determine whether the PB-induced defects observed in embryonic ovaries were restored after birth, we performed RT-qPCR of some target genes at PND5. We found that genes encoding histone-modifying enzymes, such as *Fgfr1*, *Foxo3*, *Kat2a* and *Kat2b,* were downregulated (Wilcoxon–Mann–Whitney, *p*-value = 0.057) (Additional file 1: Fig. S8), whereas genes encoding DNA repair or apoptosis proteins were not altered (except for the repair protein *Mus81*, which was downregulated). In summary, in utero PB exposure results in mostly transitory transcriptional deregulation of several key functional compartments in the embryonic ovaries.

### DNA methylation tends to be increased at the promoters of the master regulator genes *Dazl, Ddx4* and *Hormad1*

**Fig. 5:**
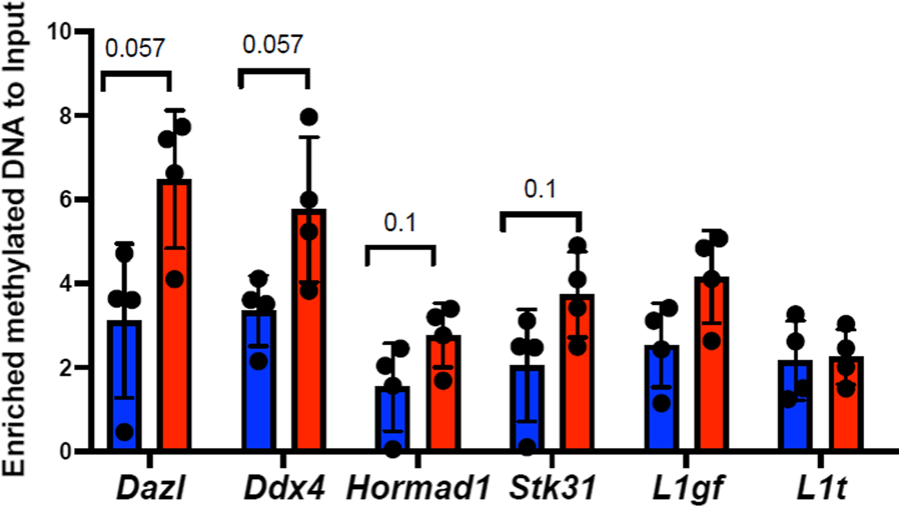
DNA methylation is increased in the promoters of germline master regulator genes. The average values for the methylated DNA-to-input ratios were calculated and plotted. The DNA methylation levels of promoter LINE-1 (L1) retroelements were used as positive controls. A nonparametric Mann–Whitney test was used to assess statistical significance, and the exact p-values are indicated at the tops of the columns

Alterations in gene expression could be a consequence of the effects of PB on the somatic-to-germline transition process, during which somatic cells lose methylation and establish germline lineage-specific marks. Since DNA demethylation at master regulator genes such as *Ddx4, Hormad1* and *Dazl* is critical for maintaining the germ cell lineage [2], we analysed the promoter methylation of these genes in E15.5 ovaries. We found a tendency for increased DNA methylation at *Ddx4, Dazl* and *Hormad1* (*p* = 0.057 for *Ddx4* and *Dazl,* *p* = 0.1 for *Hormad1*) promoter CpG islands (Fig. 5), suggesting a possible impact of PB not only on histone modification levels, but also on DNA methylation levels. This DNA methylation increase could also reflect an alteration of the germline-to-somatic cell ratio, as these genes are normally highly methylated in somatic cells.

### Decreased oocyte number at E15.5 in PB ovaries

**Fig. 6:**
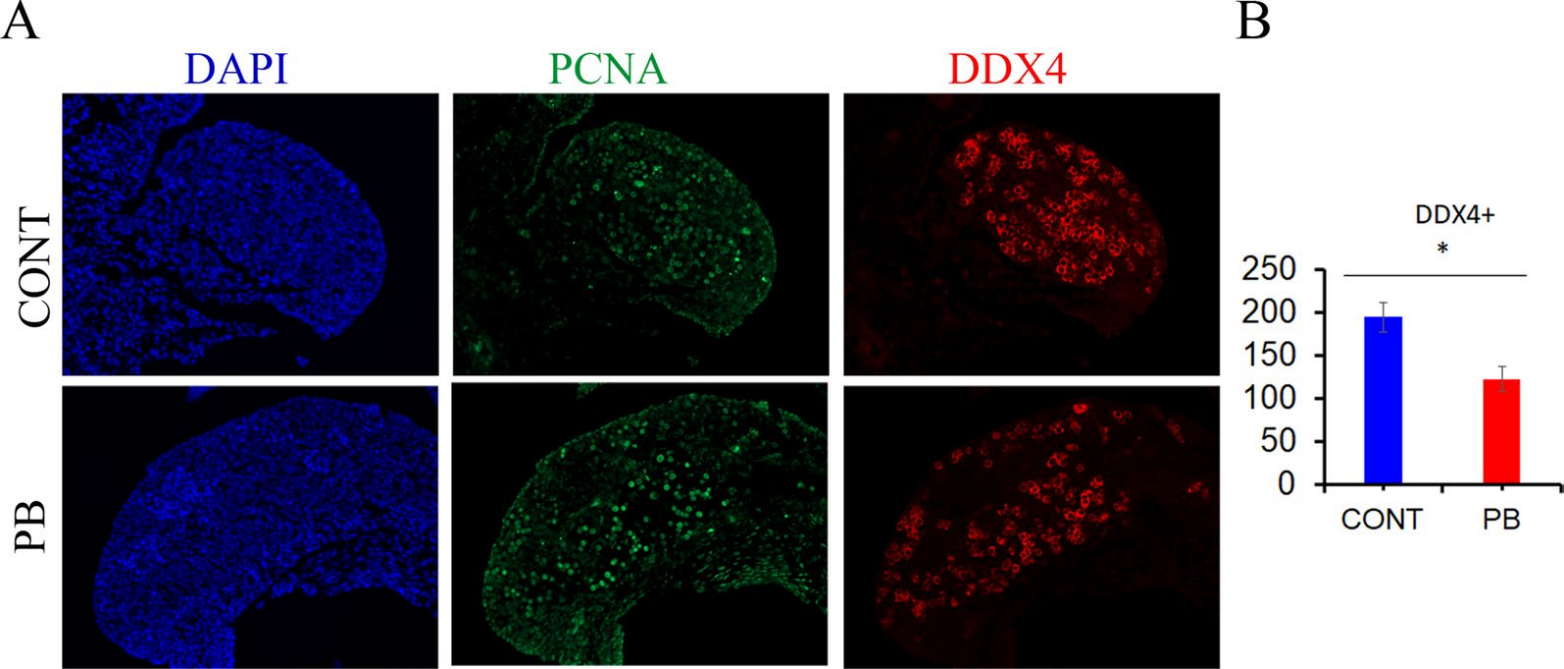
The germ cell number decreases upon PB treatment. **A** Paraffin sections of E15.5 ovaries were immunostained against DDX4 (red) and PCNA (green) in the control (top panel) and PB (bottom panel) groups. **B** The germ cells were scored for 5 biological replicates. The scores were averaged and plotted, and the data are presented as the number of oocytes per section ± SEM, nonparametric Mann–Whitney test. PCNA staining was used to illustrate that the germ cells were undergoing HR in oocytes

Since SGT reprogramming is critical for the establishment of the germ cell lineage and because we observed alterations in DNA methylation at genes involved in the maintenance of the germ cell lineage, we counted germ cells in E15.5 ovary sections. We prepared paraffin sections from E15.5 ovaries and immunostained them for the germ cell marker DDX4 (Fig. 6A). Germ cell counting revealed a 30% decrease in oocyte number at E15.5 (*p* < 0.05) (Fig. 6B), suggesting a possible deleterious effect of PB exposure on the germline cell population.

### Increased follicle growth at PND5 in PB-exposed ovaries

**Fig. 7:**
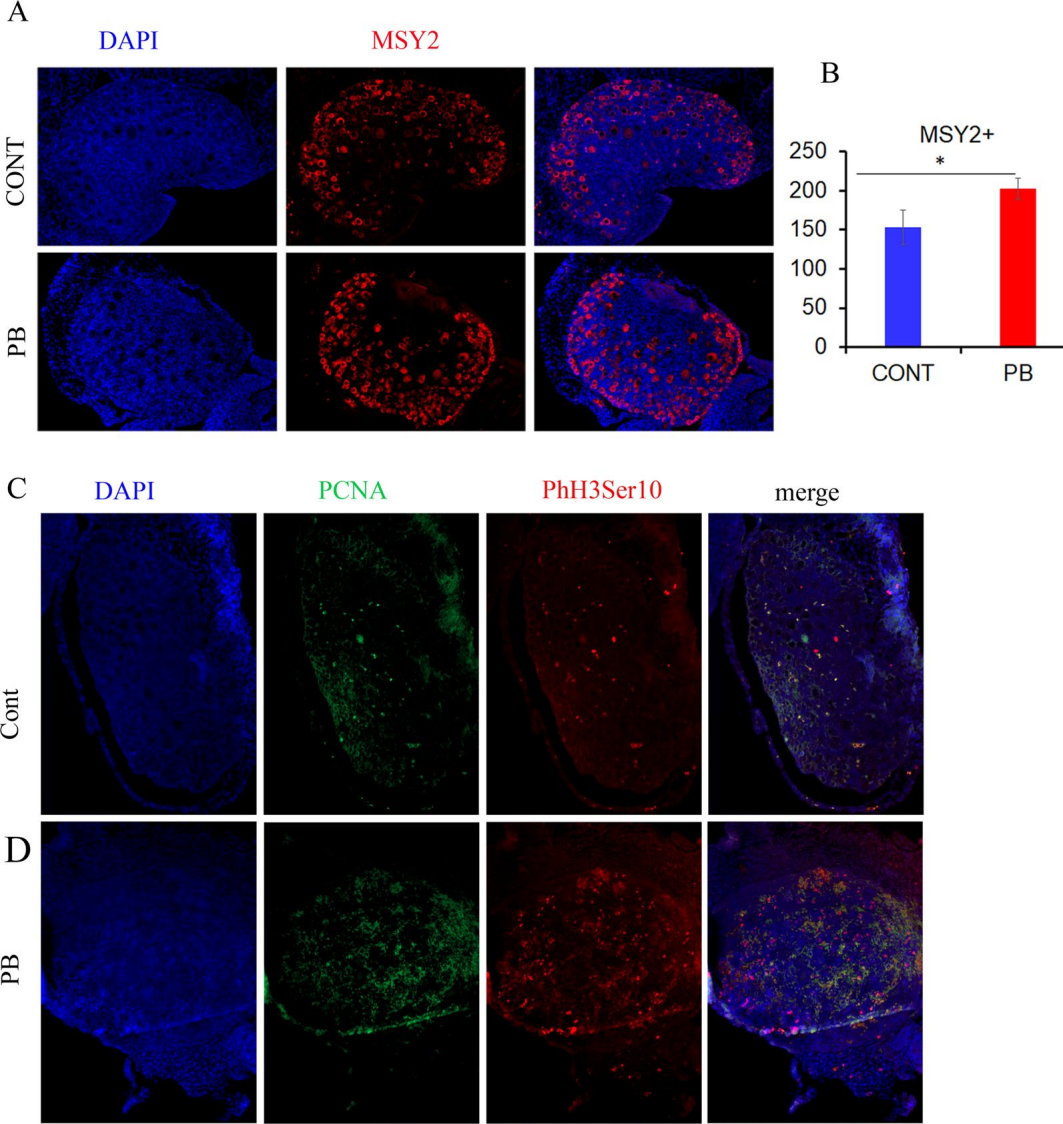
Increased proliferation in PB-exposed PND5 ovaries. **A** Paraffin sections of PND5 ovaries were immunostained for MSY2 (red) in the control (top) and PB (bottom) groups. **B** The follicles were scored for 5 biological replicates. The scores were averaged and plotted and are presented as the number of follicles per section ± SEM; *p < 0.05, nonparametric Mann–Whitney test. **C** Representative image of paraffin sections of PND5 ovaries immunostained for PCNA (green) or phosphorylated histone 3 at serine 10 (red) in the control (top) and PB (bottom) groups. Since PCNA and phosphorylated -histone H3 at serine 10 showed heterogeneity in cell type staining, quantitative analysis of fluorescence-positive cells was not performed

To check the follicle maturation efficiency, we immunostained PND5 ovary sections with an oocyte marker, MSY2, which is a cytoplasmic marker of germ cells. At this timepoint, MSY2 stained mainly primary and secondary follicles, which are oocytes surrounded by one or two layers of somatic granulosa cells (Fig. 7A). We detected an increase in MSY2-positive cells in the PB group (Fig.  7B), suggesting that more oocytes were undergoing maturation in the PB group than in the control group. To confirm our observation, we coimmunostained the ovaries for the proliferation marker PCNA and the mitosis marker phospho-histone H3 (serine 10) (Fig. 7C). We observed that both markers had increased signal intensity at PND5, confirming the proliferation of somatic cells.

**Fig. 8:**
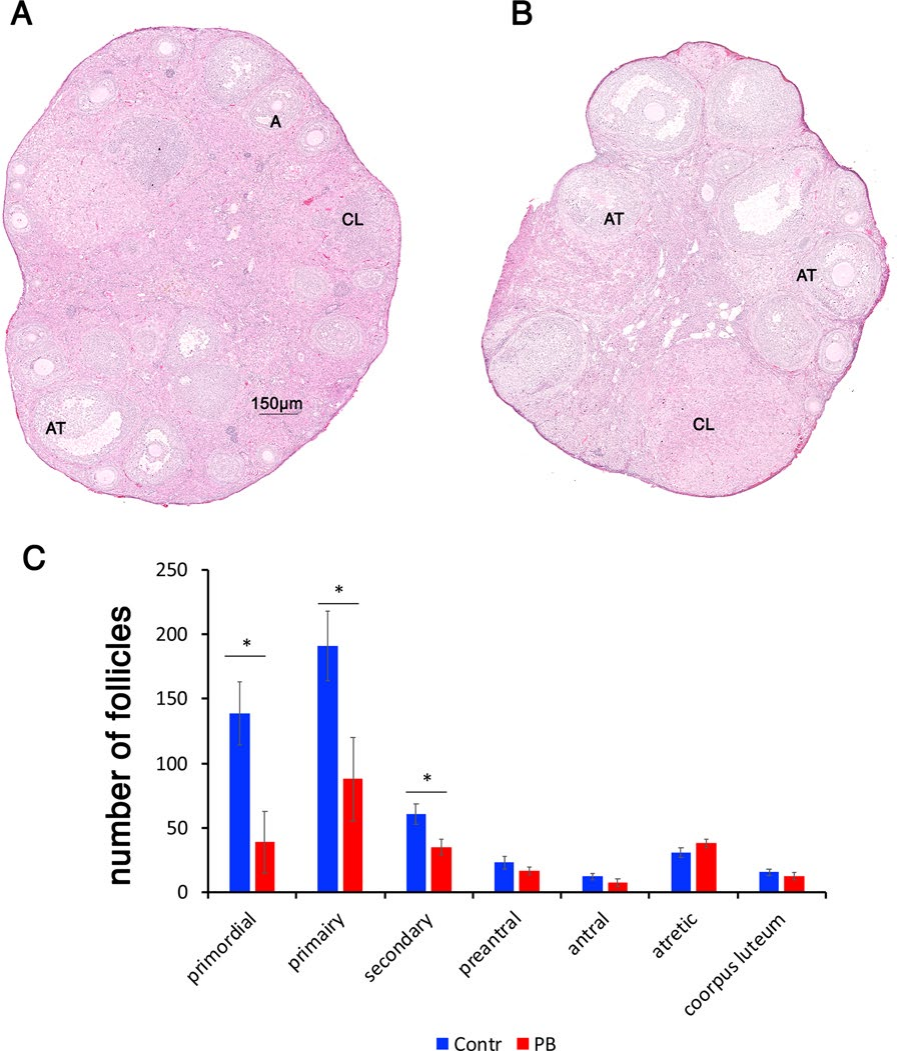
PB-induced morphological changes in adult ovaries. Representative H&E-stained images of ovaries in control (**A**) and PB-treated (**B**) samples (NanoZoomer; 5X magnification). In control ovaries, most of the late follicles were healthy; in contrast, in treated ovaries, decreases in primordial, primary and secondary follicles were observed. **C** Quantitative analysis of follicle numbers. The follicles were counted manually using NDP.view2 software and were categorized according to the classification defined in the Methods sections. We scored follicles in 4 biological replicates for the control and treated groups. The total numbers were compared; *n* = 4 for each group; *p < 0.05, nonparametric Mann–Whitney test. AT, atretic follicle; A, normal antral follicle; CL, *corpus luteum*

To determine whether gestational exposure to PB affects the follicular population in adult mice, we analysed histological sections of ovaries from 5-month-old mice (Fig. 8A, B) and scored the different follicle types as described in the Methods section. Analysis of the follicle counts (Fig. 8C) showed a 3.6-fold decrease in primordial follicles (139 ± 24 control, 39 ± 24 treatment, *p < 0.05, *t*-test), a 2.2-fold decrease in primary follicles (191 ± 27 control, 88 ± 32, treatment, *p < 0.05, *t*-test) and a 1.7-fold decrease in secondary follicles (61 ± 8, control, 35 ± 6 treatment, *p < 0.05, *t*-test, minimum 4 animals for each group).

### The transcriptional network is perturbed in adult mouse ovaries following gestational PB exposure

**Fig. 9:**
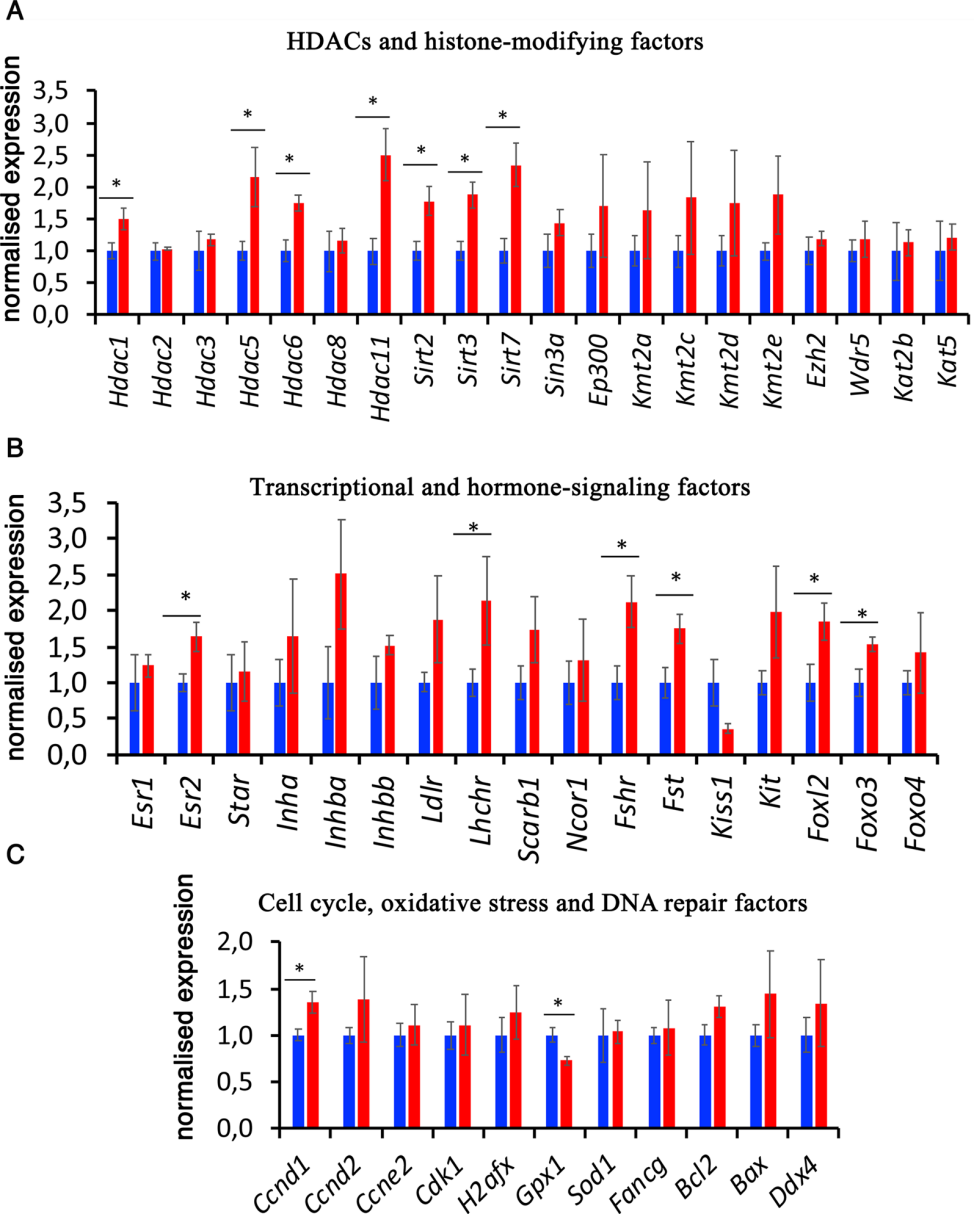
Gene expression in adult ovaries. Gene expression was analysed by RT-qPCR using RNA from ovaries from 5-month-old animals. The expression of genes was normalized to the expression of the housekeeping gene *Rpl37a* and is presented as the average signal compared to the control ± SEM; *p < 0.05, nonparametric Mann–Whitney test; *n* = 5 for the control and PB groups. The RT-qPCR primers are listed in Additional file 1: Table S2

We performed RT-qPCR gene expression analysis on the ovaries of adult mice that were exposed to PB as embryos. Several candidate genes were selected, including HDACs, histone-modifying enzymes, germ cell markers, genes involved in the oestrogen signalling pathway and genes involved in the transcriptional regulation of ovarian development (*Foxl2, Foxo3).* We also examined the expression of genes that are involved in the oxidative stress response (*Gpx1, Sod1)* and DNA damage (*H2afx*). We found that the expression of the genes encoding *Hdac1, Hdac5, Hdac6, Hdac11*, *Sirt2, Sirt3* and *Sirt7* increased following in utero PB treatment (Fig. 9A). We also found that the *Esr2, Lhchr, Fshr, Fst, Foxl2* and *Foxo3* genes were upregulated in the ovaries of in utero-treated mice (Fig. 9B). The expression of other histone-modifying enzymes was not significantly affected. The expression of *Gpx1*, a gene encoding a detoxification enzyme, was reduced in exposed ovaries (Fig. 9C). Our data show that in utero exposure to PB leads to gene expression alterations.

### Decreased H3K4me3 marks in adult oocytes following in utero PB exposure

**Fig. 10:**
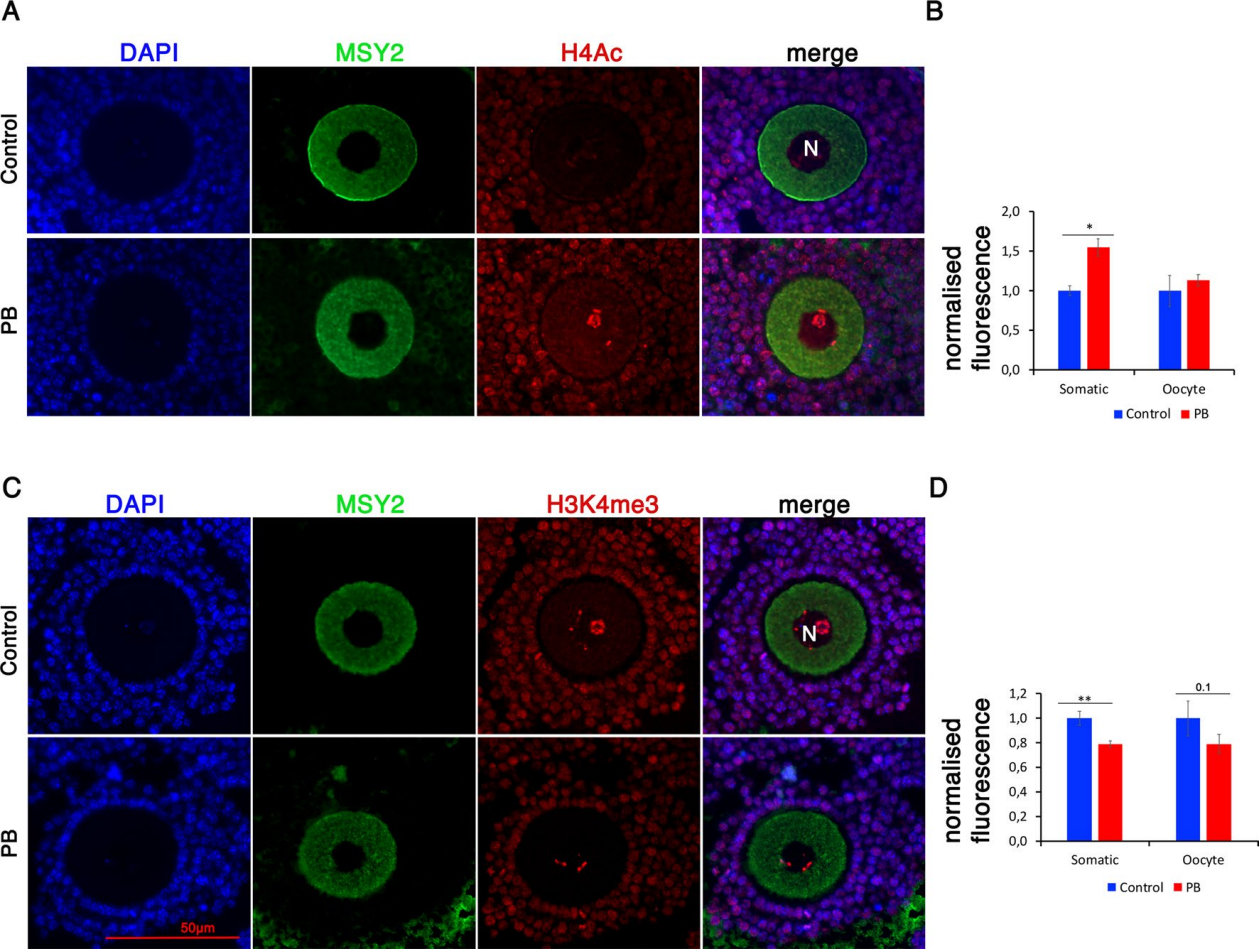
Decreases in histone H4 acetylation and H3K4me3 levels in adult ovaries following gestational PB exposure. Exposure to PB affects histone H4Ac and H3K4me3 levels in grown oocytes in adult ovaries. **A** Representative images of control (top) and PB-treated oocytes (bottom) immunostained for MSY2 (oocyte marker, green) or H4Ac (red) (40X magnification). **B** Quantitative analysis of the H4Ac signals in oocytes and in the surrounding granulosa cells; *n* = 4 for each group; *p < 0.05, nonparametric Mann–Whitney test. **C** Representative images of antral follicles immunostained for H3K4me3 (red) and MSY2 (oocyte marker, green). **D** Quantitative analysis of H3K4me3 signals in granulosa cells and oocytes from control and PB-treated samples; *n* = 4 for each group; p** < 0.01, nonparametric Mann–Whitney test

To determine whether gene expression is associated with alterations in epigenetic marks in adult ovaries, we immunostained ovary sections using antibodies against the important histone marks H4Ac and H3K4me3 (Fig. 10A, C). Since the signal for both histone marks was detectable only in the nuclei of fully grown oocytes, we analysed this mark in fully grown oocytes and in surrounding granulosa cells. Quantitative analysis of immunofluorescence of H4Ac showed a significant 1.5-fold increase in this marker in surrounding granulosa cells of fully grown oocytes, but we did not observe significant changes in oocytes themselves (Fig. 10B).

Quantitative analysis of the H3K4me3 mark showed a 1.3-fold decrease in H3K4me3 marks in surrounding granulosa cells, but no significant changes could be observed in the oocytes of treated ovaries (Fig. 10D).

In conclusion, our analysis revealed that gestational exposure to PB results in changes in gene expression in adult ovaries that are accompanied by an increase in H4Ac and a decrease in H3K4me3 in granulosa cells that surround fully grown oocytes. However, these changes in the levels of key epigenetic marks are not observed in fully grown oocytes.

## Discussion

### Exposure to PB affects meiosis

Given the potential usefulness of PB in human cancer therapy, understanding the mechanisms by which PB affects the female germline is of great importance. In this study, we found an increase in histone acetylation marks following PB exposure, which was expected since PB is an HDAC inhibitor. However, deregulation of the acetylation and deacetylation balance could affect other histone marks, in particular via changes in the regulation of histone-modifying enzymes. For example, we observed a decrease in the histone H3K4 demethylase factor KDM5A, suggesting that an increase in H3K4me3 could reflect decreased demethylation rather than an increase in lysine methyltransferase activity. KDM5A is physically and functionally associated with two histone deacetylase complexes [30]; therefore, HDAC inhibition could influence H3K4me3 levels. Acetylation and methylation are both involved in synaptonemal complex (SC) formation, and their altered levels in PB-treated mice may explain the delay in SC formation at E15.5. Moreover, PB-induced disturbances in the female sex-determination pathway could also lead to delayed entry into meiosis.

We observed the persistence of unrepaired DSBs in PB-exposed animals. Chromatin condensation after hyperacetylation is important for DNA repair [24]. Since acetylation involves a prolonged chromatin relaxation state, we speculate that it could lead to inefficient DNA repair. It has been shown that hyperacetylated chromatin during mitosis impairs proper chromosome condensation during the pre-anaphase stages, resulting in poor sister chromatid resolution [31]. We suggest that similar effects could impact meiosis in PB-exposed ovaries. The decrease in H3K9me3, shown to be associated with chromosome pairing failure [17], could also alter the orientation of chromosomes and delay DNA repair in PB-exposed ovaries.

In addition, exposure to PB, a nonselective HDAC inhibitor, could also affect the activities of HDACs that are directly involved in DNA repair. It has been shown that depletion of either HDAC9 or HDAC10 specifically inhibits homologous recombination [32]. On the other hand, we observed that DNA repair genes such as *Brca1* and *Wrn* (which are both known to be important for homologous recombination) showed decreased expression upon PB exposure. Since we did not analyse gene expression separately in somatic and germ cells, we cannot exclude the possibility that the observed alterations in DNA repair genes were present in somatic cells.

Altogether, the findings reveal that the chromatin state, effects on DNA repair genes and inhibition of HDACs involved in HR could lead to persistence of DSBs. We could not exclude that the presence of high number of DSBs is due to delayed entry into meiosis. Further work is required to answer this question. Since the persistence of DSBs has strong therapeutic potential, as it allows the specific elimination of rapidly growing cancer cells, further work is required to reveal the roles of other DNA repair factors perturbed by PB. We identified DNA repair factors in our RNA-seq data, including *Chek2, Huwe1, Neil1, Palb2, Paxip1, Polb, Recgl, Tex15,* and *Trp53bp1*, which should be further investigated.

### Consequences of PB exposure on gene expression

Since we did not observe alterations in HDAC gene transcription levels, we believe that the observed PB effects are a direct or indirect consequence of HDAC enzyme inhibition. On the other hand, we observed that more than 100 genes encoding Zn-finger proteins were deregulated. Since PB can directly bind to Zn [33], it is conceivable that PB affects the expression of genes encoding proteins that use Zn for their functional activity.

It has been shown that panobinostat directly affects HDAC1, HDAC2, HDAC6 and TTC38 [34] and some other targets such as zinc finger E-box-binding homeobox 1 (ZEB1) and 2 (ZEB2) which are EMT master regulators [33] suggesting that zinc finger proteins are also targets of PB. Since the dose we used was higher than patients take, the alteration of many Zn-finger proteins could be also due to unspecific cellular response to high PB level.

In our study, we detected an increase in *Hdac7,* which plays a key role in the maintenance of vascular integrity and has important functions during cardiovascular development [35]. This unexpected increase might reflect the induction of a feedback loop upon inactivation of the HDAC7 protein, possibly due to deficiency in Zn.

Further work is required to investigate this possibility. The alterations in germ cell differentiation following PB exposure could be due to perturbation in somatic-to-germline transition accompanied by global demethylation and establishment of a new epigenetic state at the so-called germline reprogramming-responsive (GRR) genes [2]. The changes in DNA methylation at master regulator genes (e.g., *Ddx4, Dazl, Hormad1*), which are essential for germ cell lineage establishment, support our hypothesis.

How can alterations in these genes affect ovary development? DAZL (deleted in azoospermia-like), a germ cell-specific RNA-binding protein, is essential for developing PGCs [36]. Since DAZL is also a meiosis-promoting factor in developing germ cells [37], the perturbation in this factor could affect entry into meiosis. *Ddx4*, also known as Vasa, a Drosophila Vasa orthologue, is a conserved germline factor in metazoans [38]. *Ddx4* plays an essential role in germ cell determination [39]. We suggest that alterations in DDX4 may perturb germ cell specification. *Hormad1* is essential for normal synaptonemal complex formation and for the efficient recruitment of ATR checkpoint kinase activity to unsynapsed chromosomes [40]. Thus, changes in *Hormad1* could affect normal meiosis progression.

Other factors expressed in somatic tissue are necessary for female gonad development. For example, RSPO1 activates the WNT4/β catenin pathway to oppose male sex development [41]. The transcription factor *Foxl2* is expressed in ovarian granulosa cells from sex determination to adulthood [42]. *Foxl2* expression prevents normal testis development in transgenic mouse embryos with ubiquitous *Foxl2* expression [43]. In our study, we identified alterations in the expression of many developmental genes regulated by sex-determination factors expressed in somatic cells in males, such as SOX9 and DMRT1, suggesting that PB exposure strongly deregulates the female-specific transcriptional network.

In adults, we found that the expression of genes that are normally expressed in granulosa cells, such as *Esr2, Lhchr, Fst* and *Fshr*, was altered due to gestational PB exposure. This change in gene expression could have been a consequence of increased ovarian somatic cell proliferation after birth. However, we cannot exclude the possibility that the change in gene expression was a consequence of changes in cellular composition. We also suggest that the observed epigenetic changes in granulosa cells in adult ovaries could have contributed to these alterations.

### Embryonic PB exposure affects the germ cell population

In this study, we also examined the effects of PB treatment on the ovarian cell population. We observed a decrease in germ cell number at E15.5 following PB exposure. This can be explained by the effects of HDACs on the cell cycle. Prior to meiosis, oogonia mitotically divide to form up to 25,000 cells [44]. Since we counted the germ cells at E15.5, it is unlikely that the cells were eliminated due to meiotic failure, as synaptonemal complexes were not yet formed and meiosis did not progress. Since the inhibitory effects of PB on mitosis have been previously reported [45], the observed decrease in germ cell population could have also been due to impaired mitosis prior to meiosis onset.

In PND5 ovaries, we detected increased numbers of growing primary and secondary follicles together with increases in PCNA and phospho-histone H3 (serine 10) signals, suggesting that the cells were massively proliferating. This could have been a consequence of *Foxo3* expression deregulation in PND5 mice. Indeed, it has been shown that in *Fox*o3 KO mice, massive uncontrolled activation of follicles occurs such that the mouse ovaries become deficient in the entire pool of primordial follicles because they have been prematurely activated [46]. In our study, *Foxo3* showed a 2.3-fold decrease in expression in PB-exposed mice. This low *Foxo3* expression may explain the cell proliferation in primary and secondary follicles.

We also observed decreases in primordial, primary and secondary follicles in 5-month-old mice. This decrease could be explained by the poor initial survival of germ cells due to embryonic exposure to HDAC inhibitors and premature activation after birth. These two hypothetical pathways are not mutually exclusive and may both have contributed to reducing the oocyte stockpile observed in 5-month-old animals.

## Conclusion

Our data suggest that inhibition of histone deacetylases by PB during a critical developmental window affects histone epigenetic marks to in turn impair meiosis and eventually lead to ovarian oocyte reserve shrinkage. A schematic presentation of a possible mechanism of action of PB on the ovarian system is presented in Additional file 1: Fig. S9. In this schema, we suggest that impairment of the sex-specific programme, such as unprogrammed expression of male-specific genes and a simultaneous decrease in female-specific gene expression, could contribute to global defects in PB-exposed female ovaries.

## Methods

### Animal treatment

Inbred C57BL/6J mice were used for all experiments. We used a dose of 5 mg/kg/of PB on alternate days. This dose is 17.5 times higher than that administered to humans for therapy (20 mg per day for 21 days). This dose is somewhat high; however, in our study, the drug was given only 5 times, while humans take it for a longer period; in addition, in humans, it is often combined with bortezomib and dexamethasone. In animal studies, several doses have been tested (10, 30, and 100 mg/kg/day), and it has been shown that 10 mg/kg/day can cause some effects on reproductive functions (https://dailymed.nlm.nih.gov/dailymed/drugInfo.cfm?setid=7774972a-eeaa-4b9a-9e56-3fc1b968e86a), such as embryo resorption, which we did not observe in our study. We also did not observe changes in litter size or body weight in 5-day-old mice.

The exposure window was from E6.5 to E15.5, which corresponds to the somatic-to-germline transition. Briefly, the drug was first dissolved in DMSO and then diluted in water (1:30) and administered via oral gavage in a volume of 150 μl. The drug was administered every other day, similar to the regimen used in humans for cancer treatment. Control mice were treated with the same volume of DMSO and water. Ovaries were dissected at E15.5, E17.5, E18.5, PND5 and the adult stage (5 months). The ovaries were fixed in Bouin’s solution or flash-frozen in liquid nitrogen. Embryonic ovaries were used for the preparation of surface spreads or structurally preserved nuclei or were flash-frozen in liquid nitrogen for RNA extraction. For most of the experiments, a minimum of 4 animals from separate litters were used.

### Preparation and immunostaining of structurally preserved nuclei

Structurally preserved nuclei (SPNs) for three-dimensional analysis were prepared by cutting fresh E15.5 or E18.5 ovarian tissues in DMEM (Life Technologies, Gibco) with 0.5% protease inhibitor. The ovary suspensions were mixed with equal volumes of 3.7% (vol/vol) paraformaldehyde and 0.1 M sucrose and spread on glass slides. The slides were then air-dried and stored at − 80 °C. The slides were washed several times with PBS before use and for 2 min with 0.1 M glycine in PBS to remove paraformaldehyde traces. The slides were permeabilized for 30 min in PBS/0.5% Triton at room temperature (RT), washed with PBS and blocked for 30 min in a solution containing 0.1% (v/v) donkey serum, 0.03% (w/v) BSA, and 0.005% (v/v) Triton X-100 in PBS. SPNs were coimmunostained with rabbit polyclonal H4Ac (1:500, Merck Millipore, 06-956), rabbit monoclonal KDM5A (1:500, Abcam, ab194286) or rabbit H3K9me3 (1:500, Abcam, ab8898) and mouse SYCP3 (1:200, Santa Cruz, SC-33195) antibodies at 4 °C overnight; washed several times; incubated with fluorescent Alexa Fluor-conjugated secondary antibodies; and mounted with VECTASHIELD solution (Vector Laboratories, Burlingame, CA) containing 0.001% (v/v) 4,6-diamidino-2-phenylindole dihydrochloride (DAPI). Z-stacks were acquired with a 500-nm step, and 21 individual planes were taken for each individual channel for DAPI (blue, 350 nm), SYCP3 (red, 594 nm) and H4Ac (green, 488 nm) staining using the Zen Pro (version 2.3) program. All images for control and exposed samples were taken with a fixed exposure time. Deconvolution was performed using the “Fast Iterative” algorithm provided by Zen Pro. The sum of image intensities was generated for each z-stack, and the resulting fluorescence was calculated in ImageJ v1.52n. We used the lasso tool for nucleus and cytoplasm contouring, and the integrated density immunofluorescence for each nucleus was calculated. To ensure proper signal quantification, a background area was subtracted from each raw signal. We used 4 independent biological replicates for most of the experiments, and a minimum of 15 cells for each replicate were analysed. The data were plotted in MS Excel and are presented as corrected total cell fluorescence (CTCF) for nuclei compared to the control ± SEM, *p < 0.05, **p < 0.01.

### Meiotic chromosome surface spreads

The ovaries were dissected at E15.5, E17.5, and E18.5 and kept in ice-cold PBS. The ovaries were placed in a drop of 100 mM sucrose (20 µl), carefully disrupted with tweezers and pipetted until a cell suspension was formed. The cells were then transferred onto a glass slide with 100 µl of 1% paraformaldehyde containing 0,1% (v/v) Triton X-100, kept for 2–4 h in a humidified chamber at RT, and then dried. The slides were washed 4 times for 1 min each in 0.4% (v/v) Kodak Photo-Flo solution, air-dried and kept at -80 °C until use.

### Immunolabelling of ovarian spreads

The ovarian spreads were blocked with blocking buffer (0.1% (v/v) donkey serum, 0.03% (w/v) BSA, and 0.005% (v/v) Triton X-100 in PBS) for 20 min at 37 °C in a humidified chamber. The slides were then incubated with a primary antibody diluted in blocking solution for 1 h at 37 °C in a humidified chamber, washed three times with 0.4% (v/v) Kodak Photo-Flo/PBS and incubated with a secondary fluorescent Alexa Fluor-conjugated antibody diluted 1:500 for 20 min at RT. The slides were washed three times with 0,4% (v/v) Kodak Photo-Flo/PBS and mounted with VECTASHIELD mounting medium containing 5 µg/ml DAPI. The slides were analysed with an epifluorescence Axio Imager M1 microscope (ZEISS, Germany), and pictures were taken using a 63X or 100X objective with AxioVision Rel 4.7.1 software provided with microscope. For DMC1 foci visualization, slides containing E15.5 and E17.5 and E18.5 cells were incubated with primary rabbit anti-DMC1 (1:100, Santa Cruz, SC-22768) and mouse anti-SYCP3 (1:100, Santa Cruz, SC-74569) antibodies. DMC1 foci were counted manually using Photoshop, and the average number of foci per cell was compared between the control and treated groups. For H3K4me3 visualization, slides containing E18.5 ovaries were incubated with primary rabbit anti-H3K4me3 (1:500, Millipore, 07-473) and mouse anti-SYCP3 (1:100, Santa Cruz, SC-74569) antibodies. For H3K9Ac visualization, slides containing E18.5 ovaries were incubated with primary rabbit anti-H3K9Ac (1:500, Millipore, 07-352) and mouse anti-SYCP3 (1:100, Santa Cruz, SC-74569) antibodies. We used a minimum of 4 biological replicates for the control and treated groups derived from 4 different litters. We analysed at least 20 cells for each biological replicate and used ImageJ for quantitative analysis of the immunofluorescence of histone marks. We measured the intensity of fluorescence by contouring the nuclei with the lasso tool of ImageJ. The signals for histone marks, SYCP3, DAPI and the background were measured. We calculated the CTCF (corrected total cell fluorescence) as the product of the mean value of fluorescence (with background subtracted) and the cell area. The immunofluorescence signal was normalized to the DAPI signal, and the data are presented as the normalized fluorescence compared to the control.

### Immunofluorescence on paraffin sections

For immunostaining, the ovaries from the control and PB-treated groups were fixed in 4% (w/v) PFA solution for 16 h, dehydrated and embedded in paraffin. The sections were deparaffinized and rehydrated, and the epitopes were unmasked in 0.01 M citrate buffer, pH 6, at 80 °C for 45 min. After washing in 1X PBS-0,05% Tween (PBS-T), the sections were incubated with rabbit anti-H3K4me3 (1:500, Merck Millipore, 07-473) and mouse anti-MSY2 (1:500, Santa Cruz, SC-3938440) antibodies, rabbit polyclonal anti-hyperacetylated histone 4 (1:500, Merck Millipore, 06-956) and mouse anti-MSY2 (1:500, Santa Cruz, SC-3938440) antibodies, mouse anti-PCNA (1:500, Abcam, ab29) and rabbit anti-phospho-histone (serine 10) (1:500, Millipore, 06-570) antibodies, or rabbit polyclonal anti-DDX4 (1:500, Abcam ab13840) and mouse anti-PCNA (1:500, Abcam, ab29) antibodies. The sections with primary antibodies were incubated in PBS-T overnight at 4 °C in a humidified chamber. After washing in PBS-T, the sections were incubated with appropriate fluorescent secondary antibodies (1:100) for 1 h in a humidified chamber at room temperature. The sections were counterstained with 0.001% (v/v) 4,6-diamidino-2-phenylindole dihydrochloride (DAPI) and mounted using VECTASHIELD solution. Images were obtained using an AxioImager microscope equipped with an AxioCam MRc5 camera and AxioVision software version 4.8.2 (Zeiss, Germany) with a 40X objective lens. We analysed IF intensity in antral follicles only. For the analysis, we chose oocytes with nuclei of similar size. We analysed a minimum of 4 follicles per replicate and separately quantitated the signal intensity in oocytes and surrounding granulosa cells using ImageJ v1.52n. We subtracted the background using regions with no cells. The mean intensity of H3K4me3 or H4Ac signals was measured, normalized to the signal intensity of the oocyte marker MSY2 and calculated as the normalized mean fluorescence compared to the control.

### Quantification of germ cells and follicles

Quantification of germ cells was performed at E15.5 and PND5 on paraffin sections using antibodies against the germ cell markers DDX4 (E15.5) and MSY2 (PND5). We scored the average number of germ cells per section. For the analysis of adult follicles, ovaries were fixed in Bouin solution, washed in PBS and 70% ethanol, dehydrated and embedded in paraffin. Five-micrometre sections of the entire ovaries were cut, and every 5th section was placed on the slide. The sections were deparaffinized, rehydrated and stained with PAS-haematoxylin. The follicles were quantified using NDP.view2 software. The follicles were counted when the nuclei of the oocytes were visible in the follicles. The follicles were classified as primordial when each oocyte was surrounded by a single layer of flattened granulosa cells. as primary when at least one of the granulosa cells of the single layer was cuboidal, as secondary/preantral when the number of granulosa cell layers exceeded one, and as antral when the antrum cavity appeared. The follicles were classified as atretic when they contained at least 2 pyknotic granulosa cells. We analysed on average 46 sections per control and 43 sections per PB-treated sample.

### DNA methylation analysis

Three to four E15 embryonic ovaries were pooled for one biological replicate, 4 biological replicates for the control and PB groups were prepared, and DNA was extracted according to the DNAeasy protocol (Qiagen, 69,506). An optional RNase A treatment step was included. Nearly 1000 ng of DNA was extracted for each biological replicate. For DNA methylation analysis, the EpiMark Methylated DNA Enrichment Kit (NEB, #E2600S) was used. 500 ng of DNA was sonicated using a Qsonica sonicator with the following parameters: efficiency 60%, total sonication time 6 min, 20 s on and 20 s off. Sonicated methylated DNA was precipitated using beads, the methylated DNA-MBD2a-protein A-coated bead complex was washed, and DNA was eluted with elution buffer. The DNA concentration was determined by the fluorescent method using dsDNA-binding dye (Promega). 12–15 ng of methylated DNA was recovered following precipitation, and the unprecipitated starting sonicated material was used as the input. Equal amounts of methylated DNA and input were taken for qPCR using primers (Additional file 1: Table S2) located in the CpG island of each tested gene. A region in the *Rplpo* gene was used for background normalization. The methylated DNA-to-input ratios were averaged and compared between the control and PB groups. A nonparametric Mann–Whitney test was used to assess the statistical significance.

### RNA extraction and quantitative PCR

Total RNA was extracted using an RNeasy Plus Mini Kit (Qiagen) according to the manufacturer’s instructions. This kit includes a DNA elimination step. For embryonic ovary analysis, RNA was extracted from a pool of 4 embryonic ovaries. For RNA analysis in adult mice, 5 individual ovaries from mice in 5 different litters from each group were used. Reverse transcription was performed with 1 µg of RNA using an iScript™ cDNA Synthesis Kit (Bio-Rad). The resulting cDNA was diluted 10 times and used for quantitative RT-qPCR. The primer sequences used for RT-qPCR are shown in Additional file 1: Table S2. RT-qPCR was performed using iTaq Universal SYBR Green Supermix (Bio-Rad) according to the manufacturer**’**s instructions on a CFX384 Touch Real-Time PCR Detection System (Bio-Rad). The quantification cycle (Cq) values were calculated using Bio-Rad CFX Manager 3.1. The Cq values of *Rpl37a* cDNA were used for normalization. The data were analysed and are presented as the mean fold change (FC) values compared to the control ± SEM, *p < 0.05, **, *p* < 0.01, *** *p* < 0.001.

### RNA-seq analysis

Three biological replicates from control and embryonic ovaries were used for RNA-seq analysis, and each replicate was generated from a pool of a minimum of 8 ovaries. The pooled replicates generally showed reproducibility (Additional file 1: Fig. S10). The libraries were prepared using an NEBNEXT® Ultra™ II Directional RNA Library Prep Kit for Illumina® (NEB E7760S) using 1 µg of total RNA according to the manufacturer’s instructions. Library sequencing was performed on an Illumina HiSeq 4000 sequencer to produce paired-end 100-base reads. Image analysis and base calling were performed using RTA 2.7.3 and bcl2fastq 2.17.1.14. Adapter dimers were removed using DimerRemover. Quality control was assessed using FastQC 0.11.2. To analyse the differentially expressed genes (DEGs), first, the quality-checked reads were aligned to the reference genome (mm10 assembly) using HISAT2 (2.1.0) [47] with the –dta and –known-splicesite-infile options. Then, StringTie (1.3.5) [48] was used to assemble RNA-seq alignments into potential transcripts using the NCBI RefSeq gtf file as a reference annotation. Individual transcriptomes were then merged into a general nonredundant transcriptome using StringTie merge mode (–merge). Finally, we performed quantification of the transcriptome for each biological replicate, and we prepared the transcript count matrix, which is the required input for the DESeq2 package, using the prepDE python script available on the GitHub repository of StringTie. The read counts were normalized and compared between control and PB samples using DESeq2 (1.30.0) [49]. The p-values were automatically adjusted for multiple testing by DESeq2 using the Benjamini and Hochberg method. The adjusted p-values are hereafter referred to as the false discovery rates (FDRs) in this manuscript. Downstream analyses were performed on the DEGs, which were transcripts with a fold-change (FC) between the control and PB above 2 and an FDR below 0.05. Gene set enrichment analysis (GSEA) was performed using the web-based tool g:Profiler [50] with the default parameters. Heatmaps were generated with the R package Pheatmap (1.0.12) using row clustering (Euclidian distance, complete-linkage clustering) and row scaling (scaling and centering).

### Statistical tests

For most of our experiments, we used a nonparametric Mann–Whitney test, *p < 0.05, **p < 0.01, ***p < 0.001; for the follicle count, we used a *t*-test, *p < 0.05.

## Supplementary Information

**Additional file 1.** Additional Figures S1–S10 and Tables S1–S2.

## Data Availability

All RNA-seq data from this study are publicly available and have been deposited in the National Center for Biotechnology Information Gene Expression Omnibus, the GSE number is GSE174831.
