# Supplementary material for: Histone deacetylase inhibition leads to regulatory histone mark alterations and impairs meiosis in oocytes
Source: Epigenetics Chromatin. 2021 Aug 12;14:39. doi: 10.1186/s13072-021-00413-8 (PMC8359552; doi:10.1186/s13072-021-00413-8)
Supplement: Supplementary file 1 — Additional file 1. Additional Figures S1–S10 and Tables S1–S2. [file 13072_2021_413_MOESM1_ESM.pdf]

Histone deacetylase inhibition leads to regulatory histone mark alterations and impairs meiosis in oocytes.

Louis Legoff*, Ouzna Dali*, Elena de La Mata Santaella, Christian Jaulin, Shereen Cynthia D'Cruz#, Fatima Smagulova#

Univ. Rennes, EHESP, Inserm, Irset (Institut de recherche en santé, environnement et travail) - UMR_S 1085, F-35000, Rennes, France

***These authors equally contributed.**

# corresponding authors
Shereen Cynthia D'Cruz: shereen-cynthia.d-cruz-benard@inserm.fr
Fatima Smagulova, fatima.smagulova@inserm.fr

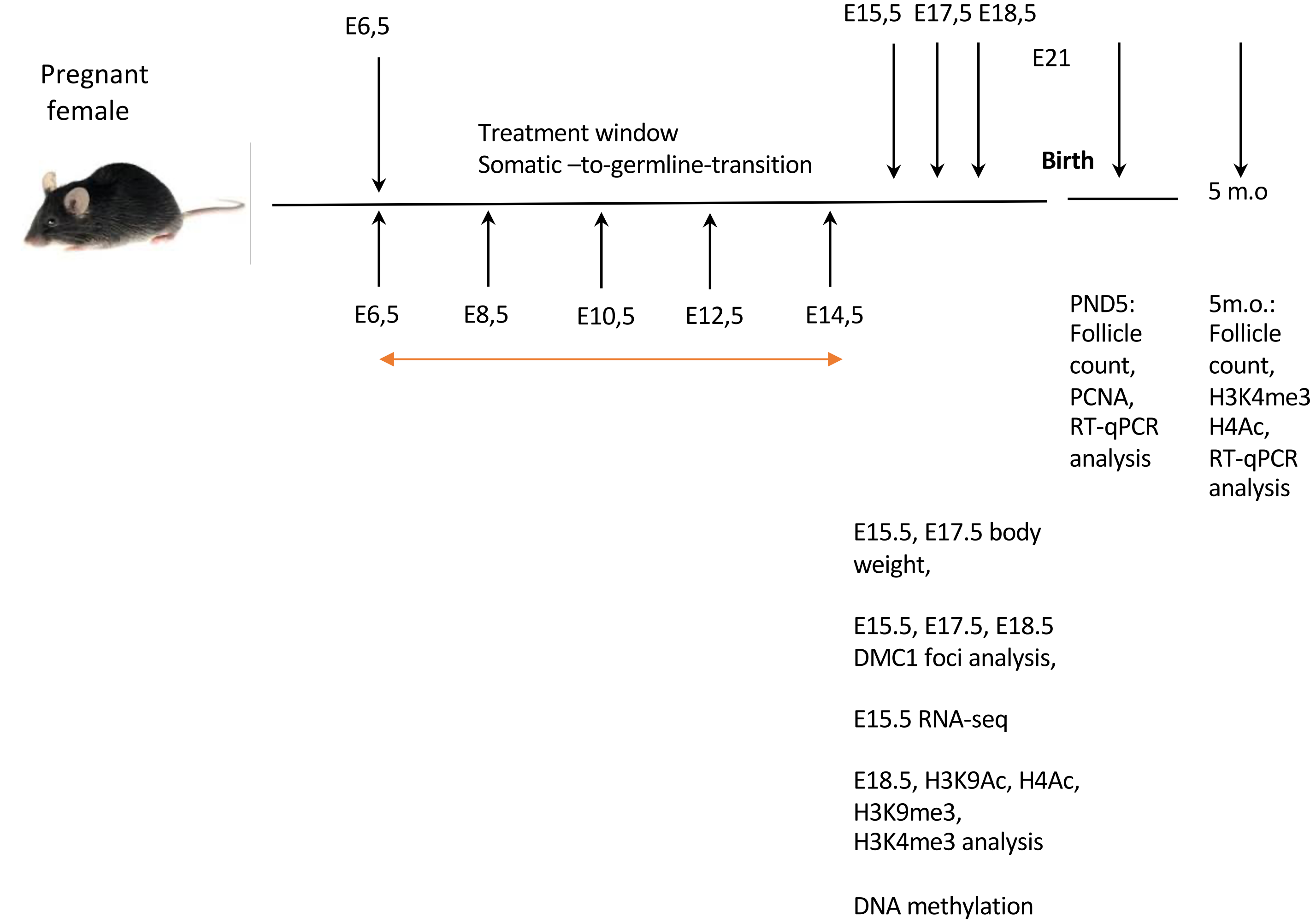

Additional file1: Fig.S1. Schematic representation of the experimental design. Mice were treated at E6.5-E15.5 with a 5mg/kg/alternate day of PB diluted in water and DMSO, control mice were treated with water and DMSO.

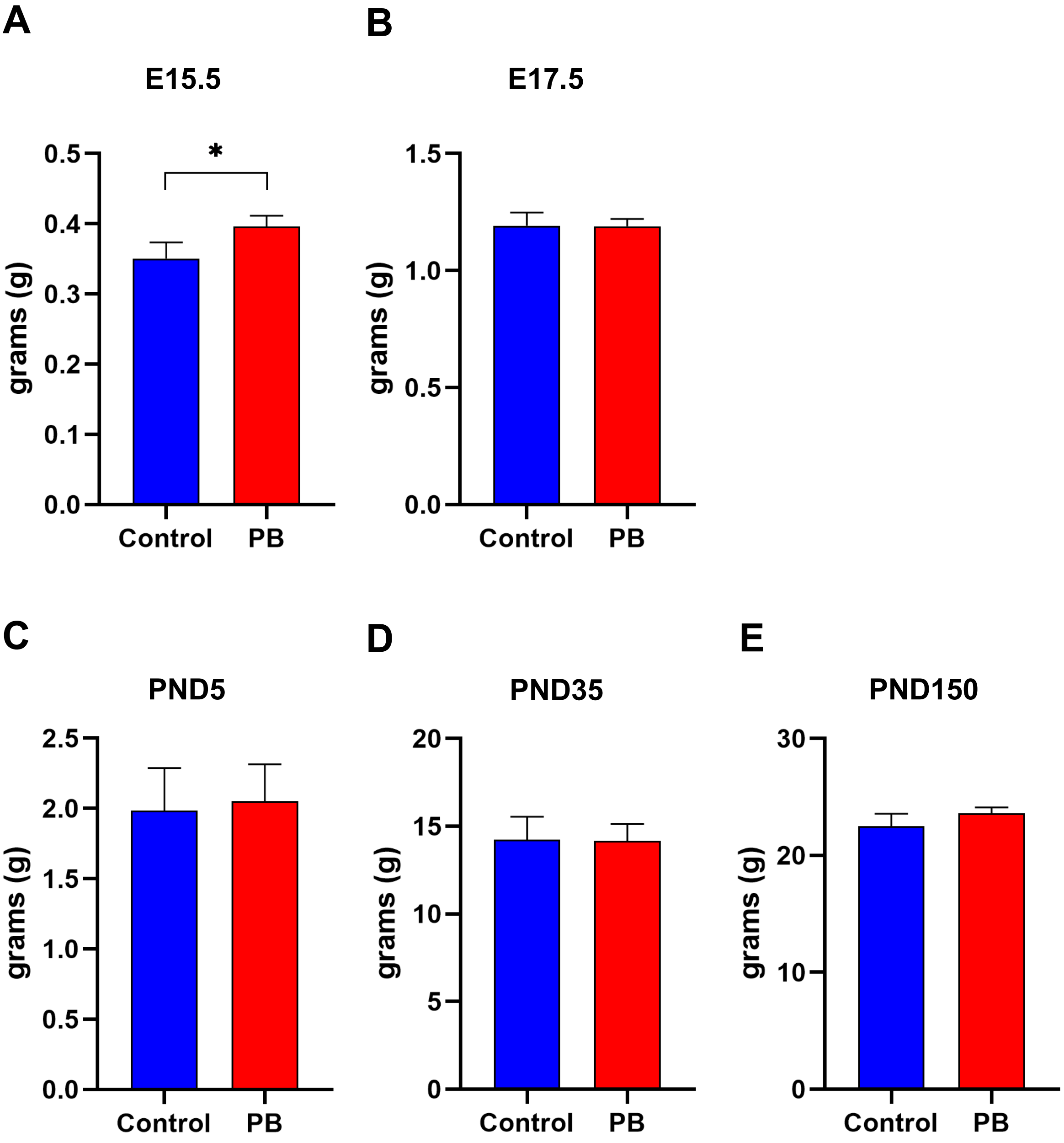

Additional file1: Fig.S2. Body weight.
(A) E15.5 mice. Control n=6, PB n=4. (B) E17.5 mice. Control, n=4, PB=5. (C) PND5 mice. Control n= 6, PB=6. (D) PND35 mice. Control n=12, PB=16. (E) PND150. Control n=4; PB=4. Wilcoxon-Mann-Whitney test.

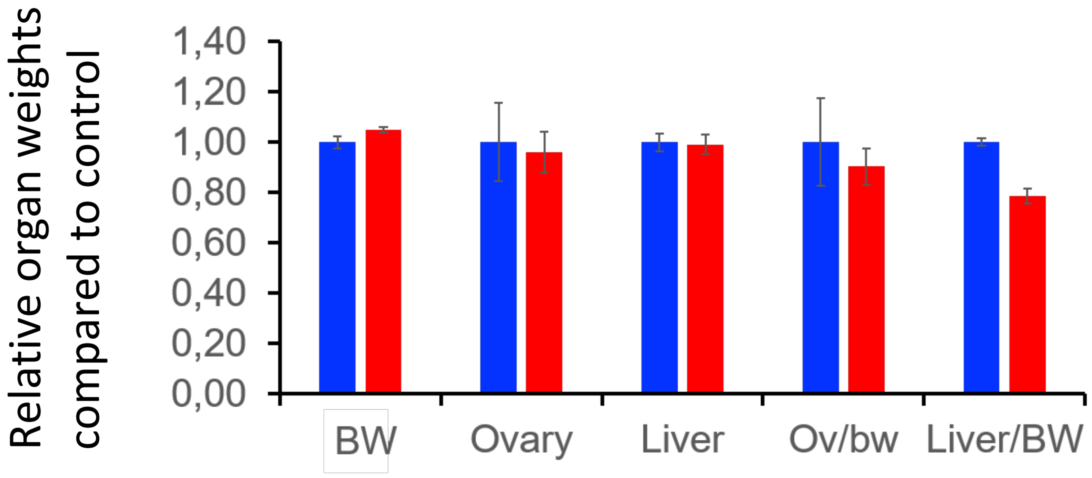

Additional file1: Fig.S3. Effects of PB exposure on body and ovary weights in embryo and in adults. BW, body weight, Ov/bw, Liver/BW ovary or liver weight ratios to body weight ratio compared to control. Control n=12, PB n=16, weight is given in grams.

A

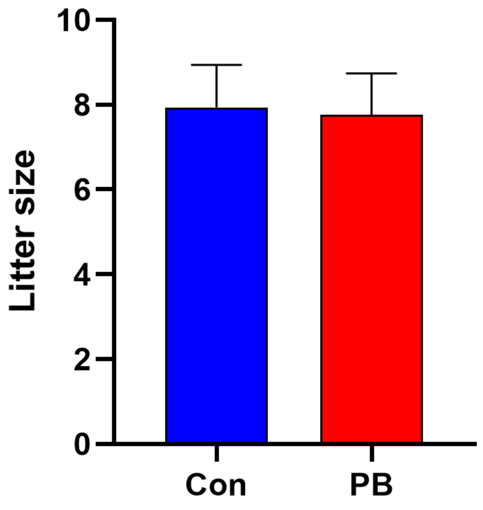

B

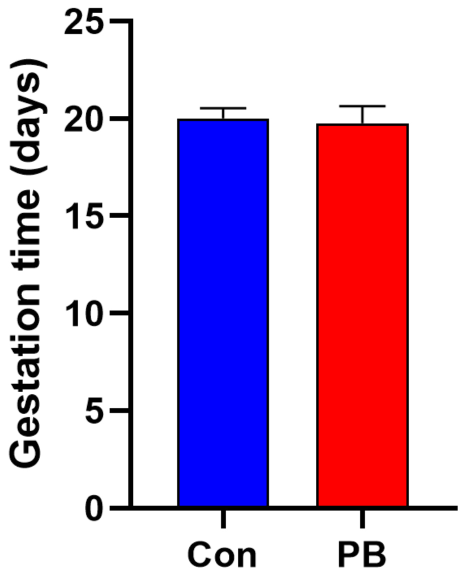

Additional file1: Fig.S4. PB exposure does not affect gestation parameters.
(A) Litter size. Control n=16, PB=17. (B) Gestation time. Control n=8, PB n=8. Wilcoxon-Mann-Whitney test.

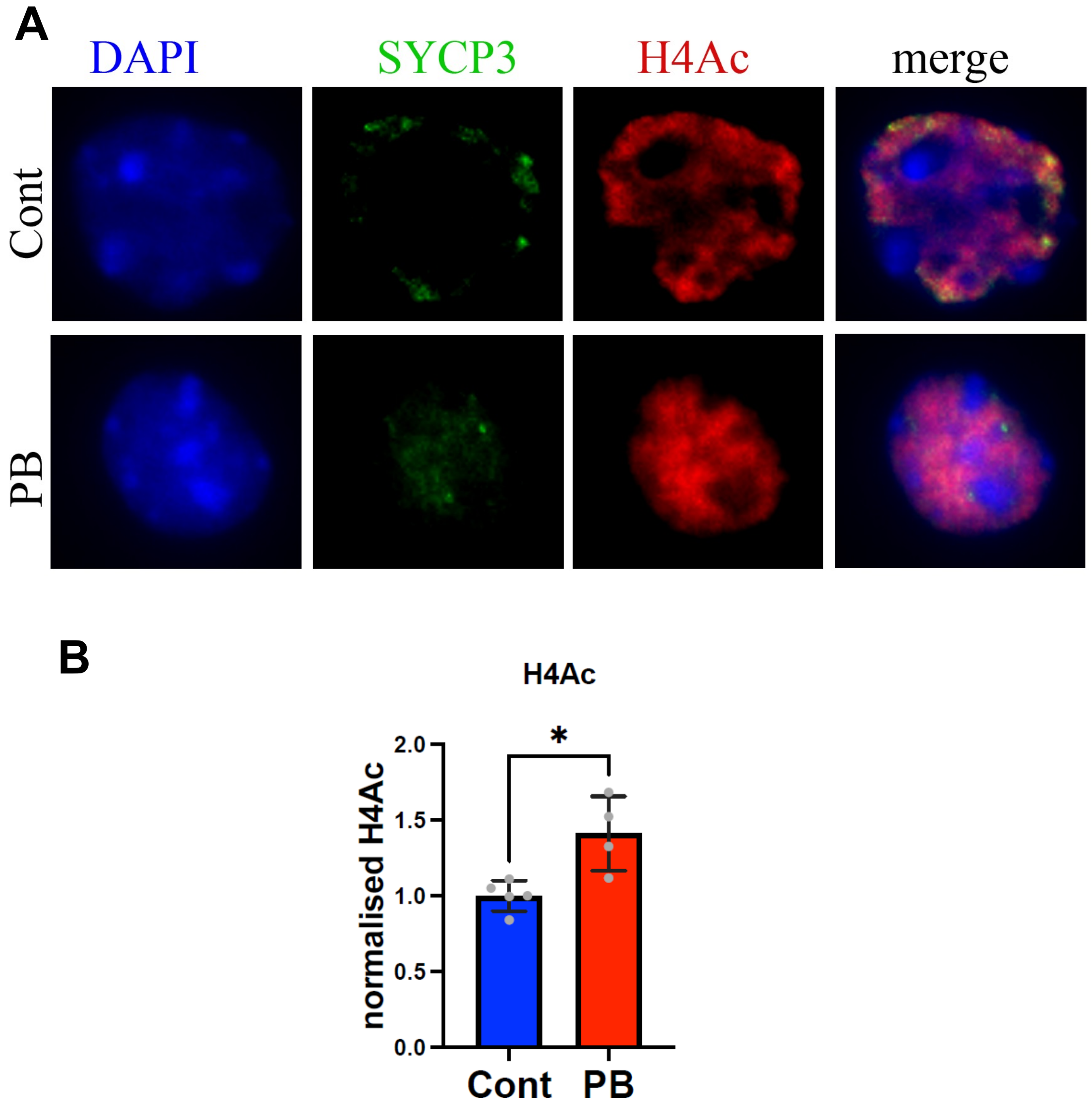

Additional file1: Fig.S5. Effects of PB exposure on H4acetylation.

(A) Structurally preserved nuclei from E15,5 ovaries from control (first row) and PB-exposed mice (second row) were immunostained with anti-H4Ac (red) and anti-SYCP3 (green) antibodies (63X magnification). (B) Quantitative analysis of immunofluorescence intensity. The data is presented as averaged fluorescence compared to control +/-SD, n=5 control, n=4, PB group, statistical significance was assessed by non-parametric Mann-Whitney test, p*<0.05.

A

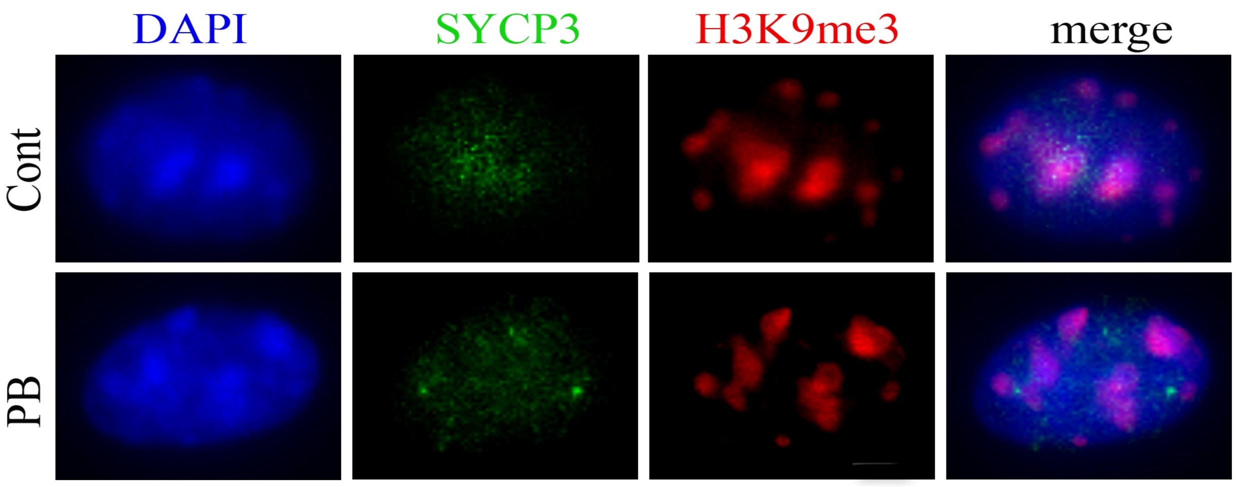

B

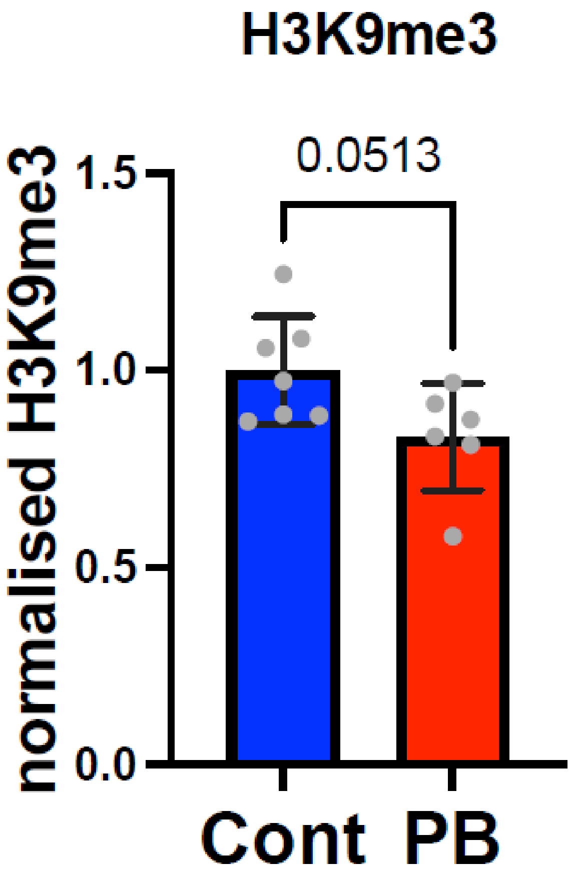

Additional file1: Fig.S6. Effects of PB exposure on H3K9me3.
(A) Structurally preserved nuclei from E15.5 ovaries from control (first row) and PB-exposed mice (second row) were immunostained with anti-H3K9me3 (red) and anti-SYCP3 (green) antibodies (63X magnification). (B) Quantitative analysis of immunofluorescence intensity. The data is presented as averaged fluorescence compared to control +/-SD, n=7 control, n=6, PB group, statistical significance was assessed by Mann-Whitney test, exact p-value is indicated on the top of column.

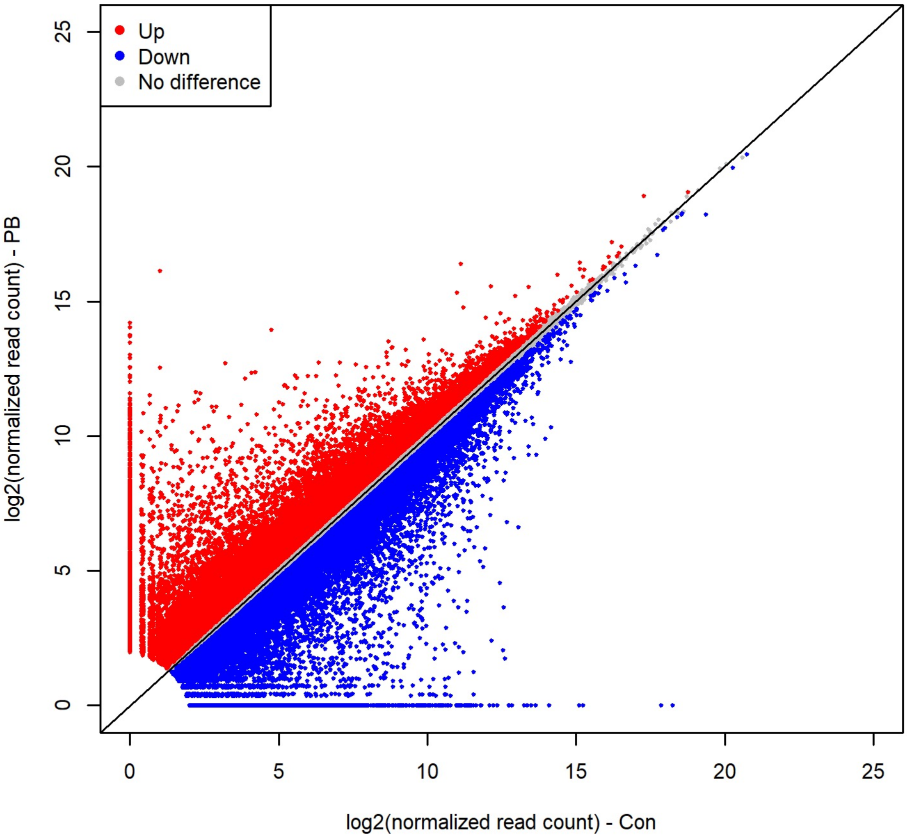

Additional file1: Fig.S7. Scatter plot of DEGs.

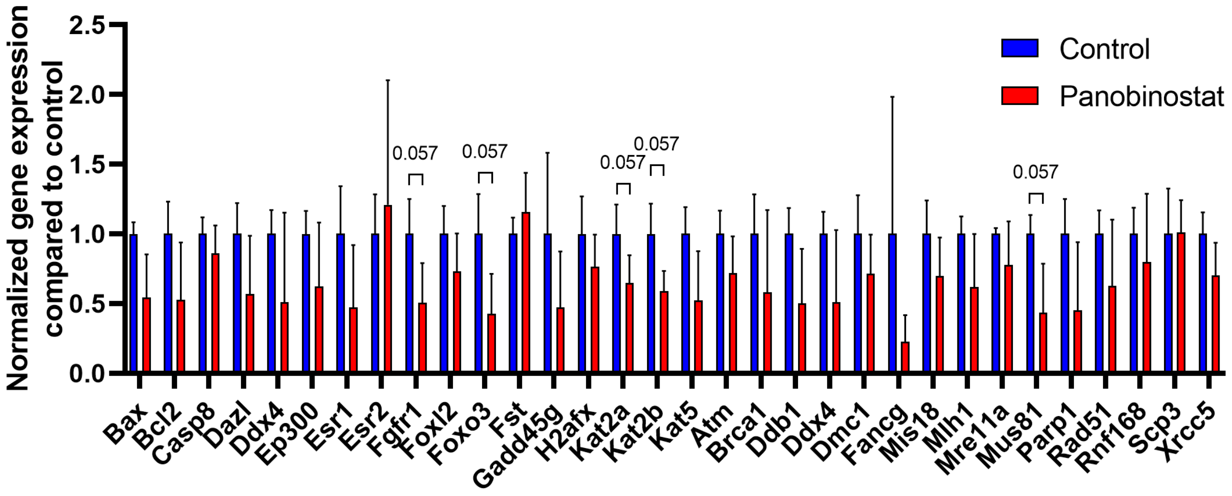

Additional file1: Fig.S8. Gene expression is altered in PND5 ovary.
Gene expression was normalized to the expression of *Rpl37a* housekeeping gene. For each group, n = 4. non-parametric Wilcoxon-Mann-Whitney test, exact p-values are indicated on the top of the columns.

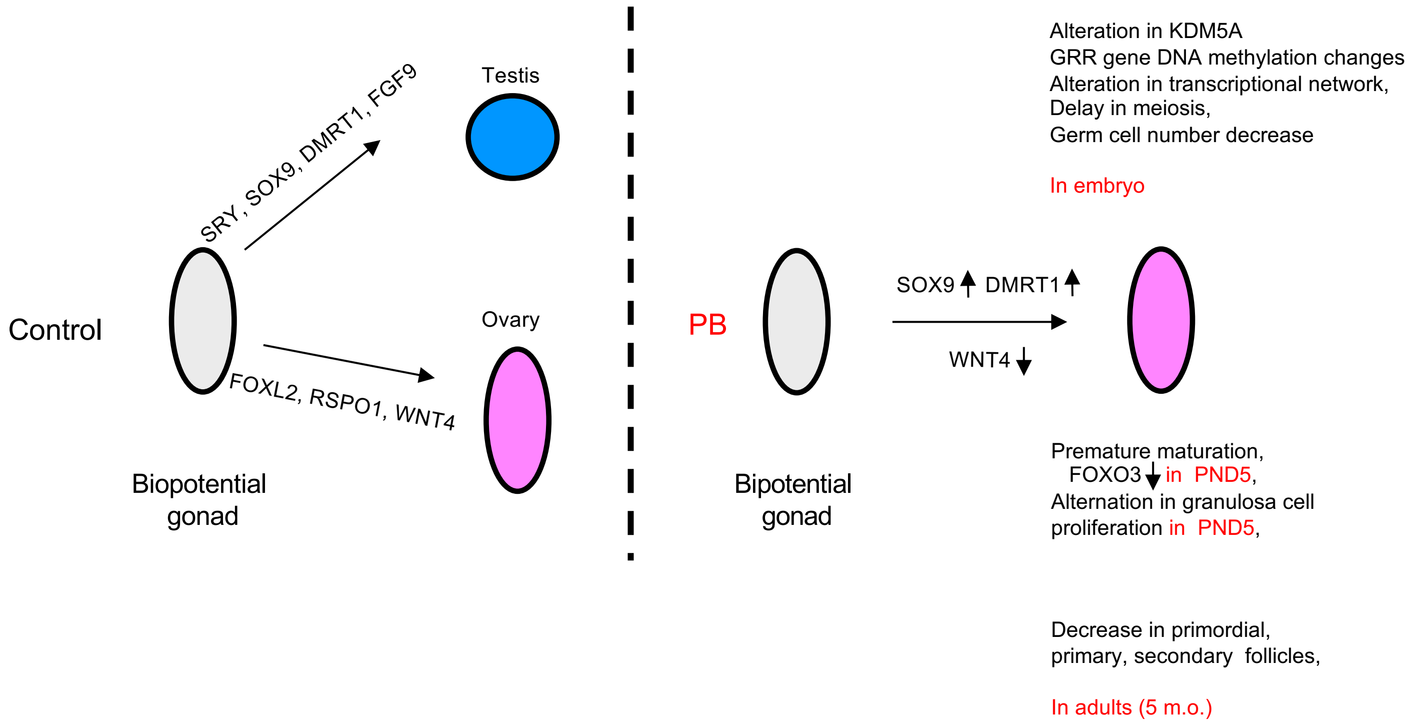

Additional file1: Fig.S9. Schematic presentation of the effects of PB on ovarian development.

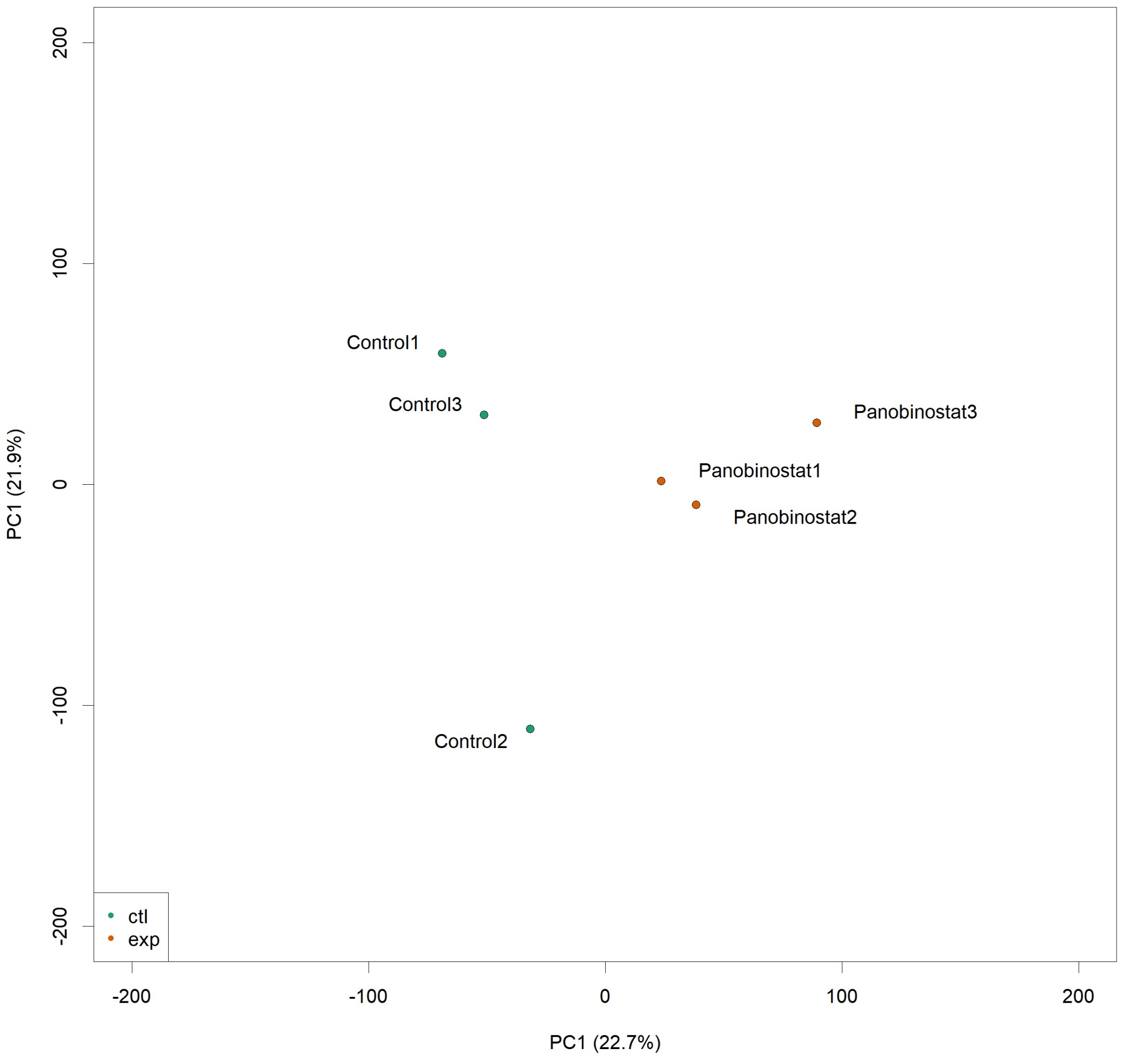

Additional file1: Fig.S10. Principal component analysis of RNA-seq data.

Additional file1: Table S1. Differentially expressed transcripts

Control1	Control2	Control3	PB1	PB2	PB3	log2FoldChange	P.adj	Strand	Gene Name	Transcript ID	Class Code
402,55	375,91	356,23	253,82	150,94	130,91	-1,08	9,45E-02	-	*1110002L01Rik*	NR_030694.1	=
7,36	17,73	11,98	0,00	0,00	0,00	-6,11	2,31E-03	-	*1700006A11Rik*	NM_027939.1	=
0,00	1,77	0,00	20,81	15,99	11,58	4,61	8,97E-03	-	*1700006A11Rik*	XM_006502121.1	=
2,10	0,00	2,18	17,68	10,00	37,40	3,98	2,68E-02	-	*1700019A02Rik*	NM_027070.1	=
3,15	8,87	6,54	0,00	0,00	0,00	-5,11	5,86E-02	-	*1700020M21Rik*	NR_040742.1	=
31,53	36,35	40,31	140,44	237,91	65,90	2,04	3,90E-02	-	*1700030J22Rik*	NM_027103.2	=
6,31	3,55	10,89	0,00	0,00	0,00	-5,26	4,95E-02	-	*1700031F05Rik*	XM_006528312.2	=
0,00	0,00	0,00	3,12	17,99	23,15	6,32	3,24E-02	+	*1700113H08Rik*	XM_006514284.2	=
43,09	39,90	29,41	0,00	0,00	1,78	-5,86	2,57E-05	+	*1700125H20Rik*	NM_028589.1	=
0,00	0,00	0,00	9,36	9,00	6,23	5,47	6,18E-03	+	*1700125H20Rik*	XM_006534340.3	=
8,41	3,55	37,04	0,00	0,00	0,00	-6,51	5,91E-02	+	*1810010H24Rik*	NM_001163473.1	=
14,71	1,77	22,88	0,00	0,00	0,00	-6,19	8,74E-02	+	*1810010H24Rik*	XM_017314737.1	=
10,51	26,60	51,20	3,12	3,00	4,45	-3,05	2,68E-02	+	*1810043G02Rik*	XM_006514008.1	=
28,38	57,63	23,97	210,13	114,96	160,30	2,14	4,43E-03	+	*1810043G02Rik*	XM_006514009.1	=
47,30	49,65	61,01	19,77	19,99	28,50	-1,20	1,84E-02	-	*2210011C24Rik*	NM_001291292.1	=
132,43	73,59	77,35	179,97	205,92	199,48	1,05	8,87E-02	-	*2610318N02Rik*	XM_006522537.2	=
34,68	96,64	3,27	0,00	0,00	0,00	-7,97	1,85E-02	-	*4430402I18Rik*	NM_198651.2	=
104,05	62,95	15,25	0,00	0,00	0,00	-8,40	1,69E-04	+	*4833420G17Rik*	XM_006517750.1	=
2576,12	2448,71	3695,17	1554,16	1397,48	1244,08	-1,06	5,82E-03	-	*4833439L19Rik*	NM_133797.4	=
0,00	0,00	0,00	6,24	4,00	7,12	4,97	3,84E-02	+	*4930433I11Rik*	NM_207248.3	=
6,31	7,09	8,72	0,00	0,00	0,00	-5,36	8,57E-03	+	*4930455B14Rik*	NR_045968.1	=
0,00	0,00	0,00	5,20	6,00	8,01	5,12	2,08E-02	+	*4930458A03Rik*	XR_880514.1	=
0,00	0,00	0,00	10,40	3,00	6,23	5,14	7,43E-02	-	*4930511M06Rik*	NR_027945.1	=
0,00	0,00	0,00	452,52	379,86	325,05	11,03	2,65E-16	+	*4932438A13Rik*	XM_006535456.3	=
65,17	49,65	4,36	5,20	1,00	0,89	-4,09	8,65E-02	+	*4933412E12Rik*	NR_038025.1	=
220,72	0,89	287,60	1,04	1,00	0,00	-7,99	8,64E-03	-	*4933427D14Rik*	XM_006534369.3	=
4,20	46,99	47,93	0,00	3,00	1,78	-4,37	7,58E-02	-	*4933427D14Rik*	XR_388544.3	=
0,00	0,00	0,00	4,16	6,00	6,23	4,89	4,01E-02	-	*4933428G20Rik*	NM_001289118.1	=
160,81	39,01	43,58	4,16	6,00	2,67	-4,25	3,39E-04	-	*5930430L01Rik*	NR_102383.1	=
63,06	152,49	2,18	1,04	4,00	0,89	-5,20	4,31E-02	+	*6330416G13Rik*	XM_006537867.2	=
213,36	41,67	100,22	10,40	21,99	17,81	-2,82	2,14E-02	+	*9030025P20Rik*	XM_011246223.2	=
0,00	0,00	0,00	208,05	5,00	182,56	9,48	2,71E-03	+	*9030025P20Rik*	XM_017317188.1	=
6,31	7,09	6,54	0,00	0,00	0,00	-5,21	1,26E-02	+	*9230105E05Rik*	NR_040626.1	=
5,26	5,32	5,45	0,00	0,00	0,00	-4,90	3,44E-02	+	*9530077C05Rik*	XM_006510570.3	=
42,04	9,75	49,02	0,00	0,00	0,00	-7,55	7,04E-04	+	*AA467197*	XM_006499873.3	=
1,05	0,89	1,09	177,89	2,00	266,27	7,21	2,46E-03	-	*Aamp*	XM_006495957.3	=
56,76	46,10	64,27	27,05	30,99	23,15	-1,04	4,52E-02	+	*Abca13*	NM_178259.3	=
72,52	91,32	2,18	3,12	3,00	2,67	-4,24	6,57E-02	-	*Abcc10*	XM_017317442.1	=
15,77	110,82	14,16	1,04	1,00	0,89	-5,59	4,80E-03	-	*Abcc5*	XM_006522214.1	=
0,00	0,00	0,00	297,52	211,92	320,59	10,55	2,19E-14	-	*Abcc5*	XM_006522209.3	=
0,00	0,00	0,00	1032,98	1038,61	1539,74	12,67	6,86E-21	-	*Abcc5*	XM_017317029.1	=
547,60	434,42	614,41	257,99	223,92	177,22	-1,28	3,43E-04	-	*Abhd17a*	NM_145421.2	=
0,00	0,00	0,00	298,56	131,95	349,09	10,46	4,88E-11	+	*Abi3bp*	XM_006522280.2	=
1277,03	939,77	0,00	0,00	0,00	0,00	-12,01	8,71E-02	+	*Abl2*	XM_006496620.3	=
437,24	414,92	7,63	3,12	15,99	2,67	-5,30	2,26E-02	+	*Abl2*	XM_006496613.3	=
1853,00	2555,10	2041,50	64,50	481,82	366,01	-2,82	3,02E-02	-	*Abtb1*	NM_030251.3	=
77,78	109,94	76,26	174,76	337,87	245,79	1,52	8,57E-03	-	*Abtb1*	XM_006506793.3	=
40,99	53,19	56,65	564,86	358,87	347,31	3,07	2,54E-15	-	*Abtb1*	XM_006506794.3	=
43,09	0,00	4,36	718,82	684,74	607,35	5,41	3,96E-02	-	*Abtb1*	XM_006506791.3	=
1,05	0,89	1,09	93,62	40,98	31,17	5,78	1,69E-05	-	*Abtb1*	XM_006506795.2	=
1815,16	715,46	907,45	0,00	0,00	0,00	-12,64	2,20E-16	+	*Acaca*	XM_011248667.1	=
12,61	7,09	14,16	1,04	0,00	0,00	-5,01	1,59E-02	-	*Acd*	XM_006531169.2	=
659,01	15,07	347,51	15,60	18,99	16,03	-4,33	2,56E-02	-	*Acot8*	XR_001781109.1	=
83,03	35,46	128,55	28,09	11,00	11,58	-2,29	8,58E-02	-	*Acot8*	XM_011239319.2	=
18,92	310,30	288,69	2,08	0,00	0,89	-7,71	7,84E-05	+	*Acsl1*	NM_001302163.1	=
780,93	862,64	1208,12	1570,80	1960,27	2431,16	1,06	4,61E-02	+	*Acsl1*	XM_006509264.3	=
112,46	86,00	14,16	3,12	0,00	1,78	-5,44	2,30E-03	+	*Actr5*	XM_006498566.1	=
523,42	496,48	786,53	134,19	277,90	132,69	-1,73	1,89E-02	-	*Adam15*	NM_001037722.3	=
5,26	0,00	0,00	50,97	20,99	98,85	5,04	8,41E-02	-	*Adam2*	NM_009618.3	=
1198,20	1490,33	1648,23	0,00	0,00	0,00	-12,98	4,94E-23	-	*Adam22*	NM_001098225.2	=
9,46	113,48	133,99	0,00	0,00	0,89	-7,94	1,04E-03	-	*Adam22*	NM_001007221.3	=
44,14	34,58	81,70	2,08	0,00	0,00	-6,34	6,44E-05	-	*Adam22*	XM_006503540.3	=
0,00	0,00	0,00	4,16	46,98	88,16	7,97	1,02E-02	-	*Adam33*	XR_001780408.1	=
3,15	2,66	1,09	1,04	629,76	390,05	7,20	3,96E-03	+	*Adamts10*	XM_017317417.1	=
0,00	0,00	0,00	0,00	1200,55	1686,68	12,35	7,12E-02	+	*Adamts10*	XM_017317412.1	=
6,31	9,75	10,89	1477,18	11,00	82,82	5,86	1,70E-02	+	*Adamts17*	XM_006540843.3	=
6,31	5,32	9,80	89,46	217,92	5,34	3,87	9,11E-02	+	*Adamts19*	XM_006525922.3	=
0,00	0,00	0,00	512,85	1333,50	4919,32	13,57	1,95E-10	-	*Adamts9*	XM_006505263.3	=
62,01	10,64	1014,21	0,00	0,00	0,00	-10,98	2,60E-03	+	*Adck4*	XM_006540422.2	=
1,05	0,89	0,00	324,56	3,00	402,52	8,54	1,27E-03	-	*Adcy9*	XM_011245805.1	=
0,00	0,00	0,00	85,30	37,99	75,70	8,49	1,26E-07	+	*Adcyap1*	NM_009625.3	=
216,52	121,46	84,97	2,08	2,00	6,23	-5,33	2,14E-09	+	*Adgrf5*	XM_006524125.3	=
0,00	0,00	0,00	67,62	71,97	8,01	8,06	1,65E-03	+	*Adgrg3*	XM_006531199.3	=
653,75	1031,97	1,09	0,00	1,00	0,89	-9,79	8,74E-04	+	*Adgrl1*	XM_011248415.1	=
81,98	78,02	116,56	218,46	222,92	272,50	1,37	7,70E-05	+	*Agbl5*	NM_174849.3	=
9,46	4,43	22,88	63,46	68,97	82,82	2,56	1,23E-02	+	*Agbl5*	XM_006503871.1	=
832,43	130,33	1177,62	67,62	9,00	94,40	-3,65	7,22E-02	+	*Agpat1*	XM_006524647.2	=
2,10	117,91	74,08	0,00	0,00	0,00	-8,50	1,82E-02	-	*Agtpbp1*	XM_011244556.2	=
2,10	98,41	72,99	0,00	0,00	0,00	-8,33	1,93E-02	-	*Agtpbp1*	XM_006517339.2	=
14,71	76,25	40,31	899,83	201,92	122,00	3,22	9,22E-02	-	*Ahsa2*	XM_006514710.2	=
2,10	0,00	2,18	12,48	7,00	12,47	2,97	8,65E-02	+	*Aifm3*	XM_006522595.3	=
0,00	374,13	1788,76	0,00	0,00	0,00	-11,97	8,89E-02	+	*Ak4*	NM_001177605.1	=
93,54	366,15	29,41	0,00	0,00	0,00	-9,83	8,57E-05	-	*Akap7*	XM_006512787.3	=
0,00	0,00	0,00	8,32	8,00	17,81	5,95	4,84E-03	+	*Akr1d1*	XM_006505827.2	=
1,05	6,21	9,80	1443,89	1,00	1397,25	7,38	7,51E-03	-	*Akr1e1*	XM_006516485.1	=

Additional file1: Table S1. Differentially expressed transcripts (*continued*)

Control1	Control2	Control3	PB1	PB2	PB3	log2FoldChange	P.adj	Strand	Gene Name	Transcript ID	Class Code
1474,62	1262,48	1361,72	509,73	891,67	625,16	-1,02	3,91E-02	+	*Akt1s1*	NM_001290694.1	=
2716,97	2523,19	2512,11	5198,21	5909,79	5083,18	1,06	5,82E-17	+	*Akt1s1*	NM_026270.4	=
13,66	14,19	34,86	101,95	54,98	126,46	2,18	2,98E-02	+	*Aldoc*	NM_009657.4	=
0,00	0,00	0,00	0,00	2219,17	844,23	12,43	6,73E-02	+	*Alms1*	NM_145223.2	=
8,41	2,66	4,36	201,81	272,90	5,34	4,96	1,91E-02	-	*Alx1*	XM_006513529.2	=
462,46	382,11	379,10	1326,34	1225,54	1043,71	1,56	1,63E-13	+	*Amhr2*	XM_006520294.3	=
0,00	0,00	0,00	136,27	350,87	112,21	10,08	6,47E-09	+	*Amhr2*	XM_006520293.2	=
5,26	4,43	10,89	31,21	29,99	49,87	2,45	2,08E-03	+	*Ammecr1l*	XM_006525844.2	=
69,37	79,79	74,08	78,02	743,72	868,27	2,92	7,57E-02	+	*Ammecr1l*	XM_006525843.3	=
16,82	13,30	17,43	8,32	7426,23	8263,29	8,37	1,08E-04	-	*Anapc1*	XM_011239333.2	=
33,63	31,03	18,52	9,36	11,00	7,12	-1,61	5,12E-02	+	*Ank1*	NR_102386.2	=
18,92	58,51	37,04	41,61	6964,40	3644,08	6,54	1,08E-03	+	*Ank1*	XM_006508995.3	=
125,08	116,14	123,10	14,56	2,00	5,34	-4,07	1,85E-06	+	*Ankra2*	XM_017315580.1	=
70,42	85,11	78,44	17,68	14,99	24,94	-2,01	1,69E-07	+	*Ankra2*	NM_001271389.1	=
3,15	1,77	3,27	31,21	128,95	233,32	5,60	8,77E-06	-	*Ankrd42*	XM_017312337.1	=
0,00	0,00	0,00	17,68	22,99	24,04	6,87	1,23E-05	+	*Ankrd7*	XM_006505192.3	=
0,00	0,00	0,00	0,00	8153,95	10063,06	28,11	2,49E-10	-	*Ano1*	NM_178642.5	=
0,00	0,00	0,00	512,85	1,00	1915,54	12,10	4,10E-03	-	*Anxa4*	XM_006505397.2	=
1,05	16033,67	13271,90	0,00	0,00	0,00	-15,73	1,55E-04	+	*Ap2m1*	NM_009679.3	=
987,99	31,03	248,38	0,00	0,00	0,00	-11,20	5,42E-05	+	*Ap4e1*	XM_006498551.3	=
6570,12	2,66	7108,20	2,08	9,00	1,78	-10,06	3,23E-05	+	*Apc*	XM_006525535.1	=
32,58	513,33	1,09	0,00	0,00	0,00	-9,99	3,31E-02	-	*Appl1*	XM_006519608.1	=
36,79	13,30	53,38	0,00	0,00	0,00	-7,59	1,70E-04	+	*Aqp12*	NM_001159658.1	=
2,10	11,53	122,01	0,00	0,00	0,00	-7,98	6,69E-02	+	*Arap1*	XM_011241897.2	=
1845,64	909,62	836,64	186,21	41,98	222,63	-2,99	1,14E-02	+	*Arap1*	XM_006508196.3	=
1,05	1,77	1,09	2,08	76,97	1405,27	8,56	1,49E-03	+	*Arhgap23*	XM_006533868.3	=
5,26	4,43	2,18	24,97	19,99	32,95	2,71	7,52E-04	-	*Arhgap33*	XM_011250546.2	=
3,15	5,32	5,45	60,34	43,98	10,69	3,04	3,30E-02	-	*Arhgap4*	XM_011247531.2	=
0,00	0,00	0,00	3170,73	0,00	2392,87	13,29	3,73E-02	-	*Arhgap42*	NM_027823.1	=
0,00	2,66	0,00	1441,81	0,00	2020,63	10,32	6,15E-02	+	*Arhgap5*	XM_011243990.1	=
64,11	21,28	4,36	0,00	1,00	0,00	-6,42	2,44E-02	+	*Arhgap8*	XM_006521468.3	=
554,95	742,95	614,41	0,00	0,00	0,00	-11,80	5,53E-19	-	*Arhgef15*	XM_017314643.1	=
365,77	619,71	21,79	8,32	8,00	11,58	-5,17	1,23E-03	+	*Arhgef2*	XM_006501067.3	=
13,66	5,32	0,00	628,32	577,78	501,37	6,49	4,29E-07	+	*Arhgef2*	XM_006501066.1	=
0,00	0,00	0,00	116,51	94,96	160,30	9,39	7,93E-11	+	*Arhgef2*	NM_001198911.1	=
0,00	0,89	0,00	0,00	245,91	194,14	8,67	9,79E-02	-	*Arhgef25*	XM_006513880.1	=
908,11	109,05	13,07	6,24	13,99	7,12	-5,24	1,96E-02	+	*Arid3a*	XM_006513201.3	=
8,41	15,96	9,80	31,21	26,99	52,54	1,69	7,17E-02	+	*Armc12*	NM_026290.3	=
158,71	319,17	1,09	1,04	1,00	0,00	-7,90	8,72E-03	+	*Armc9*	NM_001310702.1	=
0,00	0,00	0,00	6,24	85,97	115,77	8,55	2,90E-03	+	*Armcx1*	XM_006528624.2	=
0,00	0,00	0,00	186,21	74,97	95,29	9,33	8,15E-09	+	*Armcx1*	NM_001166380.1	=
0,00	130,33	331,17	0,00	1,00	0,00	-8,78	9,77E-02	+	*Arpc4*	NM_001170485.1	=
21,02	68,27	74,08	0,00	0,00	0,00	-8,25	1,26E-05	-	*Arpp21*	XM_006512326.3	=
0,00	0,00	0,00	18,72	249,91	50,76	9,17	7,25E-04	-	*Arpp21*	XM_017313650.1	=
2,10	296,12	34,86	0,00	4,00	1,78	-5,85	9,09E-02	+	*Arrb2*	XM_006532917.1	=
188,14	831,61	323,55	0,00	3,00	20,48	-5,83	8,98E-03	-	*Arrdc2*	XR_379046.3	=
88,29	2,66	64,27	3,12	2,00	0,00	-4,94	8,78E-02	+	*Art1*	XR_869649.1	=
6,31	3,55	40,31	0,00	0,00	0,00	-6,54	8,03E-02	-	*Artn*	XM_006502692.3	=
156,61	80,68	157,96	2,08	4,00	2,67	-5,50	5,09E-13	+	*Asap3*	XM_006538762.3	=
21,02	67,38	11,98	1310,73	154,94	995,62	4,61	1,76E-03	-	*Ash2l*	NM_001286207.1	=
403,60	358,18	650,36	1618,65	1818,32	1774,84	1,88	1,73E-06	+	*Asxl2*	XM_006515242.2	=
3388,59	522,19	348,60	0,00	0,00	0,00	-12,95	1,71E-08	+	*Atad2b*	XM_017315117.1	=
96,70	485,84	42,49	15,60	10,00	19,59	-3,79	2,14E-02	+	*Atad2b*	XM_017315116.1	=
0,00	0,00	0,00	39,53	38,99	84,60	8,20	9,26E-07	+	*Athl1*	XM_017322105.1	=
1,05	0,89	0,00	0,00	116,96	262,71	7,60	6,95E-02	-	*Atl2*	XM_006524654.3	=
2196,70	1578,99	3216,94	7,28	22,99	257,37	-4,60	3,02E-02	-	*Atp11c*	XM_011247616.1	=
254,35	481,41	239,66	3060,46	3725,61	3853,35	3,45	6,06E-15	-	*Atp11c*	NM_001037863.1	=
881,83	4061,39	4606,98	917,51	370,86	382,04	-2,52	9,08E-02	+	*Atp13a1*	XM_006509564.3	=
2,10	1,77	5,45	392,18	121,95	0,89	5,79	3,90E-02	+	*Atp2a3*	NM_001163337.1	=
10,51	2,66	8,72	0,00	0,00	0,00	-5,34	6,94E-02	+	*Atp6v0a1*	XM_006532050.2	=
0,00	0,00	0,00	314,16	273,90	309,91	10,66	6,88E-16	+	*Atp6v0a1*	XM_006532053.1	=
0,00	0,00	0,00	357,85	270,90	293,88	10,70	1,66E-15	+	*Atp6v0a1*	NM_016920.3	=
1,05	0,89	1,09	16,64	21,99	14,25	4,13	1,57E-03	-	*Atxn2l*	XM_017322209.1	=
55,71	0,89	45,75	0,00	1,00	0,89	-5,74	7,75E-02	+	*Axin2*	XM_006532060.1	=
26,28	48,76	94,78	1,04	0,00	0,89	-6,45	8,11E-05	+	*B3galnt2*	XM_006516818.3	=
7,36	3,55	6,54	0,00	0,00	0,00	-5,01	4,62E-02	-	*B3gntl1*	XM_017314416.1	=
14,71	8,87	18,52	141,48	82,97	105,97	2,99	2,70E-08	-	*B4galt2*	NM_001253381.1	=
1241,29	21,28	1324,68	40,57	14,99	2,67	-5,48	2,30E-02	+	*B4galt4*	XR_384580.3	=
64,11	67,38	47,93	61,38	1430,47	1076,66	3,84	2,92E-02	+	*Bach2*	NM_001109661.1	=
0,00	6,21	1,09	55,13	148,94	135,36	5,49	2,99E-04	+	*Baz2a*	NM_054078.2	=
9,46	3,55	0,00	179,97	124,95	267,16	5,46	2,87E-05	-	*Baz2b*	XM_017319088.1	=
0,00	0,00	3,27	52,01	13,99	82,82	5,56	2,14E-02	-	*Baz2b*	XM_017319083.1	=
0,00	0,00	0,00	82,18	21,99	94,40	8,48	1,69E-05	-	*Baz2b*	XM_017319100.1	=
3,15	6,21	5,45	11,44	108,96	95,29	3,86	1,21E-02	+	*Bbs12*	XM_006535480.1	=
0,00	401,62	522,90	2,08	0,00	2,67	-7,60	7,43E-02	+	*Bcas3*	XM_006532540.2	=
756,76	468,11	726,62	2367,64	3136,83	2495,28	2,04	6,28E-09	+	*Bcas3*	NM_001166643.1	=
2785,28	3527,67	2427,14	1506,30	1491,44	1353,62	-1,01	4,34E-04	-	*Bcl2l1*	XM_006498609.3	=
178,68	126,78	166,67	1,04	0,00	0,00	-8,81	3,13E-10	-	*Bcor*	XM_017318624.1	=
274,32	427,33	3,27	0,00	0,00	1,78	-8,58	6,31E-03	+	*Bend3*	XM_006512769.3	=
15,77	11,53	22,88	112,35	136,95	27,61	2,47	6,69E-02	+	*Bicd2*	XM_011244429.2	=
27,33	22,16	23,97	79,06	65,98	85,49	1,66	2,13E-06	+	*Bin1*	XM_006525993.3	=
9,46	0,00	0,00	155,00	193,93	320,59	6,15	2,39E-02	+	*Birc6*	XM_006523524.2	=
8,41	0,00	0,00	157,08	193,93	366,90	6,43	1,15E-02	+	*Birc6*	XM_006523520.2	=
57,81	109,05	135,08	454,60	237,91	416,77	1,88	1,44E-02	+	*Bivm*	XM_006496004.3	=
186,04	208,34	279,97	1168,22	367,86	976,03	1,90	4,19E-02	+	*Bivm*	NM_144558.4	=

Additional file1: Table S1. Differentially expressed transcripts (*continued*)

Control1	Control2	Control3	PB1	PB2	PB3	log2FoldChange	P.adj	Strand	Gene Name	Transcript ID	Class Code
90,39	74,47	27,23	426,51	176,93	665,23	2,72	2,07E-02	-	*Bptf*	XM_006532691.1	=
1391,59	1824,57	0,00	0,00	1,00	0,89	-10,72	4,34E-03	-	*Brca1*	XM_006532064.3	=
3605,10	94,86	4463,18	18,72	116,96	74,81	-5,28	8,57E-03	-	*Brd3*	NM_001113574.1	=
9,46	7,09	4,36	16,64	41,98	75,70	2,68	4,01E-02	+	*Brd9*	XM_017315339.1	=
2,10	429,10	287,60	2,08	3,00	1,78	-6,71	2,80E-03	-	*Brox*	XM_006535598.3	=
72,52	18,62	393,27	8,32	0,00	5,34	-5,15	6,25E-02	-	*Btf3l4*	XM_011240610.1	=
0,00	0,00	0,00	4,16	126,95	85,49	8,61	6,42E-03	-	*Btk*	XM_006528483.3	=
407,81	666,70	638,38	230,94	245,91	333,06	-1,08	5,92E-02	+	*C530008M17Rik*	XM_006535010.3	=
15,77	5,32	14,16	220,54	268,90	19,59	3,86	2,43E-02	+	*Cacna1a*	XM_006530588.3	=
207,06	197,71	574,10	0,00	0,00	0,00	-10,83	2,13E-10	+	*Cacna2d2*	NM_001174049.1	=
21,02	2,66	8,72	406,74	318,88	375,81	5,10	7,65E-09	+	*Cacna2d2*	XM_017313496.1	=
0,00	0,00	0,00	7,28	21,99	6,23	6,00	1,98E-02	+	*Cacna2d2*	XM_017313497.1	=
25,23	31,03	19,61	66,58	79,97	97,96	1,68	1,76E-04	+	*Cacnb3*	NM_001044741.2	=
0,00	0,00	0,00	22,89	301,89	105,08	9,60	1,19E-04	-	*Camk2b*	XM_017314241.1	=
0,00	0,00	0,00	18,72	11,00	11,58	6,22	5,69E-04	+	*Camk2d*	NM_001293665.1	=
54,65	50,53	37,04	294,40	67,97	314,36	2,25	7,20E-02	-	*Camk2g*	XM_006518489.2	=
54,65	49,65	39,22	313,12	67,97	317,92	2,28	6,80E-02	-	*Camk2g*	XM_006518493.2	=
54,65	54,97	44,66	386,98	70,97	423,00	2,51	5,77E-02	-	*Camk2g*	XM_006518492.2	=
49,40	39,01	32,68	410,90	48,98	334,84	2,71	7,95E-02	-	*Camk2g*	XM_006518496.3	=
50,45	39,90	31,59	435,87	50,98	339,29	2,76	7,37E-02	-	*Camk2g*	XM_017315826.1	=
213,36	0,00	162,32	0,00	1,00	0,89	-7,62	6,83E-02	+	*Car12*	XM_006511548.3	=
51,50	49,65	47,93	23,93	17,99	30,28	-1,04	5,87E-02	+	*Casp1*	NM_009807.2	=
0,00	2080,79	1853,03	0,00	0,00	0,00	-12,84	5,12E-02	+	*Casp8ap2*	XM_006537949.1	=
47,30	51,42	3,27	2,08	1,00	0,00	-5,08	4,81E-02	-	*Cast*	NM_001301181.1	=
0,00	1,77	0,00	117,55	2,00	505,82	8,40	1,74E-02	-	*Cbarp*	NM_001195268.1	=
9,46	0,00	3,27	197,65	87,97	46,31	4,72	1,83E-02	-	*Cbs*	XM_006523549.3	=
573,87	2669,47	2745,23	444,19	393,85	463,08	-2,20	7,43E-02	-	*Cc2d1a*	XM_006530810.2	=
0,00	2,66	0,00	12,48	35,99	31,17	4,79	1,20E-02	-	*Ccdc116*	XM_006522709.3	=
1783,63	23,05	1473,93	61,38	32,99	76,59	-4,26	7,17E-02	-	*Ccdc64*	XR_387579.2	=
0,00	2,66	0,00	81,14	147,94	1,78	6,39	9,14E-02	-	*Ccdc77*	XM_011241435.2	=
1,05	0,00	0,00	21,85	56,98	16,03	6,46	1,54E-03	+	*Ccl25*	XM_006508739.3	=
8,41	0,00	1,09	128,99	129,95	105,97	5,28	1,19E-03	-	*Ccnb3*	XM_017318448.1	=
2,10	1,77	3,27	9,36	12,00	14,25	2,35	5,68E-02	-	*Ccny*	XM_017317974.1	=
1340,09	1197,76	11,98	10,40	8,00	13,36	-6,33	1,03E-03	-	*Ccz1*	XM_017320876.1	=
4,20	31,03	28,32	0,00	0,00	0,00	-6,89	1,23E-02	+	*Cd300a*	XM_006533070.3	=
690,54	82,45	578,46	37,45	68,97	27,61	-3,33	2,13E-02	-	*Cd36*	NM_007643.4	=
0,00	0,00	0,00	4,16	13,00	6,23	5,40	4,00E-02	-	*Cd46*	XR_373889.3	=
28,38	24,82	28,32	63,46	65,98	52,54	1,16	3,38E-03	+	*Cd47*	XM_006521807.3	=
0,00	0,00	0,00	3,12	27,99	8,01	6,14	7,37E-02	+	*Cd47*	XM_006521809.3	=
6,31	2,66	6,54	0,00	0,00	0,00	-4,84	9,51E-02	-	*Cd7*	NM_009854.2	=
0,00	0,00	0,00	21,85	23,99	11,58	6,69	1,63E-04	-	*Cdc14b*	XM_006517219.1	=
5081,83	114,37	3787,77	0,00	0,00	0,00	-14,03	1,07E-07	-	*Cdc27*	XM_006533051.2	=
31,53	19,50	26,15	67,62	61,98	65,90	1,35	8,49E-04	-	*Cdc27*	NM_145436.2	=
433,03	318,28	3,27	3,12	2,00	15,14	-5,21	5,66E-02	+	*Cdc6*	NM_011799.2	=
269,07	34,58	4,36	0,00	0,00	0,00	-9,16	1,31E-02	+	*Cdca7l*	XM_011244099.2	=
0,00	0,00	1,09	121,71	181,93	0,89	8,14	2,13E-02	-	*Cdcp1*	XM_006511922.3	=
4,20	4,43	0,00	33,29	15,99	15,14	2,87	9,05E-02	-	*Cdh22*	XM_017314853.1	=
17,87	13,30	17,43	0,00	0,00	0,00	-6,49	7,78E-05	+	*Cdh7*	XM_006529600.3	=
0,00	0,00	0,00	27,05	15,99	6,23	6,47	5,67E-03	+	*Cdh7*	XM_017320703.1	=
13,66	8,87	9,80	28,09	78,97	32,06	2,11	5,59E-02	+	*Cdk12*	XM_006534108.3	=
4363,96	0,00	3613,47	0,00	0,00	0,00	-13,86	2,46E-02	-	*Cdk13*	XM_006516767.3	=
4101,20	2,66	4871,70	4,16	0,00	0,00	-11,09	1,88E-03	-	*Cdk13*	XM_006516766.3	=
148,20	125,89	22,88	0,00	0,00	0,00	-9,11	2,29E-05	+	*Cdk17*	XM_011243447.2	=
10,51	71,81	56,65	0,00	0,00	0,00	-8,01	6,08E-04	-	*Cdpf1*	XM_006521448.1	=
404,65	3,55	1957,61	1,04	16,99	1,78	-6,90	1,93E-02	-	*Celf2*	NM_001110230.1	=
352,10	3,55	942,31	1,04	15,99	1,78	-6,11	3,75E-02	-	*Celf2*	NM_001110229.1	=
371,02	0,89	340,98	1,04	11,00	0,89	-5,79	8,51E-02	-	*Celf2*	XM_017315442.1	=
324,77	20,39	427,04	5,20	13,99	5,34	-4,98	2,76E-03	-	*Celf2*	XM_006497339.2	=
360,51	52,31	837,73	13,52	34,99	64,12	-3,47	9,08E-02	-	*Celf2*	NM_001160292.1	=
0,00	0,00	0,00	167,48	560,79	569,94	11,19	6,53E-11	-	*Cenpc1*	XM_006534739.3	=
142,94	158,70	43,58	19,77	16,99	8,01	-2,95	4,61E-03	+	*Cep135*	XR_389278.3	=
725,22	190,61	620,95	2279,22	1413,47	2082,96	1,91	9,94E-02	+	*Cep250*	XM_006498797.3	=
80,93	80,68	99,13	0,00	0,00	0,00	-8,92	1,30E-10	-	*Cep41*	XM_006505207.3	=
74,62	51,42	94,78	0,00	0,00	0,00	-8,68	6,78E-09	-	*Cep41*	XM_006505210.3	=
4,20	54,08	61,01	0,00	1,00	1,78	-5,39	2,19E-02	+	*Cfap126*	XM_006497018.2	=
0,00	0,00	0,00	11,44	18,99	41,86	7,03	8,64E-04	-	*Cfap206*	NM_027041.4	=
0,00	0,00	0,00	33,29	49,98	43,64	7,84	1,32E-07	-	*Cfap206*	XM_006538232.3	=
198,65	80,68	430,30	4738,41	1197,55	4001,18	3,81	1,62E-03	+	*Cflar*	NM_207653.5	=
45,20	30,14	30,50	35,37	1305,51	1391,02	4,69	1,04E-02	-	*Chd3*	XM_006532881.1	=
5,26	1,77	3,27	79,06	5,00	138,92	4,45	3,11E-02	+	*Chek2*	XM_017320973.1	=
1553,45	1754,53	186,28	124,83	57,98	61,45	-3,84	4,55E-03	-	*Chid1*	NM_026522.5	=
43,09	27,48	16,34	0,00	0,00	0,00	-7,34	4,05E-05	+	*Chka*	XM_006531651.3	=
19,97	2,66	35,95	0,00	0,00	0,00	-6,76	3,83E-02	+	*Chrd*	XM_011245816.2	=
92,49	104,62	116,56	0,00	0,00	0,00	-9,19	2,52E-11	-	*Clcn2*	XM_006521749.3	=
1,05	0,00	0,00	18,72	433,84	391,84	9,61	5,73E-05	-	*Clcn5*	XM_011247443.2	=
10,51	28,37	103,49	0,00	2,00	0,00	-6,17	1,98E-02	+	*Cldn34c2*	XM_006528427.2	=
0,00	2,66	3,27	13,52	9,00	14,25	2,62	7,75E-02	+	*Clec16a*	XM_006522662.2	=
0,00	0,00	0,00	163,32	11,00	1,78	8,31	7,17E-02	+	*Clgn*	XM_006530636.2	=
0,00	2066,60	879,13	0,00	0,00	0,00	-12,42	6,79E-02	-	*Clip1*	XM_006530411.3	=
0,00	1706,65	428,13	0,00	0,00	0,00	-11,95	8,99E-02	-	*Clip1*	XM_006530421.3	=
0,00	0,00	0,00	34,33	20,99	21,37	7,11	1,09E-05	+	*Clk4*	XM_017314244.1	=
174,47	66,49	47,93	1681,07	894,67	1038,37	3,65	9,77E-06	-	*Clptm1*	XM_006540227.1	=
74,62	65,61	100,22	0,00	0,00	0,00	-8,80	8,84E-10	-	*Cnpy3*	NM_001305989.1	=
1,05	0,89	1,09	68,66	64,98	0,89	5,48	4,49E-02	+	*Cnr1*	XM_017319939.1	=
87,24	148,94	196,09	5,20	0,00	0,00	-6,41	1,06E-03	+	*Cntrl*	XM_006498074.3	=

Additional file1: Table S1. Differentially expressed transcripts (*continued*)

Control1	Control2	Control3	PB1	PB2	PB3	log2FoldChange	P.adj	Strand	Gene Name	Transcript ID	Class Code
111,41	92,20	9,80	4,16	11,00	6,23	-3,32	7,11E-02	+	*Cog5*	XM_011243869.2	=
0,00	0,00	0,00	9,36	27,99	19,59	6,68	7,74E-04	-	*Col13a1*	NM_007731.3	=
24,17	48,76	1,09	1,04	1,00	0,89	-4,66	9,80E-02	+	*Col25a1*	XM_006502266.3	=
509,76	603,76	220,05	42,65	141,95	69,46	-2,39	3,24E-02	+	*Cops4*	NM_012001.2	=
2,10	67,38	205,89	0,00	1,00	0,00	-8,04	1,36E-02	-	*Coro1a*	XM_006507285.3	=
6,31	54,97	0,00	756,27	453,83	945,75	5,13	6,72E-02	-	*Cpeb1*	XM_011250785.2	=
6,31	7,09	0,00	526,37	187,93	512,06	6,50	1,35E-07	-	*Cpeb1*	XM_011250784.2	=
0,00	4,43	19,61	122,75	510,81	168,31	5,06	2,83E-02	-	*Cpeb3*	XM_011247207.1	=
90,39	82,45	93,69	27,05	22,99	51,65	-1,38	5,91E-02	-	*Crb1*	XM_006529224.3	=
3228,83	4332,68	0,00	0,00	0,00	0,00	-13,78	2,60E-02	-	*Crebbp*	XM_006521753.3	=
15,77	8,87	0,00	9799,30	10201,19	0,00	9,66	3,93E-02	-	*Crebbp*	XM_006521754.3	=
32,58	32,80	71,90	0,00	0,00	0,00	-7,99	2,69E-06	+	*Crim1*	XM_006524603.3	=
322,67	346,65	413,96	640,80	767,71	1226,27	1,28	2,52E-02	-	*Csad*	XM_011245638.1	=
7,36	9,75	22,88	469,16	9,00	831,76	5,04	2,37E-02	-	*Csgalnact1*	XM_006509626.1	=
14481,37	14157,68	13445,11	4496,03	4636,27	4078,66	-1,67	2,37E-61	+	*Csnk1a1*	XM_006526388.2	=
146,10	152,49	192,82	66,58	52,98	51,65	-1,52	5,50E-07	+	*Csnk1a1*	XM_006526385.2	=
1897,15	1820,13	1752,81	646,00	605,77	684,82	-1,50	5,84E-45	+	*Csnk1a1*	XM_006526386.3	=
13675,22	13845,61	12966,88	5474,92	4887,17	4658,40	-1,43	2,16E-34	+	*Csnk1a1*	XM_006526384.2	=
217,57	327,15	273,43	142,52	106,96	87,27	-1,28	7,81E-03	+	*Csnk1a1*	XM_006526383.3	=
373,12	294,34	510,92	7570,01	7567,17	8452,97	4,32	5,00E-47	+	*Csnk1a1*	XR_386022.3	=
0,00	0,00	0,00	11035,14	10931,92	11788,92	15,89	4,81E-37	+	*Csnk1a1*	XR_386023.3	=
56,76	29,26	91,51	0,00	0,00	0,00	-8,36	2,22E-06	-	*Csnk1e*	XM_011245650.2	=
2,10	0,89	2,18	310,00	411,85	13,36	7,17	5,35E-05	-	*Csnk1e*	NM_001289899.1	=
3,15	186,18	716,81	3,12	2,00	0,00	-7,48	7,97E-03	+	*Csrnp3*	XM_006500402.2	=
0,00	0,00	3,27	190,37	172,94	315,25	7,79	4,26E-10	-	*Ctbp2*	XM_006507311.3	=
3,15	0,89	1,09	822,85	1,00	31,17	7,39	1,70E-02	-	*Cttnbp2*	XM_006505104.2	=
7,36	1141,02	1075,22	8,32	22,99	1,78	-6,07	1,54E-02	+	*Cul2*	XM_006526275.3	=
4,20	70,93	68,63	2,08	5817,83	7881,25	6,57	4,62E-02	-	*Cul3*	XM_006496497.3	=
816,67	76,25	510,92	40,57	46,98	38,29	-3,48	1,06E-02	-	*Cwc22*	XM_006500434.1	=
2,10	0,89	2,18	44,73	44,98	32,06	4,60	2,00E-07	-	*Cwc22*	NM_172667.2	=
2,10	0,89	2,18	47,85	52,98	37,40	4,78	2,97E-08	-	*Cwc22*	NM_001290740.1	=
1,05	0,89	2,18	39,53	41,98	33,84	4,84	2,89E-07	-	*Cwc22*	NM_030560.5	=
92,49	78,90	46,84	14,56	8,00	8,91	-2,80	8,40E-06	+	*Cxxc4*	XM_006501561.2	=
0,00	6,21	4,36	59,30	266,90	10,69	4,99	7,40E-02	-	*Cyb561*	XM_006532133.3	=
0,00	0,89	42,49	132,11	1596,40	2459,66	6,60	5,74E-02	+	*Cyfip1*	NM_001164661.1	=
6,31	10,64	25,06	0,00	0,00	0,00	-6,28	9,27E-03	+	*Cyp4f37*	NM_001100187.1	=
6,31	8,87	7,63	0,00	0,00	0,00	-5,41	7,41E-03	-	*Cyp7b1*	XM_006535384.2	=
0,00	0,00	0,00	6,24	7,00	2,67	4,84	9,63E-02	+	*D030040B21Rik*	NR_037998.1	=
4,20	1,77	1,09	221,58	2,00	284,08	6,17	9,91E-03	+	*D11Wsu47e*	XM_011249060.2	=
0,00	0,00	0,00	17,68	12,00	10,69	6,18	5,27E-04	+	*D230017M19Rik*	XR_865520.2	=
1,05	2,66	4,36	88,42	74,97	59,67	4,80	1,91E-11	-	*D5Ertd579e*	XM_011240745.2	=
1,05	0,00	0,00	64,50	73,97	1,78	7,02	2,93E-02	-	*D5Ertd579e*	XM_011240743.2	=
603,30	601,10	616,59	2021,24	2252,16	1837,18	1,75	3,55E-35	-	*Dact2*	NM_172826.3	=
0,00	0,00	0,00	11,44	9,00	4,45	5,48	1,83E-02	-	*Dcdc2b*	XM_006502631.3	=
291,14	422,90	262,54	2885,70	3135,83	367,79	2,71	8,83E-02	+	*Dcp1a*	XM_006519656.2	=
0,00	0,00	0,00	421,31	511,81	229,76	11,03	1,63E-13	+	*Dctn1*	XM_006505491.3	=
7,36	2,66	6,54	37,45	362,86	16,92	4,66	2,75E-02	+	*Dcun1d4*	XM_006503637.3	=
39,94	54,97	57,74	280,87	73,97	289,42	2,08	7,58E-02	-	*Ddr1*	XM_006523535.3	=
79,88	1054,14	1286,56	61,38	111,96	91,73	-3,19	8,65E-02	-	*Ddr2*	NM_022563.2	=
8784,68	11323,31	14355,83	0,00	0,00	0,00	-15,97	1,74E-33	-	*Ddx17*	XM_006521290.3	=
900,75	953,95	1039,27	0,00	0,00	0,00	-12,39	6,71E-22	+	*Ddx31*	XM_006497923.1	=
2,10	10,64	8,72	298,56	3,00	202,15	4,55	8,94E-02	-	*Deaf1*	XM_017322370.1	=
3,15	9,75	5,45	20,81	287,89	431,91	5,33	9,48E-04	+	*Def8*	NM_001253784.1	=
0,00	0,00	0,00	212,21	241,91	251,13	10,31	9,69E-15	+	*Def8*	XM_006530977.3	=
0,00	0,00	0,00	0,00	3325,76	158,52	25,79	1,14E-08	+	*Def8*	XM_006530975.2	=
1,05	36,35	72,99	0,00	0,00	0,00	-7,68	6,56E-02	+	*Dennd2c*	XR_375532.3	=
233,33	927,36	162,32	4,16	4,00	2,67	-6,94	4,74E-10	+	*Dennd2c*	XM_006501602.2	=
1,05	179,09	11,98	0,00	0,00	0,00	-8,48	8,65E-02	+	*Dennd4b*	XR_375516.3	=
110,36	6,21	129,64	6,24	4,00	8,01	-3,75	6,21E-02	-	*Derl2*	NM_001291147.1	=
127,18	200,37	195,00	43,69	69,97	28,50	-1,88	7,64E-03	+	*Dgkb*	XM_006515067.2	=
0,00	0,00	0,00	16,64	54,98	11,58	7,23	2,30E-03	+	*Dgkb*	NM_178681.4	=
0,00	0,00	0,00	131,07	4,00	48,98	8,37	1,09E-02	+	*Dgkb*	XM_006515070.2	=
2450,00	585,14	693,93	0,00	0,00	0,89	-11,80	1,15E-11	-	*Dgkz*	XM_006498541.2	=
1335,89	1837,87	1427,09	1,04	1,00	0,89	-10,62	1,72E-29	-	*Dgkz*	XM_017314867.1	=
1410,51	1041,72	1568,71	1,04	1,00	34,73	-6,77	3,28E-04	-	*Dgkz*	NM_138306.2	=
3873,12	2312,18	4333,55	44,73	73,97	0,89	-6,46	8,46E-05	-	*Dgkz*	XM_006498543.2	=
502,40	374,13	371,48	11,44	6,00	0,89	-6,11	6,16E-12	-	*Dgkz*	XM_006498538.2	=
12,61	282,82	290,86	3,12	24,99	8,91	-3,99	9,79E-02	-	*Dgkz*	XR_374381.2	=
224,92	211,00	236,40	11,44	10,00	46,31	-3,30	1,01E-03	-	*Dgkz*	XM_006498539.1	=
16,82	13,30	27,23	654,33	13,99	685,71	4,56	2,55E-02	+	*Dhps*	XR_001778449.1	=
2,10	0,89	2,18	95,70	33,99	3,56	4,70	3,73E-02	+	*Dhrs13*	XM_011249255.2	=
13,66	1,77	4,36	549,26	436,84	126,46	5,82	1,29E-06	+	*Dhx16*	XM_006524873.1	=
0,00	0,00	0,00	8,32	6,00	40,96	6,64	3,05E-02	-	*Dhx32*	XM_006507137.1	=
719,97	1872,44	3679,92	185,17	291,89	345,53	-2,93	5,27E-03	-	*Dis3l*	NM_001001295.2	=
189,19	406,05	434,66	3,12	0,00	0,00	-8,43	5,38E-10	-	*Dixdc1*	XM_006510457.3	=
85,14	117,03	175,39	514,93	448,83	681,26	2,12	2,76E-05	+	*Dlg1*	XM_017316872.1	=
209,16	196,82	239,66	564,86	306,89	501,37	1,09	5,78E-02	-	*Dmtn*	XM_011244949.2	=
0,00	578,93	1896,61	0,00	0,00	0,00	-12,17	7,90E-02	+	*Dopey2*	XR_001781858.1	=
0,00	0,00	0,00	0,00	2990,88	3247,79	13,46	3,29E-02	+	*Dot1l*	XM_006513430.1	=
1229,73	1171,16	1399,85	0,00	0,00	0,00	-12,79	3,66E-23	-	*Dpy19l3*	XM_006539906.1	=
62,01	1,77	34,86	0,00	0,00	0,00	-7,52	4,10E-02	+	*Dpysl4*	XM_006536195.1	=
757,81	3,55	726,62	5,20	8,00	2,67	-6,55	3,11E-03	+	*Dstyk*	XM_006529356.3	=
0,00	0,00	0,00	6,24	18,99	3,56	5,70	7,14E-02	-	*Duoxa1*	NM_001305262.1	=
57,81	76,25	25,06	0,00	0,00	0,00	-8,21	3,54E-06	+	*Duxbl1*	XM_017316041.1	=
0,00	0,00	0,00	3,12	8,00	9,80	5,24	5,26E-02	-	*DXBay18*	XM_006528194.1	=

Additional file1: Table S1. Differentially expressed transcripts (*continued*)

Control1	Control2	Control3	PB1	PB2	PB3	log2FoldChange	P.adj	Strand	Gene Name	Transcript ID	Class Code
26,28	20,39	21,79	0,00	0,00	0,00	-6,99	6,18E-06	+	*Dym*	XM_006526219.3	=
367,87	141,85	211,34	22,89	31,99	24,04	-3,19	2,22E-07	-	*Dynll2*	NM_001168472.1	=
404,65	277,50	308,29	534,70	856,68	650,98	1,04	4,66E-02	+	*Dyrk1a*	NM_007890.2	=
89,34	91,32	3,27	0,00	0,00	2,67	-6,06	7,15E-02	-	*Dzip1*	XM_017316127.1	=
45,20	100,18	102,40	19,77	2,00	6,23	-3,15	3,73E-02	+	*Dzip1l*	XR_001779020.1	=
0,00	0,00	0,00	11,44	13,00	16,92	6,22	3,03E-04	+	*Dzip1l*	XR_870770.2	=
14,71	28,37	37,04	106,11	76,97	64,12	1,62	4,53E-02	+	*E2f5*	XR_001783649.1	=
81,98	72,70	86,06	135,23	222,92	159,41	1,11	1,88E-02	-	*Eci2*	XM_006516677.1	=
2,10	2,66	1,09	373,46	0,00	308,13	6,86	3,65E-02	+	*Eda*	NM_001177943.1	=
0,00	0,00	0,00	14,56	3,00	22,26	6,17	4,03E-02	-	*Efcab11*	XM_006516385.3	=
0,00	0,00	0,00	53,05	45,98	337,51	9,62	8,19E-05	+	*Efemp1*	XM_011243695.1	=
4,20	6,21	1,09	391,14	3,00	237,77	5,77	1,62E-02	-	*Efna3*	XM_017319452.1	=
316,37	0,89	144,89	0,00	0,00	0,00	-9,75	1,98E-02	-	*Efs*	XM_006518518.3	=
63,06	55,85	86,06	18,72	20,99	17,81	-1,83	3,39E-05	+	*Eif4ebp3*	NM_201256.4	=
3,15	98,41	13,07	2,08	2,00	0,89	-4,54	9,15E-02	+	*Eif4g1*	XM_006521939.1	=
4668,77	7556,26	387,82	280,87	169,94	536,99	-3,67	5,02E-02	+	*Eif5*	NM_178041.2	=
56,76	62,06	192,82	19,77	21,99	16,92	-2,41	2,60E-02	-	*Elf3*	XM_006529124.1	=
154,50	3,55	423,77	0,00	3,00	0,00	-7,61	2,83E-02	-	*Endod1*	XM_017313601.1	=
6,31	2,66	3,27	138,36	124,95	7,12	4,47	1,59E-02	+	*Enox1*	XM_006518999.3	=
0,00	4,43	7,63	96,74	134,95	40,07	4,50	3,86E-03	+	*Enox1*	XM_006519005.3	=
6,31	2,66	3,27	282,95	344,87	6,23	5,70	3,69E-03	+	*Enox1*	XM_011245066.2	=
0,00	0,00	0,00	14,56	12,00	24,94	6,54	3,38E-04	-	*Enthd1*	XM_017316685.1	=
27,33	100,18	307,20	6,24	25,99	5,34	-3,53	9,10E-02	+	*Entpd1*	XM_006526618.3	=
0,00	0,00	1,09	7,28	34,99	6,23	5,49	5,61E-02	+	*Epb41l3*	XM_006523615.2	=
0,00	2,66	3,27	54,09	82,97	84,60	5,21	1,25E-09	-	*Epn2*	XM_006532167.3	=
0,00	0,00	0,00	49,93	60,98	44,53	8,13	1,69E-08	-	*Epn2*	XM_011248711.2	=
486,64	69,15	99,13	0,00	0,00	0,00	-10,25	5,83E-06	+	*Erc2*	XM_006518932.3	=
23,12	31,03	52,29	0,00	0,00	0,00	-7,63	5,82E-06	+	*Erich2*	XM_006500027.3	=
0,00	0,00	0,00	31,21	29,99	24,04	7,26	1,53E-06	+	*Erich2*	NM_025744.2	=
3,15	10,64	9,80	0,00	0,00	0,00	-5,46	4,18E-02	-	*Erp27*	XM_006506555.2	=
2,10	6,21	5,45	1556,24	0,00	2237,92	8,11	1,20E-02	-	*Esf1*	XM_006500011.3	=
448,80	200,37	683,04	96,74	80,97	87,27	-2,33	3,20E-03	-	*Espn*	XM_006539063.2	=
0,00	0,00	0,00	45,77	97,96	1,78	8,04	3,24E-02	-	*Esrp1*	NM_001290383.1	=
1,05	7,09	7,63	204,93	11,00	216,40	4,77	1,70E-02	+	*Esyt2*	XR_001780467.1	=
0,00	0,00	0,00	1630,10	0,00	1350,05	12,39	6,92E-02	+	*Etl4*	XM_006497415.3	=
8,41	0,00	8,72	35,37	2987,88	3466,86	8,57	4,12E-04	+	*Etv6*	XM_011241206.2	=
63,06	67,38	99,13	310,00	665,75	544,12	2,73	2,37E-07	-	*Evc*	XM_006504042.3	=
0,00	1,77	0,00	4286,93	0,00	4452,68	12,23	1,44E-02	-	*Exoc2*	XM_006516726.2	=
2,10	10,64	94,78	1,04	0,00	0,00	-6,68	7,56E-02	-	*Fam120a*	XR_001780774.1	=
1,05	0,00	0,00	10,40	8,00	15,14	4,96	1,60E-02	+	*Fam129a*	XM_006529776.3	=
63,06	47,87	71,90	26,01	15,99	13,36	-1,73	3,69E-03	+	*Fam129c*	XM_017312491.1	=
32,58	7,98	9,80	99,87	61,98	91,73	2,34	4,88E-02	+	*Fam129c*	XM_006509531.3	=
777,78	959,27	896,56	0,00	2,00	0,89	-9,82	3,54E-26	-	*Fam13b*	XM_006525849.3	=
4,20	14,19	8,72	4615,66	51,98	373,13	7,54	1,48E-04	-	*Fam160a2*	XM_006508251.3	=
344,74	2016,95	3272,49	234,06	276,90	143,38	-3,11	3,20E-02	+	*Fam168a*	XM_006507919.2	=
331,08	3373,41	3419,56	106,11	122,95	218,18	-3,99	3,40E-03	+	*Fam172a*	XM_006517350.3	=
4,20	16,84	20,70	291,27	185,93	26,72	3,59	4,62E-02	+	*Fam172a*	XM_017315582.1	=
0,00	0,00	0,00	3911,40	3772,59	0,00	13,76	2,64E-02	+	*Fam172a*	XM_017315581.1	=
236,49	280,16	66,45	0,00	0,00	0,00	-10,08	3,66E-08	-	*Fam184a*	XM_006512897.3	=
109,31	27,48	94,78	0,00	0,00	0,00	-8,75	4,15E-06	+	*Fam188b*	XM_006506296.3	=
2,10	1,77	2,18	22,89	23,99	137,14	4,93	1,63E-03	-	*Fam193b*	XR_001780770.1	=
171,32	96,64	61,01	78,02	2183,18	2620,85	3,89	6,70E-02	-	*Fam210a*	XM_006525524.3	=
180,78	135,65	82,79	36,41	36,99	46,31	-1,74	4,34E-03	-	*Fam229b*	XM_006512816.3	=
130,33	157,81	190,64	311,04	346,87	354,43	1,08	1,53E-04	-	*Fam229b*	NM_183254.1	=
2,10	137,42	362,76	0,00	2,00	0,00	-7,99	2,18E-02	+	*Fam234b*	XM_017321794.1	=
16,82	21,28	23,97	8,32	6,00	5,34	-1,67	3,74E-02	-	*Fam43b*	NM_001081672.2	=
889,19	0,00	1129,69	0,00	0,00	0,00	-11,87	9,44E-02	-	*Fam98a*	XM_017317659.1	=
70,42	0,89	72,99	0,00	0,00	0,00	-8,07	5,31E-02	+	*Farp1*	XM_006518914.3	=
366,82	375,02	373,66	1211,91	651,76	954,66	1,34	2,37E-03	-	*Fbf1*	XM_011248949.1	=
142,94	111,71	167,76	0,00	0,00	0,00	-9,62	7,04E-12	+	*Fbxo2*	XM_006538814.3	=
93,54	266,86	46,84	5,20	11,00	4,45	-4,31	3,16E-04	+	*Fbxo34*	XM_006519723.3	=
122,97	116,14	78,44	815,57	694,74	162,08	2,40	4,99E-02	-	*Fbxo38*	XM_006525502.3	=
33,63	52,31	62,09	564,86	505,81	54,32	2,93	8,54E-02	-	*Fbxo38*	XM_006525497.3	=
39,94	172,00	54,47	1139,09	954,64	150,50	3,07	9,34E-02	-	*Fbxo38*	XM_006525498.1	=
8,41	1,77	5,45	142,52	112,96	4,45	4,06	8,81E-02	-	*Fbxo38*	XM_017317780.1	=
0,00	0,00	0,00	360,97	391,85	1385,67	11,91	8,82E-11	-	*Fbxw2*	XM_006498118.3	=
424,62	346,65	375,84	3914,52	399,85	4746,56	2,98	6,94E-02	+	*Fbxw7*	XM_006501656.3	=
0,00	0,00	0,00	36,41	16,99	33,84	7,30	1,75E-05	+	*Fer1l5*	XM_006495602.2	=
1,05	216,32	76,26	0,00	0,00	0,00	-9,09	2,94E-02	-	*Fgf11*	XM_011248725.2	=
1,05	0,00	1,09	163,32	106,96	98,85	7,52	4,66E-09	-	*Fgf11*	NM_010198.2	=
31,53	170,22	249,47	27,05	10,00	13,36	-3,16	3,74E-02	-	*Fgfr2*	XM_011241673.2	=
17,87	9,75	13,07	1,04	1,00	0,00	-4,37	1,36E-02	+	*Fgfr3*	XM_006503715.3	=
2,10	4,43	0,00	27,05	22,99	21,37	3,38	8,49E-04	-	*Fkbp6*	XM_017321189.1	=
0,00	0,89	0,00	17,68	21,99	6,23	5,41	1,65E-02	+	*Fkbp8*	XR_001778406.1	=
3824,77	0,00	3919,58	0,00	0,00	0,00	-13,81	2,53E-02	-	*Fkrp*	NM_173430.2	=
5,26	3,55	2,18	109,23	115,96	42,75	4,61	2,01E-07	+	*Flrt2*	XM_011244132.2	=
0,00	0,00	0,00	8,32	14,99	6,23	5,73	8,49E-03	-	*Flt3l*	XM_006540608.3	=
0,00	0,00	0,00	364,09	256,90	128,24	10,40	9,95E-11	-	*Folr1*	XM_006507363.3	=
0,00	0,00	0,00	23,93	21,99	49,87	7,43	2,44E-05	-	*Fosb*	XM_006539544.3	=
47,30	49,65	159,05	0,00	0,00	0,00	-8,89	3,46E-06	-	*Foxa1*	NM_008259.3	=
1,05	0,00	0,00	61,38	40,98	48,98	7,13	2,37E-06	-	*Foxa1*	XM_006515483.1	=
80,93	88,66	131,81	36,41	43,98	48,98	-1,22	2,41E-02	-	*Foxj1*	XM_006532269.2	=
6,31	1745,66	872,59	14,56	2,00	9,80	-6,64	4,61E-03	+	*Foxn2*	XM_006523653.3	=
2,10	3,55	3,27	146,68	157,94	6,23	5,12	5,30E-03	-	*Foxp1*	XM_006505319.1	=
4068,62	11,53	244,02	45,77	21,99	8,91	-5,82	4,37E-02	+	*Fras1*	XM_006534873.3	=

Additional file1: Table S1. Differentially expressed transcripts (*continued*)

Control1	Control2	Control3	PB1	PB2	PB3	log2FoldChange	P.adj	Strand	Gene Name	Transcript ID	Class Code
5,26	4,43	4,36	0,00	0,00	0,00	-4,71	6,30E-02	-	*Frem1*	NM_177863.4	=
2,10	1,77	0,00	1,04	2892,92	3397,40	10,66	2,38E-04	-	*Frem1*	XM_006538015.1	=
0,00	0,00	0,00	0,00	3048,86	2091,87	13,18	4,01E-02	-	*Frem1*	XM_006538018.2	=
627,48	139,19	385,64	48,89	3,00	4,45	-4,36	3,49E-02	+	*Frmd4a*	NM_001177843.1	=
92,49	78,90	116,56	0,00	0,00	0,00	-9,06	1,54E-10	-	*Frmd5*	XM_017317740.1	=
739,94	789,94	27,23	19,77	1,00	0,89	-6,17	1,37E-02	+	*Frmd6*	XM_006515977.3	=
0,00	0,00	1,09	163,32	1708,36	3,56	10,76	1,33E-03	-	*Fundc1*	NM_001313746.1	=
55,71	256,22	30,50	1389,79	808,70	1205,79	3,31	2,40E-02	+	*Fut8*	NM_001252614.1	=
0,00	0,00	0,00	2,08	37,99	52,54	7,38	3,60E-02	+	*Gabrq*	XM_017318583.1	=
29,43	70,04	35,95	329,76	340,87	84,60	2,48	4,91E-02	+	*Gale*	NM_178389.3	=
1,05	0,89	1,09	33,29	47,98	44,53	5,39	8,63E-07	-	*Gapvd1*	XM_017319197.1	=
5,26	3,55	3,27	33,29	239,91	162,97	5,18	1,13E-05	-	*Garnl3*	XM_006498484.3	=
9,46	1,77	0,00	78,02	147,94	387,38	5,78	2,06E-03	-	*Garnl3*	XR_001783216.1	=
0,00	0,00	0,00	21,85	9,00	27,61	6,72	8,43E-04	-	*Gbp9*	XM_006534926.3	=
0,00	0,00	0,00	163,32	29,99	54,32	8,80	3,61E-05	+	*Gcnt4*	XM_006517601.3	=
16,82	24,82	45,75	0,00	0,00	0,00	-7,34	5,73E-05	+	*Gdf9*	XM_006532221.3	=
28,38	27,48	54,47	0,00	0,00	0,00	-7,68	3,62E-06	+	*Gemin8*	NM_001310724.1	=
12,61	22,16	4,36	130,03	114,96	76,59	3,02	8,38E-04	+	*Gemin8*	NM_001310722.1	=
10,51	15,07	15,25	29,13	34,99	47,20	1,45	3,11E-02	-	*Ggact*	XM_017315990.1	=
1,05	0,89	1,09	1518,79	1,00	1569,13	10,00	8,37E-05	+	*Gigyf1*	NM_031408.2	=
320,57	461,02	431,39	9450,81	9341,51	820,18	4,02	2,79E-03	+	*Gigyf2*	XM_006529464.2	=
178,68	1070,09	2078,53	67,62	77,97	60,56	-4,01	1,62E-03	-	*Git2*	XM_006530334.3	=
293,24	182,63	193,91	511,81	406,85	442,60	1,02	3,13E-02	-	*Gjc1*	NM_001159383.1	=
1,05	0,00	0,00	1,04	1646,38	139,81	10,69	4,23E-03	+	*Glcci1*	NM_001286728.1	=
252,25	532,83	2,18	4,16	5,00	1,78	-6,17	8,97E-03	-	*Gli2*	XM_011247924.2	=
28,38	46,99	49,02	1,04	2,00	0,89	-5,00	1,15E-06	-	*Glis3*	XM_006526987.3	=
0,00	0,89	0,00	2558,01	881,67	0,00	11,64	1,90E-02	-	*Gltscr1l*	XM_017317377.1	=
3,15	5,32	8,72	0,00	0,00	0,00	-5,00	7,39E-02	+	*Glyat*	NM_145935.3	=
0,00	0,00	0,00	18,72	16,99	32,06	6,94	4,77E-05	+	*Gm10419*	XR_880553.2	=
3,15	6,21	6,54	32,25	21,99	28,50	2,37	7,25E-04	-	*Gm13941*	XM_006500506.2	=
28,38	11,53	25,06	0,00	0,00	3,56	-4,12	7,51E-02	+	*Gm14321*	XR_001783578.1	=
174,47	325,37	154,69	0,00	0,00	0,00	-10,25	1,84E-11	-	*Gm14391*	XM_017319172.1	=
0,00	3,55	0,00	12,48	17,99	17,81	3,66	6,24E-02	-	*Gm16341*	XR_001779056.1	=
0,00	0,00	0,00	6,24	6,00	6,23	5,06	1,90E-02	+	*Gm16432*	XM_017321745.1	=
0,00	0,00	0,00	10,40	3,00	6,23	5,14	7,43E-02	+	*Gm16432*	XR_001785174.1	=
4,20	4,43	6,54	0,00	0,00	0,00	-4,81	5,67E-02	+	*Gm17197*	XR_386818.2	=
1446,25	895,44	448,82	6836,63	7193,31	4312,87	2,72	2,85E-04	-	*Gm21596*	XM_003945339.3	=
43,09	61,17	35,95	21,85	19,99	17,81	-1,24	5,83E-02	-	*Gm2560*	NR_131155.1	=
39,94	33,69	51,20	20,81	12,00	14,25	-1,41	3,35E-02	+	*Gm30062*	XR_880371.1	=
6,31	4,43	7,63	0,00	0,00	0,00	-5,09	2,75E-02	+	*Gm30719*	XR_373204.2	=
25,23	14,19	26,15	6,24	6,00	6,23	-1,82	4,14E-02	+	*Gm30767*	XR_390830.3	=
6,31	14,19	4,36	0,00	0,00	0,00	-5,54	3,65E-02	+	*Gm30798*	XR_387001.2	=
5,26	6,21	6,54	14,56	24,99	28,50	1,92	2,98E-02	-	*Gm31175*	XR_876270.1	=
2,10	0,89	2,18	15,60	32,99	19,59	3,76	1,10E-03	-	*Gm31204*	XR_866967.2	=
65,17	47,87	39,22	9,36	22,99	10,69	-1,82	3,43E-02	+	*Gm32051*	XR_389452.2	=
0,00	0,89	4,36	19,77	12,00	13,36	3,14	6,77E-02	-	*Gm32229*	XR_865319.2	=
0,00	0,00	0,00	5,20	7,00	5,34	4,98	2,80E-02	+	*Gm32252*	XR_385562.2	=
98,80	132,10	156,87	0,00	0,00	0,00	-9,49	2,31E-11	-	*Gm32443*	XR_381424.2	=
4,20	6,21	10,89	0,00	0,00	0,00	-5,30	3,24E-02	-	*Gm32900*	XR_001783938.1	=
14,71	15,96	14,16	0,00	0,00	0,00	-6,38	9,53E-05	+	*Gm33185*	XR_867014.2	=
8,41	2,66	6,54	0,00	0,00	0,00	-5,02	8,65E-02	-	*Gm33370*	XM_017321220.1	=
0,00	0,00	0,00	6,24	6,00	6,23	5,06	1,90E-02	-	*Gm34189*	XR_381688.3	=
10,51	7,98	11,98	0,00	0,00	0,00	-5,82	1,70E-03	+	*Gm34253*	XR_880229.1	=
0,00	0,00	0,00	10,40	11,00	13,36	5,97	6,86E-04	+	*Gm34752*	XR_377576.3	=
0,00	0,00	0,00	13,52	2,00	12,47	5,66	9,45E-02	-	*Gm35025*	XR_871161.2	=
0,00	0,00	0,00	8,32	6,00	33,84	6,44	2,66E-02	+	*Gm35129*	XR_375189.2	=
11,56	15,07	10,89	46,81	31,99	40,96	1,66	2,26E-03	+	*Gm35248*	XR_001781641.1	=
0,00	0,00	0,00	8,32	8,00	2,67	5,09	7,90E-02	-	*Gm36313*	XR_001779748.1	=
0,00	0,00	0,00	44,73	32,99	53,43	7,89	1,60E-07	+	*Gm36529*	XR_382637.2	=
2,10	6,21	0,00	27,05	24,99	25,83	3,17	1,59E-02	-	*Gm3740*	XR_375773.3	=
0,00	0,00	0,00	4,16	4,00	14,25	5,34	9,09E-02	-	*Gm38523*	XR_388026.2	=
68,32	50,53	132,90	27,05	16,99	28,50	-1,79	6,57E-02	-	*Gm38708*	XR_001785302.1	=
10,51	9,75	8,72	0,00	0,00	0,00	-5,75	1,65E-03	+	*Gm39250*	XR_879192.2	=
3,15	0,00	0,00	19,77	16,99	9,80	3,92	6,52E-02	-	*Gm39983*	XR_001783551.1	=
0,00	0,00	0,00	11,44	10,00	13,36	5,97	7,06E-04	+	*Gm40362*	XR_872643.2	=
0,00	0,00	0,00	6,24	6,00	5,34	4,98	2,48E-02	-	*Gm40960*	XR_873492.1	=
91,44	18,62	64,27	0,00	0,00	0,00	-8,34	4,45E-05	+	*Gm41168*	XR_874678.1	=
0,00	0,00	0,00	3,12	9,00	8,01	5,19	5,26E-02	-	*Gm41307*	XR_001781664.1	=
3,15	8,87	13,07	0,00	0,00	0,00	-5,54	4,54E-02	+	*Gm41935*	XR_001779349.1	=
70,42	30,14	13,07	0,00	0,00	0,00	-7,72	5,40E-04	+	*Gm4419*	XR_381323.2	=
91,44	172,88	166,67	400,50	358,87	322,37	1,33	1,28E-02	-	*Gm4969*	XM_006540471.2	=
3,15	3,55	0,00	16,64	11,00	13,36	2,57	6,02E-02	-	*Gm5782*	XR_879807.2	=
0,00	0,00	0,00	29,13	47,98	9,80	7,29	9,96E-04	-	*Gm6288*	XR_378182.3	=
9,46	7,09	8,72	0,00	0,00	0,00	-5,55	4,02E-03	+	*Gm6568*	NR_040420.2	=
29,43	21,28	19,61	9,36	8,00	6,23	-1,58	4,23E-02	+	*Gm6792*	XM_011250665.2	=
300,60	387,43	354,05	8,32	5,00	0,89	-6,23	2,04E-19	-	*Gm7072*	XR_001782223.1	=
1,05	0,00	7,63	63,46	52,98	73,91	4,49	9,90E-03	-	*Gm773*	XM_006528099.2	=
4,20	7,98	6,54	0,00	0,00	0,00	-5,13	2,88E-02	+	*Gm9994*	XM_006529052.3	=
15,77	12,41	4,36	414,03	187,93	137,14	4,50	4,52E-07	+	*Gnb1l*	NM_001081682.2	=
12,61	5,32	15,25	28,09	44,98	37,40	1,75	5,40E-02	-	*Gne*	XM_017320310.1	=
9750,59	4761,78	10872,00	2671,40	1523,43	1264,56	-2,22	3,27E-03	+	*Golga4*	NM_018748.3	=
120,87	79,79	232,04	2061,81	598,78	801,48	3,00	4,60E-03	+	*Golga4*	XR_871211.2	=
27,33	23,94	26,15	0,00	0,00	0,00	-7,17	1,96E-06	-	*Gp6*	XM_006539910.2	=
84,08	71,81	413,96	11,44	9,00	2,67	-4,63	9,08E-04	-	*Gpatch8*	XM_017314539.1	=
63,06	10,64	18,52	0,00	0,00	0,00	-7,42	2,76E-03	-	*Gpr19*	NR_072990.2	=

Additional file1: Table S1. Differentially expressed transcripts (*continued*)

Control1	Control2	Control3	PB1	PB2	PB3	log2FoldChange	P.adj	Strand	Gene Name	Transcript ID	Class Code
87,24	750,93	667,79	91,54	76,97	59,67	-2,72	8,39E-02	+	*Gps2*	NM_019726.3	=
318,47	1,77	793,07	0,00	3,00	0,00	-8,54	2,08E-02	-	*Greb1*	XM_006515095.3	=
14,71	16,84	17,43	0,00	0,00	0,00	-6,51	5,50E-05	-	*Grifin*	NM_030022.1	=
29,43	43,44	2,18	0,00	0,00	0,00	-7,13	3,87E-02	+	*Grip1*	XM_017314126.1	=
0,00	2,66	2,18	790,60	0,00	1423,97	8,83	3,00E-02	+	*Gse1*	NM_001145897.1	=
122,97	25,71	26,15	0,00	0,00	0,00	-8,34	2,98E-04	-	*Gtf2h2*	XR_873758.2	=
0,00	0,89	1,09	201,81	1,00	170,98	7,56	1,22E-02	-	*Gtf2i*	XR_001784619.1	=
77,78	0,00	0,00	18290,99	11475,71	17292,44	9,24	9,99E-03	-	*Gtf2i*	XR_001784610.1	=
5,26	268,63	245,11	7,28	9,00	8,01	-4,42	3,73E-02	+	*Gtpbp2*	XM_006524652.3	=
2,10	2,66	2,18	641,84	1,00	26,72	6,59	2,98E-02	+	*Hadhb*	NM_001289798.1	=
42,04	118,80	106,76	0,00	0,00	0,00	-8,96	1,28E-07	-	*Haghl*	NM_001271435.1	=
14,71	30,14	76,26	0,00	0,00	0,00	-7,81	4,00E-04	-	*Hax1*	NM_001282032.1	=
26117,55	1767,83	27055,81	295,44	1738,35	1796,21	-3,84	6,13E-02	-	*Hcfc1*	XM_017318387.1	=
1,05	0,00	0,00	399,46	546,80	0,00	9,77	5,56E-02	-	*Hdac7*	XM_006521208.3	=
817,72	1025,76	764,74	375,54	422,84	380,26	-1,15	9,05E-07	+	*Hdhd2*	NM_029826.2	=
854,50	1150,77	1331,22	488,92	584,78	530,76	-1,06	3,29E-03	+	*Hdhd2*	XM_006526442.3	=
951,20	940,65	906,36	15719,45	923,65	20118,11	3,72	3,15E-02	-	*Hdlbp*	NM_001301364.1	=
219,67	12,41	470,61	2,08	3,00	10,69	-5,47	6,18E-03	-	*Hebp1*	XM_011241224.2	=
7,36	1,77	0,00	676,17	728,73	0,89	7,27	3,51E-02	-	*Hecw2*	XM_006496066.3	=
6,31	8,87	10,89	0,00	0,00	0,00	-5,60	4,99E-03	+	*Hhipl2*	NM_030175.5	=
74,62	117,03	1,09	1,04	0,00	0,89	-6,64	2,95E-02	-	*Hic1*	XM_006532275.1	=
3,15	1,77	3,27	293,35	52,98	307,24	6,33	4,85E-08	-	*Hinfp*	XM_006509900.2	=
420,42	328,03	1,09	0,00	1,00	0,00	-9,48	5,05E-03	+	*Hip1r*	XM_006530373.2	=
1090,99	179,09	3,27	8,32	10,00	9,80	-5,50	3,34E-02	+	*Hira*	XM_006521802.3	=
1747,90	2232,39	1339,94	157,08	139,95	9,80	-4,12	4,97E-03	+	*Hmgcs1*	NM_001291439.1	=
2920,87	3217,37	4041,60	1362,75	1259,53	730,24	-1,60	1,10E-03	+	*Hmgcs1*	NM_145942.5	=
17940,38	16798,78	16501,91	5455,15	7129,34	7687,11	-1,34	5,92E-09	+	*Hmgcs1*	XM_006517578.1	=
468,77	201,25	434,66	3605,56	496,81	3876,51	2,85	7,12E-02	+	*Hmgxb4*	NM_001316753.1	=
3,15	1,77	0,00	864,46	151,94	1401,70	8,93	2,17E-09	-	*Homez*	NM_001177705.1	=
64,11	67,38	26,15	12,48	13,99	8,91	-2,16	1,40E-02	+	*Hpcal1*	XR_872424.1	=
1506,16	1272,23	183,02	162,28	130,95	138,03	-2,78	5,32E-02	-	*Hras*	XR_389950.2	=
113,51	231,40	104,58	33,29	25,99	40,07	-2,18	1,50E-03	-	*Hras*	NM_001130443.1	=
0,00	0,00	2,18	1327,38	0,00	1019,66	10,09	6,58E-02	-	*Hs6st2*	XM_006541519.3	=
3,15	45,22	130,73	0,00	1,00	0,89	-6,54	1,56E-02	-	*Hsdl1*	XM_006531397.3	=
5,26	9,75	6,54	184,13	9,00	186,12	4,13	2,48E-02	-	*Hsf2bp*	XM_017317700.1	=
13,66	19,50	21,79	558,62	347,87	372,24	4,54	1,00E-26	-	*Hspa12a*	NM_001327998.1	=
25,23	24,82	19,61	31,21	194,93	432,80	3,24	5,21E-02	+	*Hspa4l*	XM_006500766.3	=
2,10	179,09	305,03	0,00	3,00	0,00	-7,35	4,54E-02	+	*Htatip2*	NM_001146049.1	=
1056,31	825,40	459,72	122,75	15,99	66,79	-3,51	3,68E-03	-	*Htra3*	XM_011240796.2	=
0,00	0,00	0,00	10383,93	7471,21	0,00	27,84	3,87E-10	+	*Htt*	XM_006503744.2	=
2024,32	0,00	2001,19	0,00	0,00	0,00	-12,87	5,01E-02	+	*Hunk*	XR_384941.2	=
367,87	310,30	197,18	104,03	26,99	74,81	-2,09	5,33E-02	+	*Huwe1*	XM_011247860.1	=
0,00	0,00	0,00	331,84	587,78	24,94	10,73	4,12E-05	+	*Huwe1*	XM_006528939.2	=
174,47	476,09	46,84	38,49	29,99	16,92	-3,03	7,23E-02	-	*Iba57*	XM_011248903.2	=
0,00	0,00	0,00	2823,28	3881,55	0,00	13,56	3,05E-02	-	*Ibtk*	XM_006510766.3	=
189,19	4486,06	4512,21	60,34	41,98	73,02	-5,71	7,86E-05	-	*Ids*	NM_010498.3	=
87,24	61,17	28,32	0,00	0,00	0,00	-8,36	1,97E-06	+	*Ifit3*	XM_011247151.2	=
48,35	12,41	34,86	78,02	408,85	1060,63	4,02	1,52E-02	+	*Ift122*	XM_006506798.2	=
48,35	15,07	38,13	79,06	413,85	1247,64	4,10	1,40E-02	+	*Ift122*	XM_006506797.2	=
0,00	0,00	0,00	153,96	65,98	147,83	9,37	3,66E-09	+	*Ift140*	XM_006523408.3	=
1,05	0,00	0,00	2,08	97,96	29,39	6,90	3,84E-02	+	*Igdcc4*	XR_379432.3	=
4,20	5,32	1,09	181,01	230,91	121,11	5,62	2,03E-14	+	*Igfbp2*	NM_001310659.1	=
285,89	135,65	22,88	0,00	0,00	0,00	-9,69	6,20E-05	-	*Igsf1*	NM_177591.4	=
154,50	926,47	1042,54	37,45	50,98	68,57	-3,76	8,34E-04	+	*Igsf3*	XM_011240287.2	=
60,96	46,10	1,09	1,04	1,00	0,89	-5,21	5,03E-02	-	*Ikbkb*	XM_006509021.2	=
172,37	112,59	489,13	2,08	0,00	17,81	-5,27	3,32E-02	+	*Il17rc*	NM_178942.1	=
3,15	0,00	0,00	80,10	117,96	69,46	6,46	1,56E-07	+	*Il1r1*	XM_017317613.1	=
3,15	0,00	0,00	275,67	108,96	317,03	7,84	2,34E-07	+	*Il1r1*	XM_006495719.2	=
276,43	819,19	383,46	97,78	119,96	61,45	-2,41	5,31E-03	+	*Ilk*	XM_006507390.3	=
0,00	0,00	0,00	590,87	857,68	1070,42	12,15	3,50E-18	+	*Inadl*	XM_006502703.2	=
0,00	0,00	0,00	970,57	0,00	1804,23	12,29	7,38E-02	+	*Ints10*	XM_006509742.3	=
0,00	0,00	0,00	207,01	1,00	525,42	10,37	1,27E-02	+	*Invs*	XM_017319994.1	=
256,46	190,61	140,53	2,08	3,00	1,78	-6,43	1,77E-17	+	*Ip6k2*	XM_006511850.2	=
2415,31	2636,67	2284,43	816,61	784,71	848,68	-1,58	5,65E-42	+	*Ip6k2*	XM_006511852.2	=
22,07	8,87	43,58	203,89	193,93	195,92	3,00	4,77E-04	-	*Iqsec1*	XM_006505985.3	=
54,65	63,83	66,45	37,45	6408,61	312,58	5,19	5,75E-02	-	*Irak1*	NM_001177975.1	=
8,41	12,41	16,34	0,00	1,00	0,00	-5,15	9,51E-03	+	*Irf2*	XM_006509289.3	=
3,15	103,73	46,84	0,00	0,00	0,00	-8,16	1,52E-02	+	*Irf5*	NM_001252382.1	=
23,12	41,67	22,88	0,00	0,00	0,00	-7,35	8,76E-06	+	*Itpr1*	XM_006505629.1	=
0,00	0,00	0,00	15,60	14,99	36,51	6,92	3,21E-04	+	*Itpr1*	XM_017321407.1	=
19,97	8,87	19,61	107,15	96,96	130,91	2,81	1,39E-08	+	*Iws1*	XM_006526291.3	=
5,26	0,00	0,00	644,96	740,72	3,56	8,05	3,28E-02	+	*Iws1*	XR_386017.2	=
0,00	0,00	0,00	45,77	13,99	3,56	6,83	3,75E-02	+	*Jdp2*	XM_011244218.1	=
108,26	30,14	51,20	0,00	0,00	0,00	-8,46	5,77E-06	+	*Josd2*	NM_001205073.1	=
292,19	217,21	11,98	0,00	1,00	0,00	-8,96	2,86E-04	-	*Kalrn*	XM_011245967.2	=
255,41	187,07	7,63	0,00	1,00	0,00	-8,75	8,79E-04	-	*Kalrn*	XM_011245966.2	=
307,96	196,82	13,07	0,00	2,00	0,00	-8,03	1,62E-03	-	*Kalrn*	XM_017317074.1	=
241,74	185,29	7,63	0,00	2,00	0,00	-7,78	4,80E-03	-	*Kalrn*	XM_011245965.2	=
84,08	82,45	116,56	31,21	21,99	40,96	-1,58	2,79E-03	-	*Kansl1*	XM_006534452.3	=
83,03	91,32	126,37	355,77	253,91	435,47	1,80	6,68E-05	-	*Kansl1*	XM_006534447.3	=
0,00	0,89	0,00	471,24	361,86	0,00	9,59	6,18E-02	+	*Kat2b*	XM_006523820.3	=
3,15	0,00	3,27	80,10	57,98	125,57	5,42	2,56E-07	-	*Kcnip4*	XM_017321178.1	=
0,00	0,00	0,00	36,41	291,89	495,14	10,54	8,98E-06	-	*Kcnk2*	NM_010607.3	=
0,00	0,00	0,00	5,20	4,00	10,69	5,17	5,38E-02	-	*Kcnk4*	XM_006526718.2	=
8,41	12,41	11,98	0,00	0,00	0,00	-5,93	1,12E-03	-	*Kcnv1*	XM_006521297.3	=

Additional file1: Table S1. Differentially expressed transcripts (*continued*)

Control1	Control2	Control3	PB1	PB2	PB3	log2FoldChange	P.adj	Strand	Gene Name	Transcript ID	Class Code
59,91	48,76	76,26	2071,17	2238,16	45,42	4,56	2,00E-02	-	*Kcp*	NM_001029985.4	=
105,11	100,18	153,60	13,52	9,00	10,69	-3,44	9,76E-14	-	*Kctd15*	XM_006539903.3	=
97,75	74,47	78,44	214,29	187,93	150,50	1,14	1,75E-03	+	*Kctd6*	NM_001305936.1	=
88,29	54,97	43,58	14,56	1,00	2,67	-3,37	5,38E-02	+	*Kdm1b*	XM_006516913.3	=
12,61	16,84	13,07	5,20	3,00	3,56	-1,87	7,28E-02	+	*Khdc3*	XM_006511374.3	=
505,56	435,31	1689,63	0,00	0,00	0,00	-12,26	1,49E-11	+	*Kidins220*	XM_006515254.3	=
117,72	122,35	1014,21	0,00	0,00	0,00	-11,19	4,88E-06	+	*Kidins220*	XM_006515253.3	=
2,10	15,96	2,18	5,20	1950,27	89,05	6,65	2,73E-02	-	*Kif13a*	XM_006516861.3	=
109,31	81,56	152,51	0,00	0,00	0,00	-9,32	2,92E-10	-	*Kif16b*	XM_006498815.3	=
67,27	65,61	70,81	0,00	2,00	1,78	-5,71	7,57E-11	+	*Kif20b*	XM_006527080.2	=
235,44	247,35	6,54	0,00	0,00	0,89	-8,87	9,96E-04	+	*Kirrel3*	XM_006510557.3	=
8,41	10,64	8,72	21,85	23,99	35,62	1,55	4,29E-02	+	*Kiz*	XM_006499299.3	=
804,05	721,67	859,52	53,05	43,98	6,23	-4,53	7,00E-06	-	*Klf7*	XM_006496353.2	=
67,27	15,07	19,61	343,29	128,95	244,01	2,81	2,95E-02	+	*Klf8*	XM_017318504.1	=
182,88	101,07	96,95	0,00	0,00	0,00	-9,47	3,73E-10	+	*Klhdc1*	XM_011244121.2	=
4230,48	4690,86	448,82	267,35	339,87	257,37	-3,44	1,40E-02	-	*Klhl13*	NM_026167.4	=
16,82	4,43	19,61	44,73	125,95	64,12	2,53	5,56E-02	-	*Klhl28*	XM_006516157.3	=
5,26	40,78	114,38	3,12	1,00	0,89	-5,02	1,90E-02	+	*Klhl7*	XR_880834.2	=
3653,45	41,67	3596,04	56,17	56,98	48,09	-5,50	4,47E-03	-	*Las1l*	NM_152822.3	=
43,09	39,90	3,27	0,00	1,00	0,00	-6,36	2,39E-02	+	*Lasp1*	XM_006532342.1	=
3,15	289,02	274,52	1,04	0,00	0,89	-8,19	1,72E-03	+	*Lcor*	XR_877670.1	=
183,93	347,54	0,00	1,04	0,00	0,00	-8,99	8,25E-02	+	*Lcp1*	XM_006518699.1	=
0,00	2,66	0,00	562,78	2,00	373,13	8,42	1,61E-02	+	*Lhfpl2*	XM_011244643.2	=
3,15	8,87	0,00	1922,41	4,00	890,54	7,86	8,24E-03	+	*Limch1*	XM_006504182.3	=
2189,34	1802,40	1598,12	2859,69	7539,18	8712,12	1,77	5,01E-02	+	*Lims1*	XM_006513069.3	=
2,10	95,75	179,75	2,08	0,00	0,00	-7,09	4,23E-02	-	*Lin54*	XM_006534883.3	=
6,31	7,09	4,36	6,24	413,85	389,16	5,51	5,27E-03	+	*Lipt1*	XM_006496175.3	=
1406,31	801,46	1434,71	2,08	5,00	0,00	-9,04	1,41E-21	+	*Lmf1*	XM_006525095.3	=
98,80	3,55	65,36	0,00	0,00	0,00	-8,28	9,75E-03	-	*Lmo3*	XM_006505339.3	=
0,00	0,00	0,00	6,24	3,00	8,91	5,04	7,34E-02	-	*LOC102635252*	XR_001784052.1	=
46,25	71,81	46,84	15,60	24,99	10,69	-1,69	3,14E-02	-	*LOC102635682*	XR_866876.2	=
0,00	0,00	0,00	6,24	7,00	2,67	4,84	9,63E-02	-	*LOC102639543*	XM_006497078.1	=
14,71	5,32	8,72	0,00	0,00	0,00	-5,74	1,19E-02	+	*LOC102640059*	XR_383337.3	=
6,31	12,41	21,79	0,00	0,00	0,00	-6,23	5,37E-03	-	*LOC106557447*	NR_132734.1	=
7,36	11,53	7,63	29,13	24,99	24,94	1,56	1,87E-02	-	*LOC108167560*	XM_017313046.1	=
6,31	7,09	3,27	0,00	0,00	0,00	-4,96	5,75E-02	-	*LOC108167625*	XR_001778928.1	=
952,25	535,49	538,15	78,02	30,99	135,36	-3,05	9,48E-04	-	*LOC108168233*	XR_001781614.1	=
5,26	3,55	10,89	0,00	0,00	0,00	-5,19	6,48E-02	-	*Lpar1*	XM_011249933.2	=
0,00	0,00	0,00	96,74	185,93	92,62	9,40	1,10E-09	+	*Lrp11*	XM_006512721.3	=
14,71	6,21	17,43	0,00	0,00	0,00	-6,15	3,69E-03	-	*Lrrc16a*	XR_001780805.1	=
7,36	33,69	13,07	555,50	501,81	16,92	4,31	4,73E-02	+	*Lrrk2*	XM_006521277.3	=
78,83	83,34	111,12	46,81	14,99	20,48	-1,74	8,89E-02	+	*Lrrtm4*	XM_006506086.2	=
0,00	0,00	1,09	222,62	184,93	1,78	8,57	7,58E-03	+	*Lsamp*	XM_006522201.3	=
39,94	104,62	64,27	0,00	10,00	3,56	-3,94	3,23E-02	+	*Ltb4r1*	XM_006518666.2	=
595,95	616,17	627,48	8507,29	9656,39	496,03	3,34	7,17E-02	+	*Ltbp1*	XM_006524350.3	=
772,52	1175,60	885,66	358,89	324,88	377,59	-1,42	2,47E-06	+	*Ltbp3*	XM_006531669.2	=
459,31	660,50	593,71	218,46	213,92	242,23	-1,34	4,52E-07	+	*Ltbp3*	XM_006531671.2	=
466,67	582,48	583,91	231,98	210,92	242,23	-1,25	1,09E-09	+	*Ltbp3*	XM_006531672.2	=
357,36	392,75	369,30	171,64	171,94	173,65	-1,11	1,09E-15	+	*Ltbp3*	XM_017318071.1	=
255,41	393,64	309,38	160,20	123,95	168,31	-1,08	1,31E-02	+	*Ltbp3*	XM_006531668.2	=
360,51	345,76	350,78	178,93	168,94	170,98	-1,03	2,19E-14	+	*Ltbp3*	XM_017318072.1	=
1,05	0,89	0,00	2,08	98,96	92,62	6,62	1,68E-02	+	*Luzp1*	XR_390753.1	=
1161,41	0,00	2276,80	0,00	0,00	0,00	-12,64	5,83E-02	+	*Ly6e*	NM_001164039.1	=
914,41	0,00	1339,94	0,00	0,00	0,00	-12,03	8,59E-02	+	*Ly6e*	NM_001164038.1	=
0,00	0,00	0,00	1689,39	1979,26	0,00	12,69	5,66E-02	+	*Ly6e*	NM_001164040.1	=
101,95	102,84	84,97	1695,63	185,93	3802,59	4,29	4,37E-03	+	*Lypd6*	XM_011239116.2	=
0,00	0,00	0,00	3,12	38,99	35,62	7,13	2,17E-02	+	*Lypd6b*	XM_006498350.3	=
0,00	0,00	0,00	5,20	80,97	28,50	7,69	1,10E-02	+	*Lypd6b*	XM_006498351.3	=
99,85	1,77	237,48	0,00	3,00	0,00	-6,83	8,10E-02	+	*Lztr1*	XM_006522476.2	=
17,87	3978,05	3792,13	12,48	28,99	0,00	-7,55	1,68E-02	-	*Macf1*	XM_011240417.1	=
11,56	0,89	7,63	132,11	74,97	94,40	3,93	4,73E-04	-	*Magi1*	XM_011241222.2	=
32,58	164,02	156,87	3057,34	3010,88	167,42	4,14	3,27E-02	+	*Magi2*	NM_001170745.1	=
50,45	56,74	58,83	161,24	181,93	90,83	1,38	2,32E-02	+	*Malsu1*	XM_006506723.3	=
5593,69	5666,97	5324,88	28,09	22,99	16,03	-7,96	1,82E-217	-	*Man2a2*	XM_006540600.3	=
3343,39	2383,11	3926,12	105,07	81,97	104,19	-5,05	5,84E-56	-	*Man2a2*	XM_006540599.3	=
5736,63	5082,72	5885,91	1487,58	1358,49	988,50	-2,12	3,35E-16	+	*Man2b1*	NM_010764.2	=
924,92	1167,62	749,49	304,80	269,90	128,24	-2,02	1,68E-03	+	*Man2b1*	XM_006530747.2	=
16,82	23,94	0,00	4309,82	4329,38	4868,56	8,37	7,38E-09	+	*Man2b1*	XM_006530749.2	=
4260,96	3192,55	4245,31	1166,14	1956,27	971,58	-1,51	8,89E-03	+	*Man2c1*	NM_028636.2	=
4009,76	3446,11	0,00	0,00	0,00	0,00	-13,76	2,64E-02	-	*Map3k4*	NM_011948.2	=
1543,99	1649,91	0,00	0,00	1,00	0,00	-11,57	1,59E-02	-	*Map3k4*	XR_001782078.1	=
1726,88	1083,39	47,93	8,32	13,99	42,75	-5,45	3,22E-03	-	*Map3k4*	XR_001782077.1	=
40,99	28,37	59,92	0,00	0,00	0,00	-7,91	9,05E-07	+	*Map6*	NM_001043355.2	=
3,15	0,89	4,36	16,64	51,98	56,10	3,93	1,65E-03	+	*Map6*	XR_001785484.1	=
2,10	3,55	3,27	194,53	149,94	3,56	5,28	1,15E-02	+	*Mapk1ip1l*	XM_011245015.1	=
1412,61	1168,50	0,00	0,00	1,00	0,00	-11,27	1,94E-02	-	*Mapk8ip3*	NM_013931.4	=
3341,29	328,03	3,27	1,04	7,00	1,78	-8,55	1,08E-03	+	*Mapkap1*	NM_001290625.1	=
15,77	0,00	0,00	1232,71	32,99	2699,22	7,98	4,88E-02	+	*Mapkap1*	XM_011239083.2	=
22,07	84,22	142,71	0,00	0,00	0,00	-8,85	3,49E-05	+	*Mapt*	NM_010838.4	=
57,81	64,72	63,18	38,49	23,99	28,50	-1,03	3,21E-02	-	*Masp1*	NM_008555.2	=
472,97	292,57	288,69	63,46	56,98	43,64	-2,69	7,93E-11	-	*Mast3*	XM_006509713.3	=
7,36	5,32	15,25	52,01	22,99	33,84	1,98	9,08E-02	-	*Mast3*	XM_011242315.1	=
40,99	132,10	45,75	22,89	8,00	8,91	-2,47	8,41E-02	-	*Mastl*	XM_006498239.3	=
841,89	583,36	420,50	80,10	124,95	219,96	-2,12	1,16E-02	-	*Mbnl3*	XM_006541437.3	=
2198,80	2502,80	1,09	0,00	1,00	0,00	-12,13	3,64E-04	-	*Mboat7*	XM_006540433.3	=

Additional file1: Table S1. Differentially expressed transcripts (*continued*)

Control1	Control2	Control3	PB1	PB2	PB3	log2FoldChange	P.adj	Strand	Gene Name	Transcript ID	Class Code
922,82	1461,96	0,00	0,00	0,00	0,00	-12,11	8,17E-02	+	*Mbtd1*	XM_017314201.1	=
527,63	604,64	401,98	233,02	211,92	297,44	-1,05	1,63E-02	-	*Mcfd2*	XM_006523900.2	=
16887,23	1327,20	6,54	6,24	17,99	10,69	-9,03	2,78E-04	-	*Mcm7*	XM_006504556.3	=
0,00	0,00	0,00	2056,61	0,00	2768,68	13,09	4,27E-02	+	*Mcph1*	XM_011242058.2	=
10,51	19,50	65,36	0,00	0,00	0,00	-7,47	2,70E-03	+	*Me3*	XM_006507192.3	=
54,65	46,99	81,70	6,24	3,00	17,81	-2,75	2,25E-02	+	*Me3*	XR_001785465.1	=
0,00	0,00	0,00	96,74	73,97	81,93	8,83	2,49E-10	+	*Me3*	XM_006507189.3	=
2,10	1,77	1,09	2,08	334,87	130,02	6,55	5,20E-03	-	*Med1*	NM_013634.2	=
18,92	61,17	273,43	2411,34	1962,27	2373,28	4,26	3,11E-03	-	*Med17*	XM_006510157.3	=
0,00	0,00	0,00	2909,62	1886,30	0,00	13,08	4,29E-02	-	*Med17*	XM_006510158.3	=
0,00	0,00	0,00	57,21	78,97	49,87	8,39	1,09E-08	+	*Mef2d*	NM_001310593.1	=
318,47	336,90	523,99	150,84	143,95	135,36	-1,45	2,38E-04	+	*Mest*	NM_001252292.1	=
3001,80	2286,47	1821,44	26697,38	29868,84	28137,39	3,57	1,04E-37	+	*Mest*	XM_017321427.1	=
0,00	1,77	0,00	120,67	65,98	150,50	7,42	2,39E-07	+	*Mfap3l*	NM_001293769.1	=
0,00	0,00	0,00	212,21	17,99	32,06	8,89	1,59E-03	+	*Mfsd11*	XM_006534144.3	=
0,00	0,00	0,00	114,43	86,97	106,86	9,12	5,01E-11	+	*Mid1*	NM_001290506.1	=
126,13	47,87	62,09	1976,50	111,96	1803,34	4,04	1,78E-02	+	*Midn*	XM_006513914.2	=
43,09	1,77	38,13	1931,77	38,99	1823,82	5,52	2,48E-02	+	*Midn*	XM_017314035.1	=
178,68	147,17	150,33	68,66	39,99	87,27	-1,28	5,12E-02	+	*Mir9-3hg*	NR_040311.1	=
91,44	52,31	74,08	172,68	127,95	156,73	1,07	5,20E-02	+	*Mir9-3hg*	NR_040312.1	=
10,51	46,99	6,54	0,00	0,00	0,00	-6,90	1,89E-02	+	*Mkl2*	XM_006522108.2	=
269,07	41,67	3,27	0,00	2,00	2,67	-6,06	4,02E-02	+	*Mknk1*	XM_006502823.3	=
93,54	89,54	81,70	373,46	125,95	272,50	1,54	9,91E-02	+	*Mkrn2*	XM_011241431.1	=
141,89	34,58	43,58	209,09	1820,32	369,57	3,45	7,22E-02	-	*Mpdz*	NM_001305286.1	=
29,43	4,43	4,36	24,97	1263,53	460,41	5,52	1,28E-02	-	*Mpdz*	NM_010820.3	=
438,29	1322,77	1,09	3,12	1,00	0,89	-8,47	1,07E-03	+	*Mpeg1*	NM_010821.1	=
3,15	0,89	2,18	186,21	385,86	4,45	6,55	2,21E-03	-	*Mpp7*	NM_001081287.2	=
46,25	543,47	11,98	0,00	0,00	0,00	-10,13	2,97E-03	-	*Mppe1*	XM_011246910.2	=
79,88	30,14	62,09	0,00	0,00	0,00	-8,32	8,31E-07	-	*Mrpl15*	NR_033530.1	=
1,05	0,00	2,18	56,17	42,98	14,25	5,20	1,52E-03	+	*Mrps9*	XM_006496256.3	=
2,10	1,77	2,18	2,08	2829,94	2974,39	9,91	1,77E-05	+	*Mrrf*	NM_026422.2	=
0,00	0,00	0,00	5,20	4,00	27,61	6,06	8,64E-02	-	*Msh5*	XM_017317285.1	=
216,52	167,56	1,09	0,00	0,00	0,00	-9,48	1,84E-02	+	*Msi1*	XM_006530182.3	=
0,00	4,43	2,18	55,13	74,97	8,91	4,37	4,51E-02	+	*Mst1*	XM_006511639.3	=
5588,44	0,00	2822,58	0,00	0,00	0,00	-13,93	2,32E-02	-	*Mtcl1*	XM_006524852.3	=
0,00	0,00	0,00	142,52	228,91	16,03	9,45	1,84E-04	+	*Mtmr14*	XR_377503.3	=
3,15	7,09	5,45	84,26	75,97	8,91	3,42	4,51E-02	+	*Mtmr4*	XM_006532384.3	=
0,00	0,00	0,00	0,00	2785,96	3915,69	13,56	3,05E-02	-	*Muc16*	XM_011242635.1	=
1,05	0,89	1,09	9,36	10,00	40,96	4,33	2,69E-02	+	*Mut*	XM_017317291.1	=
5,26	5,32	1,09	7,28	2479,07	5381,51	9,39	2,04E-05	-	*Myl12a*	XM_006524822.1	=
1,05	2,66	2,18	1489,66	3,00	652,76	8,50	1,74E-04	+	*Myo1c*	NM_001080774.1	=
55,71	67,38	68,63	10,40	4,00	1,78	-3,59	7,41E-06	-	*Mysm1*	XM_017320260.1	=
0,00	0,00	1,09	169,56	0,00	1279,70	10,39	5,10E-02	-	*N4bp1*	XM_006531516.2	=
0,00	0,00	0,00	92,58	906,66	1482,74	12,13	2,02E-07	+	*N4bp2*	NM_001024917.1	=
6,31	6,21	8,72	29,13	28,99	22,26	1,93	2,72E-03	-	*N4bp3*	XM_006532765.3	=
392,04	703,94	834,46	2895,06	1294,52	1643,93	1,60	9,26E-02	+	*Naa35*	NM_030153.2	=
10,51	658,72	1237,53	0,00	0,00	0,00	-11,79	2,00E-04	+	*Nadk*	XM_006538663.3	=
1,05	0,89	0,00	4,16	11,00	61,45	5,28	6,89E-02	-	*Nadsyn1*	XM_006508655.3	=
0,00	0,00	0,00	215,33	92,97	55,21	9,36	4,12E-07	-	*Nadsyn1*	XM_011242021.2	=
124,02	41,67	45,75	0,00	0,00	0,00	-8,62	2,51E-06	+	*Nars2*	XM_017322252.1	=
101,95	28,37	43,58	11,44	8,00	14,25	-2,36	4,19E-02	+	*Nars2*	XM_006507816.1	=
1038,44	1383,05	1003,32	48,89	12,00	7,12	-5,66	5,00E-09	+	*Nbeal1*	XM_006496028.3	=
2,10	2,66	10,89	32,25	126,95	16,92	3,50	9,16E-02	+	*Nbr1*	NM_001252220.1	=
2311,26	10215,09	968,46	414,03	107,96	89,05	-4,47	7,51E-03	+	*Ncaph2*	NM_001271601.1	=
310,06	559,43	104,58	7741,66	5266,03	5182,92	4,22	8,58E-07	+	*Ncaph2*	XR_001781550.1	=
2,10	1,77	2,18	61,38	2,00	65,90	4,42	5,73E-02	+	*Nde1*	XM_011246000.1	=
63,06	77,13	101,31	120,67	1084,59	1123,86	3,27	2,26E-02	-	*Ndrg2*	NM_001145959.1	=
4,20	70,93	7,63	0,00	0,00	0,00	-7,27	5,74E-02	-	*Nebl*	XR_001783185.1	=
6,31	662,27	684,13	0,00	0,00	0,00	-11,30	5,77E-04	-	*Nectin3*	XR_001781847.1	=
152,40	103,73	203,71	0,00	0,00	0,00	-9,74	4,88E-11	-	*Neil1*	XM_006511487.3	=
0,00	0,89	0,00	19,77	171,94	2,67	7,49	3,24E-02	-	*Neil1*	XM_006511486.2	=
1923,42	0,00	881,31	0,00	0,00	0,00	-12,35	7,12E-02	+	*Nek1*	NM_175089.4	=
75,68	75,36	63,18	0,00	0,00	0,00	-8,64	5,54E-10	-	*Nek8*	XM_006532184.3	=
631,68	466,34	358,41	1399,16	1480,45	1547,75	1,60	7,62E-06	+	*Nfatc4*	NM_023699.3	=
0,00	0,00	0,00	896,71	497,81	1941,37	12,55	2,24E-13	+	*Nfx1*	XM_011250123.2	=
4,20	51,42	11,98	355,77	2890,92	516,51	5,79	6,89E-04	-	*Nfyc*	XR_001784106.1	=
4251,50	99,30	3988,22	67,62	120,95	75,70	-4,98	5,42E-03	-	*Nisch*	XM_017316116.1	=
2,10	57,63	30,50	1,04	1,00	0,89	-4,95	4,25E-02	-	*Nmral1*	NM_026393.2	=
88,29	51,42	96,95	0,00	0,00	0,00	-8,78	6,47E-09	+	*Nol9*	NM_028727.3	=
578,08	450,38	350,78	39,53	4,00	14,25	-4,58	2,78E-05	+	*Nol9*	XM_006539208.3	=
77,78	54,08	84,97	17,68	15,99	21,37	-1,97	1,64E-05	+	*Nol9*	XM_017320412.1	=
6624,77	6588,12	5453,43	12980,43	15480,22	15069,66	1,22	4,23E-11	-	*Npc2*	NM_023409.4	=
5,26	4,43	0,00	21,85	26,99	17,81	2,76	2,83E-02	-	*Npff*	NM_018787.1	=
23,12	23,94	34,86	273,59	27,99	572,61	3,42	6,94E-02	-	*Nphp1*	XM_006499904.1	=
10,51	2,66	5,45	88,42	105,96	15,14	3,50	2,82E-02	-	*Npr1*	XM_006501114.1	=
0,00	0,00	0,00	90,50	111,96	69,46	8,94	5,54E-10	-	*Nr1h3*	XM_006499168.3	=
394,14	0,89	378,01	0,00	0,00	0,00	-10,49	1,07E-02	-	*Nr2f1*	XM_006517085.3	=
0,00	0,00	2,18	8,32	13,00	31,17	4,67	6,04E-02	-	*Nr2f1*	XM_017315379.1	=
12,61	21,28	29,41	205,97	403,85	71,24	3,43	2,47E-03	-	*Nr3c1*	XM_006525660.2	=
9,46	4,43	28,32	19,77	270,90	484,45	4,20	5,17E-02	+	*Nrcam*	XM_006515944.2	=
0,00	0,00	0,00	2,08	102,96	65,90	8,27	2,16E-02	+	*Nrcam*	XM_006515949.2	=
487,69	609,08	635,11	353,69	315,88	199,48	-1,00	9,44E-02	-	*Nrg1*	XM_017312638.1	=
0,00	0,00	0,00	7,28	250,91	36,51	9,05	7,05E-03	-	*Nrg1*	XM_006509072.3	=
0,00	0,89	0,00	132,11	127,95	8,01	7,95	1,55E-03	+	*Nrp2*	NM_001077406.1	=
759,91	14,19	1212,48	5,20	11,00	16,92	-5,90	2,65E-03	+	*Nsd1*	XM_011244496.2	=

Additional file1: Table S1. Differentially expressed transcripts (*continued*)

Control1	Control2	Control3	PB1	PB2	PB3	log2FoldChange	P.adj	Strand	Gene Name	Transcript ID	Class Code
1374,77	1949,57	0,00	0,00	1,00	0,00	-11,63	1,55E-02	-	*Nsmce1*	XM_006508136.2	=
93,54	123,23	8,72	1200,47	801,70	1864,78	4,10	4,24E-03	+	*Nsun5*	NM_145414.2	=
60,96	55,85	82,79	0,00	0,00	0,89	-7,57	3,09E-07	-	*Nt5c2*	XM_017318310.1	=
5,26	0,89	0,00	187,25	70,97	23,15	5,53	7,51E-03	-	*Nt5c2*	XM_017318311.1	=
366,82	201,25	252,74	41,61	11,00	19,59	-3,51	6,18E-06	-	*Nubp2*	XM_006524308.2	=
6,31	14,19	13,07	2,08	0,00	0,00	-4,09	7,38E-02	+	*Nucb2*	XR_001785587.1	=
368,92	393,64	506,56	42,65	129,95	98,85	-2,22	2,06E-03	+	*Nudt18*	XM_006518793.2	=
35,74	83,34	69,72	257,99	121,95	472,87	2,17	7,65E-02	+	*Nxpe4*	NM_172921.3	=
3,15	6,21	19,61	618,96	357,87	291,21	5,46	4,94E-09	-	*Nyap1*	XM_006504576.3	=
58,86	32,80	65,36	135,23	239,91	200,37	1,88	2,29E-03	+	*Ocrl*	XM_017318524.1	=
36,79	6,21	71,90	0,00	0,00	0,00	-7,74	4,72E-03	+	*Odf2l*	XM_006501680.3	=
3,15	2,66	9,80	68,66	161,94	7,12	3,94	7,65E-02	+	*Ogfr*	XM_006500711.3	=
6,31	3,55	11,98	0,00	0,00	0,00	-5,34	5,18E-02	-	*Oit1*	XM_011244736.1	=
115,62	134,76	140,53	62,42	78,97	50,76	-1,03	1,22E-02	-	*Ola1*	NM_030091.1	=
0,00	0,00	0,00	105,07	63,98	77,48	8,80	1,48E-09	+	*Oma1*	XM_006503300.3	=
1193,99	2023,16	1449,96	3,12	3,00	3,56	-8,91	5,00E-47	-	*Oplah*	XM_006521508.3	=
1,05	0,89	4,36	2317,71	1,00	4471,38	10,08	1,08E-04	-	*Oplah*	XR_384054.3	=
1775,22	871,50	22,88	11,44	20,99	8,01	-6,05	1,16E-03	-	*Orc3*	XM_017320309.1	=
2871,47	4173,99	0,00	4,16	3,00	1,78	-9,62	1,54E-03	-	*Os9*	XM_011243420.1	=
2015,91	1646,37	1632,98	0,00	0,00	0,00	-13,27	1,16E-24	-	*Osbpl9*	XM_006502618.3	=
278,53	236,71	340,98	7043,64	6598,54	393,62	4,04	1,02E-02	-	*Osbpl9*	XM_006502619.3	=
474,02	466,34	449,91	0,00	0,00	0,00	-11,34	2,49E-18	-	*Otud3*	XM_017320406.1	=
211,26	160,47	307,20	864,46	500,81	1705,38	2,18	2,80E-02	+	*Otud5*	XM_006527665.3	=
21,02	17,73	79,52	4,16	2,00	1,78	-3,91	7,98E-03	+	*Oxr1*	XR_001781502.1	=
478,23	698,62	711,36	4202,67	3303,77	891,43	2,15	7,75E-02	+	*Oxr1*	NM_001130165.1	=
1,05	0,89	1,09	2319,79	2095,22	0,89	10,51	4,29E-05	+	*Oxr1*	NM_001130164.1	=
0,00	0,00	0,00	5959,68	2463,08	5434,94	14,61	5,53E-23	-	*Oxsr1*	XM_006511919.3	=
0,00	0,00	0,00	37775,16	0,00	18529,39	29,67	1,64E-11	-	*Pabpc1*	XM_011245339.2	=
550,75	280,16	357,32	14069,59	596,78	11658,90	4,47	5,82E-03	+	*Pabpc4*	NM_148917.2	=
459,31	31,92	599,16	9,36	29,99	14,25	-4,35	1,11E-02	+	*Pak1*	XM_017322028.1	=
0,00	0,00	0,00	89,46	17,99	31,17	7,96	2,76E-04	-	*Palb2*	NM_001289843.1	=
1,05	0,89	1,09	133,15	178,93	318,81	7,71	1,55E-11	-	*Pam*	XM_017319290.1	=
1,05	2,66	16,34	148,76	18,99	152,28	4,01	8,54E-02	-	*Papd7*	XM_006517185.1	=
0,00	0,00	0,00	44,73	105,96	24,94	8,31	3,16E-05	+	*Pard6a*	NM_001286345.1	=
125,08	184,41	176,48	40,57	52,98	24,94	-2,04	1,22E-04	+	*Parp11*	XM_006505231.3	=
97,75	117,91	228,77	0,00	0,00	0,00	-9,69	8,83E-10	-	*Parpbp*	XM_006514269.3	=
107,21	46,10	101,31	9,36	16,99	10,69	-2,78	1,34E-04	-	*Paxip1*	NM_018878.3	=
181,83	350,20	193,91	117,55	87,97	78,37	-1,36	6,22E-02	+	*Pbrm1*	XM_017316156.1	=
2,10	1,77	2,18	96,74	44,98	48,09	4,98	1,41E-07	-	*Pbx1*	XM_006496699.2	=
0,00	0,00	1,09	17,68	9,00	28,50	5,68	6,21E-03	-	*Pcdh1*	XM_006526324.3	=
3,15	2,66	1,09	442,11	3,00	1230,72	7,92	5,40E-04	+	*Pcif1*	XM_011239494.2	=
1107,81	1883,97	0,00	0,00	0,00	0,00	-12,44	6,69E-02	+	*Pck2*	XM_006519633.2	=
2352,25	990,30	2017,53	808,29	464,83	646,53	-1,48	9,18E-02	-	*Pcmt1*	XM_006512595.3	=
5,26	1,77	18,52	99,87	155,94	131,80	3,94	1,06E-03	+	*Pcmtd2*	XM_006500620.3	=
1,05	1,77	3,27	609,60	0,00	211,06	7,08	3,67E-02	-	*Pde8b*	XM_006517590.3	=
0,00	0,00	0,00	33,29	16,99	16,03	6,90	1,10E-04	-	*Pdia5*	XR_384603.3	=
0,00	0,00	0,00	661,61	4619,27	0,00	26,38	4,52E-09	+	*Pdk1*	XR_001782095.1	=
4437,53	2,66	599,16	3,12	0,00	0,00	-10,67	3,57E-03	-	*Pdzd2*	XM_017316734.1	=
37,84	49,65	0,00	1085,00	515,81	456,85	4,55	9,45E-02	-	*Pdzd2*	XM_006520166.3	=
0,00	0,89	4,36	181,01	57,98	161,19	6,29	4,12E-06	-	*Pex5l*	NM_001310460.1	=
12656,75	98,41	12934,19	139,40	113,96	36,51	-6,47	1,33E-03	-	*Pfkl*	XM_017313846.1	=
27,33	50,53	59,92	334,97	191,93	1114,95	3,58	2,40E-03	-	*Pggt1b*	XR_001782356.1	=
441,44	25,71	289,77	18,72	26,99	19,59	-3,53	3,89E-02	+	*Phactr1*	XM_006516907.3	=
0,00	18067,47	13656,45	0,00	0,00	0,00	-29,51	2,21E-11	+	*Phb2*	NM_007531.2	=
434,08	207,46	5,45	5,20	11,00	4,45	-4,97	2,37E-02	+	*Phf12*	XM_017314564.1	=
50,45	25,71	38,13	75,94	86,97	133,58	1,38	8,27E-02	+	*Phf24*	XM_017320123.1	=
95,65	4,43	40,31	0,00	0,00	1,78	-6,24	3,84E-02	-	*Phldb1*	XM_017313076.1	=
1226,58	1682,72	1264,77	3802,17	2531,05	2564,75	1,09	1,88E-02	+	*Pias2*	NM_001164168.1	=
197,60	176,43	163,41	710,50	490,82	741,82	1,85	8,84E-10	+	*Pias2*	NM_001164170.1	=
35,74	8,87	10,89	176,85	169,94	227,98	3,38	8,52E-05	+	*Pias2*	XR_001782348.1	=
225,98	316,51	75,17	3016,77	1035,61	3956,65	3,70	6,18E-04	+	*Pias2*	NM_001164167.1	=
59,91	37,24	33,77	751,07	661,75	923,49	4,16	3,13E-29	+	*Pias2*	NM_001164169.1	=
24,17	18,62	15,25	811,41	636,76	938,63	5,36	1,29E-49	+	*Pias2*	NM_008602.4	=
982,73	1413,20	166,67	118,59	114,96	78,37	-3,04	2,48E-02	+	*Pias3*	NM_001165949.1	=
6,31	17,73	8,72	12,48	384,86	251,13	4,30	2,88E-02	+	*Pias3*	XM_006501393.1	=
0,00	0,00	4,36	265,27	309,88	6,23	7,08	3,80E-02	-	*Pidd1*	XM_006536221.1	=
27,33	17,73	27,23	0,00	0,00	0,00	-7,07	9,16E-06	-	*Piezo2*	XM_006526170.3	=
39,94	5,32	104,58	1,04	1,00	1,78	-5,28	9,50E-03	-	*Pign*	NM_013784.3	=
2,10	0,00	3,27	3,12	266,90	166,53	6,37	2,04E-02	+	*Pik3c2b*	XM_006529533.3	=
15,77	6,21	8,72	0,00	0,00	0,00	-5,83	7,63E-03	-	*Pilrb2*	XR_868611.1	=
108,26	6,21	116,56	1,04	1,00	0,89	-6,30	1,39E-03	-	*Pip4k2c*	XR_871695.2	=
10,51	7,09	18,52	924,80	793,70	11,58	5,58	5,22E-03	-	*Pip5k1a*	XR_375498.2	=
21,02	15,07	10,89	351,61	100,96	32,95	3,37	3,90E-02	+	*Pitpnm1*	NM_008851.4	=
44,14	39,90	136,17	87,38	1112,58	1152,35	3,42	8,34E-02	-	*Pitpnm2*	XM_017320744.1	=
486,64	156,04	165,59	3,12	3,00	1,78	-6,69	8,93E-12	+	*Pitrm1*	XM_006516509.3	=
25,23	23,05	34,86	56,17	121,95	141,60	1,95	1,29E-02	+	*Pitx2*	NM_001042502.2	=
0,00	0,00	0,00	244,46	142,95	25,83	9,54	2,88E-05	+	*Pkd1*	XM_017317332.1	=
0,00	0,00	0,00	237,18	181,93	50,76	9,73	2,79E-07	+	*Pkd1*	XM_017317333.1	=
1,05	0,89	4,36	40,57	18,99	43,64	4,08	5,27E-04	+	*Pkd2l2*	XM_017317944.1	=
79,88	26,60	72,99	12,48	12,00	11,58	-2,31	1,05E-02	-	*Pla2g6*	XM_017316699.1	=
77,78	38,12	75,17	530,54	335,87	105,97	2,35	7,52E-02	-	*Plcb3*	XM_006526766.3	=
42,04	99,30	86,06	0,00	0,00	0,89	-7,76	1,66E-06	+	*Plekha1*	XM_006507140.1	=
0,00	0,00	0,00	36,41	5,00	25,83	6,92	8,97E-03	+	*Plekha4*	NM_148927.2	=
176,58	225,19	269,08	378,66	973,64	740,93	1,64	3,75E-02	-	*Plekhm3*	NM_001039493.1	=
0,00	5,32	6,54	82,18	100,96	14,25	4,05	7,40E-02	-	*Plin5*	XM_006524805.1	=

Additional file1: Table S1. Differentially expressed transcripts (*continued*)

Control1	Control2	Control3	PB1	PB2	PB3	log2FoldChange	P.adj	Strand	Gene Name	Transcript ID	Class Code
210,21	214,55	101,31	54,09	49,98	78,37	-1,53	5,01E-02	+	*Plppr2*	XM_006510187.3	=
0,00	0,00	0,00	48,89	139,95	171,87	9,35	1,34E-07	-	*Pls3*	NM_001166454.2	=
0,00	0,00	0,00	251,74	947,65	712,43	11,75	5,44E-12	+	*Plxnb1*	XM_006512081.1	=
85,14	87,77	239,66	4,16	0,00	4,45	-5,57	1,22E-05	+	*Pnisr*	XR_001784182.1	=
1,05	19,50	413,96	1,04	1,00	0,89	-7,21	1,81E-02	+	*Pnpla6*	XM_006508827.3	=
446,70	324,49	371,48	66,58	92,97	78,37	-2,26	2,86E-15	+	*Pnpla7*	NM_146251.4	=
0,00	0,89	1,09	37,45	33,99	2,67	5,22	5,31E-02	+	*Pnpla7*	XR_001782552.1	=
0,00	0,00	0,00	1133,89	1451,46	225,31	12,31	2,21E-10	+	*Pnpla7*	XM_006498016.3	=
26,28	35,46	32,68	57,21	82,97	90,83	1,29	1,16E-02	+	*Poc1b*	XR_001779545.1	=
9,46	3,55	7,63	677,21	4,00	195,92	5,41	2,37E-02	-	*Polb*	XM_006509029.2	=
0,00	0,00	0,00	29,13	27,99	154,06	8,57	3,33E-04	+	*Polr3e*	XM_006507889.3	=
40,99	30,14	7,63	0,00	3,00	0,89	-4,34	3,05E-02	+	*Pomgnt1*	NM_001290658.1	=
0,00	0,00	0,00	5,20	5,00	12,47	5,36	3,71E-02	-	*Pou2f2*	XM_006539652.3	=
5,26	33,69	9,80	33,29	92,97	757,85	4,18	9,40E-02	+	*Ppara*	XM_011245516.2	=
0,00	0,00	0,00	322,48	803,70	3,56	10,99	2,10E-03	+	*Ppara*	XM_011245517.2	=
25,23	13,30	31,59	1,04	0,00	0,89	-5,17	1,75E-03	-	*Ppie*	XM_006503249.3	=
2,10	1,77	1,09	2,08	152,94	56,10	5,41	2,47E-02	-	*Ppip5k2*	XM_006529490.3	=
0,00	0,89	0,00	777,08	1098,59	0,00	10,76	3,02E-02	+	*Ppm1b*	XM_011246322.2	=
4,20	1,77	3,27	94,66	84,97	2,67	4,31	6,13E-02	+	*Ppm1f*	XM_006522508.3	=
37,84	64,72	33,77	2,08	0,00	0,00	-6,12	4,97E-05	-	*Ppox*	XM_011238773.2	=
0,00	0,00	0,00	67,62	1,00	37,40	7,58	7,21E-02	-	*Ppp1r12c*	XM_011250502.2	=
0,00	0,00	6,54	531,58	313,88	489,80	7,70	5,62E-05	+	*Ppp1r16b*	XM_011239492.2	=
8,41	1,77	17,43	58,25	74,97	103,30	3,12	1,52E-02	+	*Ppp1r35*	XM_006504615.3	=
88,29	0,89	17,43	1,04	1,00	0,89	-5,19	9,34E-02	+	*Ppp4r1*	NM_001114131.1	=
568,62	1055,91	720,08	300,64	275,90	364,23	-1,32	1,16E-02	-	*Ppp6r1*	XM_011250556.2	=
685,28	643,65	898,74	134,19	95,96	284,08	-2,11	7,90E-03	+	*Prex2*	NM_029525.1	=
17,87	0,00	6,54	606,47	668,75	362,45	6,08	2,87E-04	+	*Prex2*	XM_006495441.3	=
304,80	402,50	6,54	1,04	4,00	1,78	-6,71	8,32E-04	-	*Prkaa2*	XM_006502651.3	=
10,51	19,50	22,88	364,09	860,68	408,76	4,95	4,69E-14	-	*Prkg1*	NM_011160.3	=
1211,86	1452,21	752,76	550,30	517,81	531,65	-1,10	5,20E-02	-	*Prmt2*	NM_133182.3	=
483,48	493,82	21,79	21,85	20,99	19,59	-4,00	2,33E-02	+	*Prnp*	NM_001278256.1	=
11,56	8,87	8,72	0,00	1,00	0,00	-4,80	1,82E-02	-	*Prok2*	NM_015768.2	=
1131,98	9,75	1280,02	0,00	0,00	0,00	-12,14	1,54E-04	+	*Prosc*	XM_017312523.1	=
206,01	93,98	63,18	31,21	18,99	28,50	-2,21	2,58E-02	-	*Proser2*	XM_011238976.2	=
27,33	17,73	18,52	4,16	9,00	7,12	-1,64	8,24E-02	+	*Prpf39*	XM_006516031.2	=
0,00	0,89	0,00	66,58	105,96	25,83	7,52	5,43E-05	+	*Prpf4b*	XM_017315440.1	=
21,02	31,03	6,54	2,08	1,00	0,89	-3,91	2,12E-02	-	*Prrc2c*	XM_006496772.3	=
9,46	9,75	4,36	42,65	24,99	46,31	2,26	3,49E-03	-	*Prrg2*	XM_006541063.2	=
1,05	0,00	1,09	149,80	0,00	212,84	7,43	7,98E-02	-	*Prss36*	XM_017312390.1	=
157,66	162,24	190,64	58,25	100,96	57,88	-1,23	2,26E-02	-	*Prss53*	XM_017322318.1	=
0,00	0,00	0,00	110,27	56,98	128,24	9,06	7,46E-09	-	*Prss53*	NM_001081268.1	=
0,00	0,00	0,00	11,44	15,99	5,34	5,88	8,97E-03	+	*Psapl1*	NM_175249.3	=
66,22	55,85	13,07	2,08	0,00	0,00	-6,08	4,26E-03	+	*Psd2*	XM_017317995.1	=
4608,86	4220,97	4157,07	0,00	0,00	0,00	-14,56	7,81E-31	-	*Psme2*	XR_383145.2	=
1500,90	1823,68	1366,08	5479,08	5546,93	5612,16	1,83	2,50E-24	-	*Psme2*	NM_011190.3	=
1212,91	0,00	979,35	0,00	2,00	0,00	-10,11	6,44E-02	-	*Ptch1*	XM_006517160.3	=
3,15	0,89	3,27	177,89	1,00	92,62	5,23	5,91E-02	-	*Ptgfrn*	XM_017319503.1	=
180,78	1,77	236,40	0,00	4,00	2,67	-5,97	5,03E-02	-	*Pth1r*	XM_017313214.1	=
48,35	79,79	79,52	616,88	96,96	372,24	2,39	9,63E-02	-	*Pth1r*	XM_006511985.2	=
279,58	187,07	166,67	13,52	17,99	30,28	-3,35	7,39E-10	+	*Ptp4a3*	NM_001166388.1	=
135,59	0,00	1225,55	0,00	1,00	0,89	-9,48	2,49E-02	-	*Ptpn2*	XM_011246858.2	=
269,07	426,44	453,18	0,00	0,00	0,00	-11,06	3,47E-15	+	*Pum1*	XM_006539305.3	=
309,01	261,54	256,00	0,00	0,00	0,00	-10,59	1,83E-15	+	*Pum1*	XM_006539304.3	=
304,80	257,11	257,09	0,00	0,00	0,00	-10,57	1,84E-15	+	*Pum1*	XM_006539306.3	=
59,91	15,96	15,25	0,00	0,00	0,00	-7,40	1,19E-03	+	*Pum1*	XM_006539303.3	=
81,98	104,62	47,93	1785,10	1819,32	105,08	3,98	1,69E-02	+	*Pum2*	XM_006515277.2	=
0,00	0,00	0,00	104,03	74,97	124,68	9,10	3,09E-10	+	*Purg*	XM_017312994.1	=
0,00	5,32	4,36	218,46	77,97	109,54	5,37	9,83E-06	+	*Pus10*	XM_006514870.1	=
81,98	86,88	132,90	184,13	217,92	211,06	1,03	2,91E-02	+	*Pwwp2a*	XM_006534205.3	=
33,63	66,49	50,11	1582,24	1117,58	1526,38	4,81	1,93E-34	-	*R3hcc1*	NM_001146012.2	=
2347,00	0,00	2633,03	0,00	0,00	0,00	-13,18	4,01E-02	+	*R3hdm2*	XM_006514166.2	=
339,49	341,33	308,29	803,09	1123,58	889,65	1,51	2,18E-10	+	*Rab30*	XM_006508294.2	=
71,47	46,99	68,63	6,24	8,00	13,36	-2,74	1,60E-06	+	*Rab3il1*	XM_011247385.2	=
7465,61	8380,77	7717,16	1737,24	1450,46	1126,53	-2,45	5,73E-22	-	*Racgap1*	NM_001253808.1	=
389,94	159,58	124,19	867,58	612,77	886,97	1,81	8,26E-02	-	*Racgap1*	XM_011245647.2	=
191,29	247,35	202,62	1044,43	695,74	1049,94	2,12	4,06E-11	-	*Racgap1*	NM_012025.7	=
315,32	229,62	166,67	4256,77	4076,48	4887,26	4,22	1,15E-34	-	*Racgap1*	XM_006521019.2	=
13,66	39,90	6,54	830,13	1097,59	409,65	5,27	1,25E-07	-	*Rai14*	XM_006520188.3	=
1,05	0,89	0,00	9,36	387,86	260,93	8,38	2,69E-04	-	*Rai14*	XM_017316775.1	=
0,00	0,89	0,00	0,00	818,69	311,69	10,03	4,83E-02	-	*Rai14*	NM_030690.3	=
69,37	84,22	53,38	134,19	142,95	148,72	1,04	4,98E-03	+	*Rai2*	NM_198409.3	=
0,00	0,00	0,00	421,31	65,98	179,00	10,23	4,70E-07	-	*Ralgps1*	XM_006498033.2	=
4,20	2,66	7,63	408,82	2,00	151,39	5,28	3,89E-02	+	*Raly*	XM_017316631.1	=
28,38	437,97	43,58	0,00	0,00	0,00	-9,89	7,23E-04	-	*Ranbp17*	NR_110973.1	=
3447,45	5,32	2935,88	53,05	48,98	38,29	-5,51	3,83E-02	-	*Rangap1*	XR_001781504.1	=
0,00	0,00	0,00	1404,36	0,00	568,16	11,80	9,87E-02	-	*Rasa2*	XM_006510776.3	=
160,81	3,55	19,61	0,00	0,00	0,00	-8,42	2,75E-02	+	*Rasal1*	XM_017320735.1	=
0,00	937,11	1808,37	0,00	0,00	0,00	-12,32	7,23E-02	-	*Rassf2*	XM_006499141.2	=
186,04	214,55	15,25	3,12	4,00	13,36	-4,34	1,75E-02	-	*Rassf5*	NM_001311094.2	=
901,80	1422,06	940,13	304,80	233,91	436,36	-1,74	1,16E-03	+	*Rassf7*	NM_025886.3	=
1,05	1,77	3,27	315,20	5,00	83,71	6,06	5,30E-03	+	*Rbfox1*	NM_021477.5	=
29,43	7,09	11,98	96,74	267,90	78,37	3,20	1,27E-02	-	*Recql*	NM_001204907.1	=
346,85	440,63	443,38	228,86	156,94	211,06	-1,04	3,89E-03	+	*Repin1*	NM_175099.3	=
16,82	118,80	26,15	7,28	6,00	7,12	-2,99	8,25E-02	+	*Retnlg*	NM_181596.4	=
0,00	0,00	0,00	788,52	2001,25	4084,89	13,60	4,13E-14	-	*Rev1*	XM_006496161.2	=

Additional file1: Table S1. Differentially expressed transcripts (*continued*)

Control1	Control2	Control3	PB1	PB2	PB3	log2FoldChange	P.adj	Strand	Gene Name	Transcript ID	Class Code
263,81	381,23	313,74	3142,64	3128,83	325,05	2,78	9,45E-02	+	*Rfx1*	NM_009055.4	=
1,05	0,00	1,09	84,26	64,98	64,12	6,73	1,07E-07	-	*Rgl1*	XM_017319386.1	=
140,84	274,84	2,18	4,16	3,00	1,78	-5,55	1,71E-02	+	*Rhag*	XM_006523912.2	=
35,74	31,92	34,86	6,24	12,00	15,14	-1,60	1,55E-02	-	*Rhno1*	XM_017321764.1	=
29,43	31,03	27,23	0,00	0,00	0,00	-7,35	7,32E-07	+	*Rhox4d*	NM_001039695.1	=
75,68	120,57	143,80	2417,58	2429,09	186,12	3,89	9,52E-03	+	*Riok1*	NM_024242.3	=
301,65	311,19	16,34	19,77	11,00	27,61	-3,43	9,68E-02	-	*Ripk2*	XR_390315.3	=
94,59	120,57	126,37	246,54	296,89	443,49	1,53	1,47E-03	-	*Rmnd1*	XM_017314053.1	=
88,29	79,79	112,21	306,88	497,81	218,18	1,87	2,61E-03	-	*Rmnd1*	XM_017314055.1	=
9,46	3,55	9,80	381,78	7,00	314,36	4,95	1,76E-02	+	*Rnf103*	NM_001308303.1	=
105,11	72,70	87,15	48,89	40,98	35,62	-1,08	1,96E-02	+	*Rnf114*	NM_030743.5	=
0,00	0,00	0,00	1403,32	0,00	1198,66	12,20	7,75E-02	-	*Rnf141*	XM_006508124.2	=
2,10	157,81	82,79	2,08	2,00	1,78	-5,37	1,84E-02	-	*Rnf146*	NM_001284279.1	=
76,73	44,33	51,20	141,48	199,93	255,58	1,80	1,41E-03	+	*Rnf32*	NM_021470.6	=
0,00	0,00	0,00	92,58	80,97	1,78	8,30	2,38E-02	-	*Rnf38*	XM_006538299.3	=
0,00	0,00	0,00	468,12	1391,48	1,78	11,71	2,83E-03	-	*Rnf38*	XM_006538301.3	=
0,00	0,00	0,00	0,00	2182,18	1431,98	12,67	5,74E-02	+	*Rnf40*	XM_006507751.3	=
274,32	44,33	63,18	2411,34	8572,80	240,44	4,88	2,21E-02	-	*Rnf44*	XM_011244449.1	=
1592,34	130,33	1496,81	0,00	0,00	0,00	-12,55	2,23E-08	-	*Robo2*	XM_006523036.3	=
0,00	0,00	0,00	30,17	19,99	61,45	7,65	4,20E-05	+	*Rpain*	NM_001252415.1	=
2,10	0,89	1,09	0,00	176,93	112,21	6,15	7,61E-02	-	*Rph3al*	XM_006533642.3	=
37,84	23,94	14,16	1,04	0,00	0,00	-6,18	9,20E-04	+	*Rpl3l*	XM_011246569.2	=
0,00	0,00	0,00	81,14	1,00	41,86	7,80	6,36E-02	-	*Rpp40*	XM_006516631.3	=
11,56	70,04	37,04	0,00	0,00	0,00	-7,79	6,10E-04	-	*Rps6ka4*	XM_017318261.1	=
186,04	212,78	15,25	0,00	0,00	0,00	-9,59	1,63E-04	-	*Rps6kb2*	XM_006531813.3	=
148,20	0,89	44,66	0,00	1,00	0,00	-7,53	4,25E-02	-	*Rps6kb2*	XM_006531814.3	=
15,77	20,39	14,16	144,60	173,94	19,59	2,75	9,75E-02	-	*Rras2*	XM_017312239.1	=
37,84	30,14	38,13	151,88	846,68	691,95	3,99	1,95E-05	-	*Rsu1*	XM_006497405.3	=
166,07	140,08	96,95	236,14	253,91	328,61	1,02	7,59E-02	-	*Runx2*	NM_001145920.2	=
0,00	0,00	0,00	1977,54	1767,34	0,00	12,72	5,56E-02	-	*Ryr2*	XM_017315447.1	=
62,01	11,53	104,58	1,04	0,00	0,00	-7,41	1,09E-03	+	*S100a14*	NM_001163525.2	=
8,41	5,32	4,36	8,32	50,98	669,68	5,33	2,83E-02	-	*S100pbp*	XM_006503448.2	=
16,82	27,48	4,36	33,29	119,96	2027,75	5,48	2,75E-02	-	*S100pbp*	XM_006503450.3	=
700,00	616,17	676,50	0,00	8,00	0,00	-7,97	9,92E-05	-	*Samd12*	XM_006521080.3	=
0,00	21,28	0,00	699,06	777,71	775,66	6,72	3,95E-02	-	*Samd12*	XM_006521085.3	=
4,20	1,77	7,63	13,52	105,96	22,26	3,39	9,74E-02	-	*Sat1*	NM_001291865.1	=
13,66	7,09	16,34	15,60	711,73	803,26	5,37	3,96E-03	-	*Sbf2*	XM_011241830.2	=
1,05	0,89	1,09	72,82	595,78	627,83	8,75	5,72E-09	-	*Sbno1*	NM_001081203.1	=
3,15	0,00	2,18	3,12	51,98	184,34	5,51	6,44E-02	-	*Sclt1*	XR_867171.2	=
4510,06	707,49	1085,02	384,90	238,91	411,43	-2,61	9,87E-02	-	*Scube1*	NM_001271472.1	=
0,00	7,09	10,89	3084,39	2732,98	7,12	8,34	3,51E-03	-	*Scube1*	XM_006521243.2	=
2105,25	2380,45	1854,12	22,89	14,99	51,65	-6,14	3,43E-24	-	*Scyl2*	XM_011243394.2	=
3404,35	144,51	2831,30	4,16	2,00	4,45	-9,23	7,02E-11	+	*Sec23ip*	XM_006507519.3	=
0,00	0,89	2,18	12,48	11,00	105,97	5,42	3,15E-02	-	*Sec24b*	XM_006502467.3	=
3,15	4,43	58,83	652,25	655,76	326,83	4,63	1,50E-02	+	*Sec24d*	XM_006502038.3	=
4096,99	0,00	601,34	0,00	0,00	0,00	-13,09	4,25E-02	-	*Secisbp2l*	XM_006500156.3	=
90,39	13,30	103,49	0,00	0,00	0,00	-8,59	2,50E-04	-	*Sema4a*	XM_006501189.1	=
853,45	1009,81	1562,17	337,05	594,78	319,70	-1,45	5,98E-02	+	*Sema4g*	XR_386477.1	=
17,87	19,50	21,79	4,16	3,00	0,89	-2,91	1,28E-03	+	*Sema5a*	XM_011245340.1	=
0,00	0,00	0,00	46,81	23,99	34,73	7,57	2,26E-06	+	*Sema6d*	XM_011239427.2	=
7261,71	6245,02	7355,49	406,74	1394,48	1642,15	-2,60	3,02E-03	-	*Senp3*	XM_006534530.2	=
272,22	469,00	347,51	10458,83	1847,31	1967,20	3,71	2,42E-03	-	*Senp3*	NM_030702.4	=
63,06	87,77	132,90	300,64	644,76	367,79	2,21	1,78E-03	-	*Senp5*	XM_006522243.3	=
0,00	0,00	0,00	21,85	172,94	545,01	10,38	1,40E-04	+	*Sept11*	XM_006535106.3	=
0,00	0,00	0,00	3,12	78,97	790,80	10,62	7,80E-03	+	*Sept11*	NM_001310669.1	=
0,00	0,00	0,00	76,98	59,98	77,48	8,60	1,02E-09	+	*Sept3*	XM_017316608.1	=
1609,16	1313,90	1449,96	0,00	0,00	11,58	-8,55	1,74E-04	-	*Sept5*	NM_213614.2	=
151,35	800,58	421,59	2,08	3,00	0,00	-8,11	1,58E-09	+	*Sept9*	XM_006533746.3	=
0,00	0,00	0,00	138,36	61,98	30,28	8,70	1,09E-05	-	*Serac1*	XM_006523234.3	=
0,00	0,00	0,00	10,40	8,00	12,47	5,80	1,82E-03	+	*Serpina3i*	XM_017315152.1	=
0,00	0,00	0,00	21,85	23,99	11,58	6,69	1,63E-04	+	*Serpinb1b*	XM_006516685.2	=
1793,09	1562,14	2182,03	1009,06	443,83	781,89	-1,31	8,07E-02	-	*Serpinh1*	NM_001111043.1	=
0,00	0,89	1,09	80,10	0,00	465,75	8,11	6,18E-02	+	*Setd6*	XR_387770.2	=
0,00	0,00	0,00	221,58	351,87	366,01	10,73	2,65E-14	-	*Sez6l*	NM_019982.3	=
118,77	168,45	189,55	294,40	2609,03	3520,29	3,75	6,59E-03	-	*Sf3b3*	XM_017312494.1	=
597,00	529,28	209,16	5116,03	4531,31	626,05	2,94	7,38E-02	+	*Sfmbt1*	XM_011245120.2	=
0,00	0,00	0,00	36,41	165,94	98,85	9,09	2,05E-06	+	*Sfswap*	XR_377056.2	=
15,77	119,69	2,18	0,00	0,00	0,00	-8,00	5,31E-02	-	*Sgsm2*	XR_001780084.1	=
17,87	115,25	1,09	0,00	0,00	0,00	-7,96	8,25E-02	-	*Sgsm2*	XR_001780083.1	=
367,87	446,83	3,27	0,00	1,00	2,67	-7,78	2,99E-03	+	*Sh3d19*	XM_017319605.1	=
66,22	89,54	64,27	875,90	301,89	242,23	2,69	4,36E-03	+	*Sh3d19*	XM_006501547.3	=
3154,20	695,07	1875,91	59,30	53,98	75,70	-4,92	1,05E-10	+	*Sh3kbp1*	NM_001135727.2	=
255,41	35,46	137,26	2875,29	2648,01	3011,79	4,32	6,07E-06	+	*Sh3kbp1*	XM_017318585.1	=
176,58	13,30	28,32	3,12	5,00	2,67	-4,34	2,09E-02	-	*Shkbp1*	XM_006539668.2	=
1,05	19,50	192,82	1,04	1,00	2,67	-5,49	7,93E-02	-	*Sik2*	XM_006510267.2	=
4050,75	2409,71	0,00	0,00	0,00	0,00	-13,55	3,07E-02	+	*Sik3*	XM_006510590.2	=
1,05	0,00	0,00	289,19	1108,59	0,00	10,34	4,58E-02	-	*Sipa1l2*	XM_006531024.1	=
0,00	0,00	0,00	163,32	184,93	84,60	9,61	3,93E-10	+	*Slc10a7*	XM_006531488.1	=
5,26	6,21	4,36	2,08	276,90	173,65	4,84	4,65E-02	-	*Slc11a2*	XM_006520578.3	=
18,92	1,77	1,09	82,18	128,95	99,74	3,84	4,29E-02	-	*Slc25a23*	XM_011246576.2	=
11,56	3,55	4,36	544,06	7,00	82,82	5,03	3,93E-02	+	*Slc25a40*	XM_006503591.3	=
3,15	5,32	6,54	0,00	0,00	0,00	-4,80	7,59E-02	-	*Slc30a7*	XM_017319686.1	=
465,62	383,00	360,58	0,00	0,00	0,00	-11,13	6,15E-17	-	*Slc38a10*	XM_017314781.1	=
264,86	242,03	105,67	0,00	0,00	0,00	-10,15	1,49E-10	-	*Slc38a10*	XM_006534285.3	=
244,89	70,93	201,54	0,00	0,00	0,00	-9,91	1,03E-08	-	*Slc38a10*	XM_006534287.3	=

Additional file1: Table S1. Differentially expressed transcripts (*continued*)

Control1	Control2	Control3	PB1	PB2	PB3	log2FoldChange	P.adj	Strand	Gene Name	Transcript ID	Class Code
322,67	255,33	241,84	116,51	121,95	105,08	-1,26	1,40E-06	-	*Slc38a10*	XM_017314785.1	=
425,68	212,78	164,50	2440,46	521,81	2426,71	2,75	3,30E-02	-	*Slc38a10*	XM_006534290.2	=
73,57	30,14	33,77	5053,61	5282,03	4283,48	6,74	9,85E-44	-	*Slc38a10*	XM_006534291.2	=
5,26	5,32	8,72	160,20	1683,37	317,03	6,81	9,18E-08	-	*Slc38a7*	XM_011248357.1	=
1047,90	38,12	383,46	43,69	25,99	39,18	-3,75	5,24E-02	-	*Slc39a14*	XM_006518789.3	=
16,82	6,21	17,43	0,00	0,00	0,00	-6,23	3,51E-03	+	*Slc44a4*	NM_023557.3	=
0,00	0,00	0,00	184,13	108,96	6,23	9,08	2,53E-03	+	*Slc4a7*	XM_011244756.2	=
0,00	0,00	0,00	3,12	11,00	14,25	5,68	4,24E-02	+	*Slc6a4*	XM_006532300.2	=
62,01	69,15	17,43	12,48	9,00	6,23	-2,43	5,36E-02	+	*Slc7a10*	NM_017394.4	=
36,79	143,62	33,77	0,00	0,00	0,00	-8,64	4,77E-05	-	*Slc7a4*	XR_384549.3	=
0,00	0,89	0,00	1,04	99,96	64,12	7,26	3,95E-02	-	*Slc7a7*	XM_006518748.2	=
9,46	220,76	70,81	8,32	8,00	2,67	-3,99	5,59E-02	+	*Slc9a8*	XM_006500395.3	=
3,15	0,00	0,00	34,33	16,99	14,25	4,42	3,64E-02	-	*Slf1*	XM_011244457.2	=
3,15	30,14	31,59	0,00	0,00	0,89	-5,95	3,41E-02	+	*Slfnl1*	XM_017320083.1	=
565,47	318,28	437,93	14275,56	274,90	15083,91	4,49	2,81E-02	+	*Slit2*	XM_006503816.3	=
3,15	0,00	0,00	142,52	5,00	241,34	6,96	3,24E-02	-	*Slitrk4*	XM_017318485.1	=
233,33	265,09	259,27	3451,60	264,90	2701,00	3,08	6,16E-02	+	*Smarcc2*	NM_001114097.1	=
447,75	461,02	579,55	23,93	3,00	3,56	-5,62	3,92E-07	-	*Smarcd2*	XM_006534552.3	=
0,00	0,00	0,00	281,91	179,93	260,93	10,35	1,26E-13	+	*Smg6*	XM_011248660.1	=
2493,09	0,00	2492,50	0,00	0,00	0,00	-13,18	4,01E-02	-	*Smtn*	XM_017314586.1	=
13,66	7,98	8,72	19,77	154,94	294,77	3,95	1,22E-02	-	*Smyd3*	XM_011238864.2	=
14,71	15,96	10,89	1,04	1,00	0,89	-3,84	5,51E-03	-	*Snapc4*	XM_011239068.1	=
148,20	168,45	283,24	2248,01	1796,33	4485,63	3,83	1,27E-09	+	*Snx13*	NM_001014973.2	=
152,40	97,52	131,81	56,17	58,98	65,90	-1,07	1,42E-02	-	*Sohlh1*	XM_006497875.3	=
72,52	68,27	82,79	35,37	12,00	18,70	-1,76	2,57E-02	+	*Sorbs2*	XR_001778428.1	=
135,59	86,00	220,05	0,00	0,00	0,00	-9,68	1,11E-09	-	*Sox17*	XM_006495474.2	=
0,00	0,00	0,00	322,48	14,99	1,78	9,26	4,56E-02	-	*Sox17*	XM_011238364.2	=
60,96	59,40	69,72	22,89	34,99	35,62	-1,02	4,13E-02	+	*Spata18*	XM_006504155.2	=
72,52	39,01	107,85	0,00	0,00	0,00	-8,67	2,31E-07	-	*Spata2l*	XM_006531505.3	=
0,00	0,00	0,00	4,16	8,00	4,45	4,90	5,80E-02	+	*Spata3*	NM_027300.3	=
1281,23	937,99	1108,99	0,00	0,00	0,00	-12,60	9,80E-22	+	*Spats2l*	XM_006496218.3	=
0,00	1,77	0,00	8,32	99,96	94,40	6,74	7,51E-03	+	*Specc1l*	XM_011243589.2	=
38,89	26,60	88,24	0,00	0,00	0,00	-8,16	1,31E-05	+	*Sphk1*	XM_006532672.3	=
0,00	0,00	0,00	6,24	3,00	6,23	4,80	7,93E-02	-	*Sprr1b*	NM_009265.3	=
12,61	203,91	141,62	12,48	14,99	3,56	-3,53	8,81E-02	+	*Srd5a3*	XM_006535162.1	=
0,00	0,89	1,09	1,04	33,99	64,12	5,64	8,26E-02	-	*Srek1*	XM_006517608.3	=
3,15	0,89	0,00	6,24	34,99	28,50	4,12	5,89E-02	+	*Ssbp2*	XM_011244554.1	=
4,20	2,66	6,54	244,46	4,00	260,93	5,25	1,40E-02	-	*Sstr3*	XM_006520669.3	=
750,45	2,66	336,62	3,12	11,00	2,67	-6,02	1,65E-02	-	*Stard13*	XM_006504921.2	=
39,94	26,60	38,13	8,32	6,00	5,34	-2,42	8,68E-05	-	*Stau2*	XM_011238387.1	=
1556,61	1940,71	0,00	1,04	1,00	0,89	-10,22	9,27E-04	+	*Stim1*	XM_017322078.1	=
1,05	1,77	3,27	673,05	0,00	711,54	7,83	1,45E-02	-	*Stk17b*	XM_006496359.3	=
4,20	33,69	107,85	1,04	0,00	0,89	-6,23	1,70E-02	-	*Stk32c*	XM_006536216.3	=
4605,70	778,41	972,82	0,00	4,00	0,00	-10,65	5,51E-08	-	*Stk38*	XM_011246233.2	=
357,36	359,06	489,13	2,08	2,00	1,78	-7,69	2,86E-27	-	*Stk38*	XM_011246232.2	=
8,41	23,94	11,98	8,32	359,87	352,65	4,02	8,54E-02	-	*Stox2*	XM_017312966.1	=
779,88	5265,36	3943,55	868,62	268,90	349,09	-2,75	9,94E-02	-	*Strbp*	XM_011239046.2	=
73,57	315,62	71,90	17,68	9,00	2,67	-3,98	8,24E-03	+	*Stx16*	XM_006499437.3	=
966,97	385,66	50,11	8,32	13,00	8,91	-5,54	7,89E-05	-	*Suco*	XM_006496759.2	=
2,10	0,00	1,09	1,04	95,96	292,10	6,95	2,87E-02	+	*Sumo3*	NR_125903.1	=
73,57	39,01	63,18	379,70	468,82	82,82	2,41	8,52E-02	+	*Sun1*	XM_006504762.2	=
0,00	0,00	0,00	139,40	85,97	101,52	9,20	1,58E-10	+	*Synj2*	XM_006523208.3	=
0,00	5,32	2,18	95,70	42,98	44,53	4,57	5,20E-04	+	*Synpr*	NM_001163032.1	=
14922,81	16135,63	15017,09	6837,67	8048,99	7525,03	-1,04	5,82E-17	-	*Tab2*	NM_138667.3	=
264,86	108,16	202,62	6851,19	6801,46	5896,24	5,09	1,49E-29	-	*Tab2*	XM_011243203.2	=
124,02	0,00	101,31	2647,48	2400,10	2817,66	5,13	8,74E-02	-	*Tab2*	XM_011243204.2	=
1,05	0,89	0,00	13,52	13,99	13,36	4,35	8,24E-03	+	*Taf12*	XM_006539107.3	=
732,58	590,46	667,79	5191,97	853,68	4637,02	2,42	7,00E-02	+	*Tatdn2*	XM_006506335.2	=
0,00	1,77	0,00	0,00	1575,41	1235,17	10,59	4,03E-02	-	*Tbc1d8*	XM_017321765.1	=
0,00	0,00	0,00	384,90	73,97	70,35	9,90	7,45E-06	+	*Tbck*	XR_001783695.1	=
59,91	10,64	13,07	143,56	220,92	205,71	2,77	4,53E-02	-	*Tbk1*	XR_001779563.1	=
1,05	0,00	0,00	109,23	10,00	2,67	6,82	6,60E-02	-	*Tc2n*	NM_028924.3	=
2,10	31,92	147,07	0,00	0,00	0,89	-7,43	2,83E-02	-	*Tcp11*	XM_006524014.3	=
2379,58	66,49	2402,08	60,34	106,96	151,39	-3,93	6,87E-02	+	*Tdg*	XM_006513577.3	=
266,97	110,82	181,93	0,00	0,00	0,00	-10,02	1,03E-10	+	*Tdrd9*	XM_006516317.3	=
3,15	4,43	2,18	0,00	567,79	691,95	7,01	2,99E-02	-	*Tdrp*	XM_011242085.2	=
0,00	0,89	1,09	1,04	980,63	367,79	9,41	1,73E-03	-	*Tdrp*	XM_017312974.1	=
7,36	9,75	11,98	789,56	630,76	921,71	6,33	5,68E-53	-	*Tefm*	NM_183275.2	=
3,15	2,66	5,45	50,97	17,99	84,60	3,78	1,59E-03	+	*Tex15*	XM_011242106.2	=
24,17	12,41	16,34	8914,04	23,99	7047,71	8,24	2,84E-05	+	*Tfdp1*	XM_006508758.3	=
1,05	0,00	2,18	745,87	783,71	0,89	8,91	3,02E-03	-	*Tfg*	XM_006521983.3	=
4,20	0,89	3,27	106,11	123,95	2,67	4,81	4,47E-02	-	*Thnsl2*	NM_178413.5	=
294,29	214,55	69,72	996,57	1173,56	613,58	2,27	4,13E-02	-	*Thoc6*	XM_006524544.2	=
110,36	69,15	93,69	260,07	169,94	180,78	1,16	3,73E-02	+	*Thsd1*	NM_019576.2	=
74,62	204,80	45,75	0,00	0,00	0,00	-9,24	2,54E-06	+	*Tia1*	XM_011241285.2	=
2201,95	2722,67	2089,43	159,16	251,91	197,70	-3,53	1,35E-33	-	*Tiprl*	XM_006496779.3	=
1431,53	1344,93	1619,91	3455,76	3461,71	3560,36	1,25	6,28E-23	-	*Tiprl*	NM_145513.4	=
46,25	44,33	87,15	0,00	0,00	0,00	-8,37	1,44E-07	+	*Tle2*	XM_011243429.2	=
43,09	38,12	37,04	0,00	1,00	0,00	-6,82	5,52E-06	+	*Tle2*	XM_011243428.2	=
35,74	30,14	30,50	0,00	1,00	0,00	-6,52	2,27E-05	+	*Tle2*	XM_011243427.2	=
35,74	30,14	1,09	1,04	0,00	0,00	-6,00	9,52E-02	+	*Tle2*	XM_011243430.2	=
42,04	35,46	29,41	3,12	0,00	0,00	-5,18	3,90E-05	+	*Tle2*	XM_006513584.2	=
37,84	31,03	33,77	3,12	0,00	0,00	-5,12	1,59E-05	+	*Tle2*	XM_011243424.2	=
44,14	36,35	40,31	3,12	0,00	8,91	-3,31	5,66E-02	+	*Tle2*	XM_011243437.1	=
21,02	42,56	68,63	366,17	220,92	351,76	2,83	9,19E-05	+	*Tle3*	XM_006511028.3	=

Additional file1: Table S1. Differentially expressed transcripts (*continued*)

Control1	Control2	Control3	PB1	PB2	PB3	log2FoldChange	P.adj	Strand	Gene Name	Transcript ID	Class Code
9,46	14,19	9,80	13,52	659,75	73,02	4,48	5,56E-02	-	*Tln2*	XM_006511433.3	=
130,33	85,11	39,22	12,48	18,99	3,56	-2,87	3,22E-02	-	*Tm9sf1*	NM_028780.3	=
0,00	0,00	0,00	10,40	3,00	5,34	5,08	8,93E-02	+	*Tmc2*	XM_017316469.1	=
24,17	13,30	13,07	209,09	140,95	219,07	3,50	3,47E-13	-	*Tmc6*	XM_006533112.3	=
6568,01	6545,57	115,47	108,19	164,94	124,68	-5,06	7,26E-03	-	*Tmem131*	XM_017321814.1	=
7228,07	7720,28	537,06	585,67	453,83	654,54	-3,19	6,81E-02	-	*Tmem131*	XM_006496150.2	=
91,44	55,85	94,78	20,81	8,00	24,04	-2,19	7,26E-03	-	*Tmem131*	XM_006496152.2	=
11,56	31,92	7,63	0,00	0,00	0,00	-6,57	6,56E-03	+	*Tmem139*	XM_006505327.3	=
70,42	36,35	3,27	2025,40	22,99	1905,75	5,17	5,98E-02	+	*Tmem161a*	XM_006509642.1	=
2562,46	196,82	2267,00	47,85	50,98	0,00	-5,67	6,30E-02	-	*Tmem168*	XM_006504952.3	=
982,73	1088,71	848,63	506,61	313,88	174,55	-1,55	7,87E-02	-	*Tmem184b*	XM_006520806.2	=
116,67	0,89	168,85	1,04	3,00	0,00	-6,16	6,57E-02	-	*Tmem200a*	XM_006512919.3	=
14,71	0,00	15,25	1844,39	12,00	683,04	6,41	5,00E-02	-	*Tmem209*	NM_001311093.1	=
15,77	50,53	9,80	1,04	3,00	4,45	-3,15	8,93E-02	-	*Tmem25*	XM_011242603.1	=
1552,40	1638,39	657,98	174,76	330,88	221,74	-2,40	8,89E-04	-	*Tmem255a*	NM_172930.4	=
0,00	0,00	0,00	237,18	311,88	423,90	10,78	3,24E-14	-	*Tmem56*	XM_006502478.2	=
69,37	44,33	115,47	9,36	18,99	11,58	-2,52	2,47E-03	+	*Tmem63a*	XR_001783670.1	=
1,05	0,89	1,09	65,54	239,91	8,91	6,70	9,96E-04	-	*Tmem65*	XM_006521504.3	=
0,00	0,00	3,27	57,21	34,99	16,03	5,11	1,71E-02	-	*Tmem82*	XR_390735.1	=
0,00	0,00	0,00	223,66	124,95	5,34	9,32	3,01E-03	+	*Tmem8b*	NM_001085508.2	=
232,28	0,89	216,79	0,00	0,00	2,67	-7,36	6,58E-02	-	*Tmod2*	XM_006511267.3	=
1072,07	0,00	1150,38	0,00	0,00	0,00	-12,01	8,69E-02	+	*Tmprss13*	NM_001013373.2	=
6,31	1297,06	1379,15	20,81	7,00	5,34	-6,34	5,30E-03	-	*Tmtc3*	XM_011243449.2	=
0,00	0,00	0,00	22,89	17,99	203,04	8,78	2,81E-03	-	*Tmtc4*	XM_006519510.3	=
981,68	693,30	525,08	1821,51	1763,34	1963,63	1,33	2,87E-03	+	*Tnk2*	XM_006522336.3	=
0,00	0,00	1,09	65,54	81,97	5,34	7,14	6,40E-03	-	*Tnnt1*	XM_017322113.1	=
311,11	122,35	276,70	2711,97	425,84	6992,49	3,83	2,07E-02	+	*Tnpo2*	XM_011248342.2	=
10,51	6,21	7,63	367,21	151,94	165,64	4,82	7,33E-12	-	*Tnpo3*	NM_177296.4	=
8,41	701,28	1063,23	8,32	9,00	3,56	-6,41	1,41E-03	-	*Tnrc18*	XM_011240977.2	=
710,51	4,43	1042,54	2,08	1,00	2,67	-8,25	1,12E-04	+	*Tnrc6a*	XM_006507705.1	=
818,77	33,69	2186,38	35,37	32,99	46,31	-4,73	1,76E-02	+	*Tnrc6a*	XM_017322204.1	=
669,52	57,63	819,21	48,89	6,00	34,73	-4,11	3,64E-02	+	*Tnrc6a*	XM_017322201.1	=
60,96	78,90	65,36	28,09	13,99	17,81	-1,78	4,88E-04	+	*Tns2*	XM_011245537.2	=
5,26	9,75	6,54	425,47	217,92	386,49	5,56	2,21E-21	+	*Tns2*	XM_006520693.2	=
414,11	628,58	649,27	213,25	284,89	162,97	-1,36	1,56E-02	-	*Tomm34*	NM_001291155.1	=
130,33	172,88	294,13	0,00	0,00	0,00	-10,12	4,49E-11	-	*Tox*	NM_145711.4	=
28,38	33,69	16,34	158,12	195,93	195,92	2,80	3,18E-12	-	*Tox*	XM_006537944.3	=
887,09	812,10	884,58	1416,84	1923,28	3302,11	1,36	5,66E-02	+	*Tpm3*	NM_001271764.1	=
50,45	131,21	53,38	0,00	0,00	0,00	-8,77	4,21E-07	+	*Tpst2*	NM_001326590.1	=
105,11	160,47	212,43	17,68	21,99	15,14	-3,13	9,07E-10	-	*Traf1*	NM_009421.4	=
46,25	83,34	86,06	7,28	0,00	0,00	-4,91	9,94E-02	-	*Trappc9*	NM_001164643.1	=
18,92	11,53	16,34	48,89	93,96	157,62	2,69	1,62E-03	-	*Trdmt1*	XM_006497325.3	=
0,00	0,00	0,00	61,38	218,92	47,20	9,21	8,16E-06	-	*Trim14*	XM_017320421.1	=
38,89	39,01	51,20	1,04	0,00	0,00	-6,94	4,34E-06	+	*Trim17*	XM_011249157.2	=
0,00	0,00	0,00	50,97	5,00	38,29	7,41	7,56E-03	+	*Trim17*	NM_031172.2	=
0,00	0,00	0,00	205,97	486,82	455,06	11,02	7,96E-13	-	*Trim2*	NM_030706.3	=
948,05	880,37	1110,08	2983,48	4164,44	3359,99	1,84	1,63E-13	+	*Trim24*	NM_145076.4	=
1000,60	978,78	1010,94	4,16	4,00	3,56	-8,00	3,65E-69	+	*Trim33*	XR_375607.3	=
7,36	26,60	18,52	0,00	0,00	2,67	-4,23	8,58E-02	-	*Trim46*	XM_011240152.2	=
23,12	100,18	105,67	8,32	3,00	0,00	-4,35	4,05E-02	-	*Trmt11*	XM_006512881.3	=
1294,89	1111,76	1366,08	298,56	651,76	265,38	-1,63	3,76E-02	-	*Trmt5*	NM_029580.3	=
1654,35	1104,67	1192,87	3057,34	3097,84	1967,20	1,04	7,68E-02	-	*Trmt6*	NM_175113.3	=
0,00	0,00	0,00	1,04	33,99	395,40	9,60	3,89E-02	-	*Trp53bp1*	XM_017318936.1	=
220,72	208,34	343,15	85,30	62,98	62,34	-1,87	1,50E-05	-	*Trub2*	NR_110962.1	=
14,71	7,09	4,36	249,66	393,85	28,50	4,68	2,20E-03	+	*Tsc1*	NM_001289575.1	=
69,37	49,65	131,81	30,17	27,99	19,59	-1,69	8,25E-02	+	*Tsen2*	XM_006506336.2	=
0,00	0,00	0,00	107,15	271,90	308,13	10,28	3,47E-10	+	*Tsen34*	XM_017312225.1	=
6098,19	5525,12	5637,54	2455,03	2423,09	2219,22	-1,28	4,55E-35	-	*Tspan14*	NM_145928.2	=
83,03	100,18	74,08	848,86	840,69	101,52	2,80	6,71E-02	+	*Ttc27*	XM_006525025.3	=
157,66	250,01	4,36	0,00	0,00	0,00	-9,58	3,29E-03	-	*Ttc39b*	XM_011250101.2	=
8,41	138,31	20,70	2,08	0,00	0,00	-6,37	4,19E-02	+	*Tti2*	NM_001199988.1	=
9,46	44,33	7,63	0,00	0,00	1,78	-5,04	8,74E-02	+	*Ttll9*	XM_006500320.1	=
1812,01	537,26	28,32	10,40	10,00	0,00	-6,87	9,87E-03	+	*Tulp4*	NM_001103181.1	=
2802,10	166,68	0,00	14,56	3,00	2,67	-7,20	8,07E-02	+	*Tusc3*	XM_017313023.1	=
0,00	0,00	0,00	8,32	8,00	2,67	5,09	7,90E-02	+	*Tusc3*	XM_017313029.1	=
0,00	1,77	1,09	252,78	47,98	72,13	6,99	1,50E-05	+	*Tusc3*	XR_001778489.1	=
72,52	121,46	45,75	164,36	402,85	398,96	2,01	5,61E-02	-	*Txndc11*	NM_029582.2	=
32,58	68,27	59,92	99,87	544,80	621,59	2,98	2,14E-02	-	*Txndc11*	XM_017316842.1	=
0,00	0,00	1,09	44,73	57,98	40,96	7,05	2,93E-06	-	*Txndc11*	XM_006521662.2	=
85,14	1,77	167,76	0,00	2,00	2,67	-5,75	5,24E-02	+	*Uaca*	XM_006511483.3	=
275,38	125,89	159,05	62,42	54,98	90,83	-1,43	9,94E-02	-	*Ubac1*	XM_006498465.1	=
2881,98	0,00	3696,26	0,00	0,00	0,00	-13,58	3,02E-02	+	*Ubap1*	XM_006538198.3	=
3,15	108,16	76,26	1,04	0,00	2,67	-5,64	3,39E-02	-	*Ubap2l*	XM_017319769.1	=
0,00	0,00	0,00	382,82	423,84	302,78	10,97	4,43E-16	-	*Ubap2l*	XM_011240246.2	=
2,10	155,15	289,77	3,12	0,00	2,67	-6,27	3,08E-02	-	*Ube2e2*	XM_017315960.1	=
0,00	0,00	0,00	0,00	774,71	1172,84	11,78	9,97E-02	-	*Ube4a*	XM_011242395.2	=
43,09	12,41	18,52	10,40	732,73	1011,65	4,57	6,27E-02	+	*Ubp1*	NM_013699.2	=
40,99	18,62	23,97	15,60	683,74	1368,75	4,63	3,75E-02	+	*Ubp1*	NM_001083319.1	=
660,06	29,26	3468,58	69,70	25,99	3,56	-5,39	5,35E-02	+	*Ubr3*	XM_006500116.3	=
378,38	525,74	0,00	2,08	0,00	2,67	-7,57	7,52E-02	-	*Ubtf*	XM_017314431.1	=
337,39	445,95	86,06	84,26	42,98	39,18	-2,39	9,04E-02	-	*Ubtf*	XM_017314430.1	=
0,00	0,00	0,00	128,99	28,99	85,49	8,78	5,32E-06	+	*Ubxn11*	XR_390778.3	=
0,00	0,00	0,00	1743,49	0,00	1147,01	12,35	7,12E-02	+	*Uchl3*	NM_016723.2	=
34,68	21,28	15,25	1262,88	132,95	81,93	4,38	1,82E-02	+	*Uchl5*	XR_387185.3	=
2222,97	914,06	0,00	0,00	0,00	0,00	-12,51	6,39E-02	-	*Uevld*	XR_001785588.1	=

Additional file1: Table S1. Differentially expressed transcripts (*continued*)

Control1	Control2	Control3	PB1	PB2	PB3	log2FoldChange	P.adj	Strand	Gene Name	Transcript ID	Class Code
11,56	67,38	4,36	1,04	0,00	0,89	-5,43	5,77E-02	+	*Ufd1l*	XM_006522006.3	=
211,26	356,40	154,69	5,20	6,00	19,59	-4,54	2,53E-07	+	*Ufd1l*	XM_006522005.3	=
0,00	0,00	0,00	14,56	7,00	76,59	7,47	1,54E-02	-	*Uggt2*	XM_017316121.1	=
60,96	92,20	52,29	29,13	16,99	12,47	-1,82	2,26E-02	+	*Ugt1a6a*	NM_145079.3	=
0,00	0,89	0,00	210,13	201,92	402,52	9,56	1,92E-10	-	*Unc13d*	XM_006534188.2	=
1,05	2,66	5,45	1065,23	952,64	2,67	7,79	8,57E-04	-	*Urgcp*	XM_011243755.2	=
96,70	6005,65	5603,76	34,33	104,96	30,28	-6,11	1,06E-03	+	*Usp11*	XM_017318468.1	=
97,75	31,03	8,72	5381,29	101,96	5107,23	6,27	2,11E-03	+	*Usp11*	XM_017318470.1	=
0,00	0,00	0,00	0,00	3886,55	823,75	13,05	4,37E-02	-	*Usp15*	XM_006513227.3	=
0,00	0,00	0,00	10,40	44,98	15,14	6,99	2,68E-03	+	*Usp29*	XM_017322424.1	=
0,00	0,00	0,00	18,72	15,99	13,36	6,43	9,66E-05	-	*Usp3*	NM_001302122.1	=
0,00	0,89	2,18	2200,16	8,00	2575,43	10,62	1,85E-05	-	*Usp36*	XM_011249273.2	=
242,79	115,25	63,18	2052,44	1590,41	1482,74	3,61	8,98E-07	+	*Vps13b*	XM_006520138.3	=
0,00	14,19	10,89	4,16	646,76	821,97	5,87	9,99E-02	-	*Vps37d*	XM_011240873.2	=
2181,98	62,06	2089,43	146,68	21,99	43,64	-4,35	6,22E-02	-	*Vps39*	NM_147153.3	=
3206,75	360,84	3269,23	279,83	252,91	383,82	-2,90	6,18E-02	-	*Vps39*	NM_178851.3	=
0,00	0,00	0,00	0,00	6733,48	5654,91	27,54	6,38E-10	+	*Vps72*	XM_017319529.1	=
21,02	120,57	6,54	0,00	0,00	0,00	-8,11	1,22E-02	+	*Vsig1*	NM_030181.3	=
518,17	826,29	101,31	0,00	0,00	0,00	-11,39	3,49E-08	-	*Wbscr16*	XR_377117.3	=
13,66	13,30	23,97	0,00	0,00	1,78	-4,73	8,48E-03	+	*Wbscr27*	XM_006504532.3	=
3311,86	0,00	2804,06	0,00	0,00	0,00	-13,47	3,25E-02	-	*Wdr3*	XM_006501542.2	=
0,00	0,89	0,00	83,22	1,00	28,50	6,70	7,45E-02	-	*Wdr62*	XM_011250540.2	=
0,00	0,00	0,00	6,24	40,98	3,56	6,52	8,51E-02	-	*Wdr78*	XR_376318.2	=
2981,83	0,00	3589,50	0,00	0,00	0,00	-13,58	3,02E-02	-	*Wdr81*	XR_388371.3	=
2,10	190,61	183,02	0,00	4,00	1,78	-6,02	4,01E-02	+	*Wdr93*	XM_006541049.3	=
2,10	9,75	19,61	0,00	0,00	0,00	-5,87	9,39E-02	+	*Wdr93*	XM_017322438.1	=
1,05	62,95	96,95	0,00	0,00	0,00	-8,23	4,33E-02	-	*Wdsub1*	XM_006500225.3	=
0,00	0,00	3,27	56,17	26,99	31,17	5,20	1,78E-03	-	*Wfdc3*	NM_027961.1	=
116,67	176,43	155,78	0,00	3,00	2,67	-6,27	1,66E-15	-	*Wipf1*	NM_001289723.1	=
2,10	3,55	3,27	1,04	834,69	349,09	7,05	4,63E-03	+	*Wipf2*	XM_006534059.3	=
49,40	40,78	26,15	10,40	1,00	8,91	-2,52	8,58E-02	+	*Wipf3*	XM_006506295.3	=
4,20	82,45	140,53	0,00	1,00	3,56	-5,61	3,49E-02	-	*Wnt2*	NM_023653.5	=
33,63	9,75	373,66	3,12	3,00	4,45	-5,30	1,59E-02	-	*Wrn*	XM_017312662.1	=
59,91	0,89	40,31	1,04	0,00	0,00	-6,59	7,39E-02	-	*Xlr3a*	XM_011247559.1	=
0,00	0,89	0,00	234,06	62,98	165,64	8,74	3,56E-07	-	*Xlr5a*	XM_006528206.3	=
0,00	0,89	0,00	470,20	391,85	268,94	10,03	1,46E-12	-	*Xpnpep1*	XM_011247159.2	=
149,25	186,18	19,61	0,00	3,00	0,89	-6,51	3,67E-04	-	*Zbtb16*	NM_001033324.2	=
146,10	1,77	154,69	0,00	0,00	0,00	-9,14	1,36E-02	-	*Zbtb34*	XM_006498041.3	=
3,15	6,21	7,63	101,95	12,00	38,29	3,16	6,85E-02	-	*Zbtb37*	XM_006496841.3	=
38,89	54,08	32,68	13,52	13,99	9,80	-1,76	3,50E-03	-	*Zbtb46*	NM_027656.2	=
31,53	54,97	22,88	6,24	3,00	10,69	-2,45	2,98E-02	-	*Zbtb48*	XM_006538451.2	=
139,79	130,33	138,35	1,04	0,00	0,89	-7,71	7,80E-11	-	*Zbtb7b*	XM_006501316.2	=
13,66	189,73	27,23	0,00	0,00	0,00	-8,74	3,02E-03	-	*Zc3h11a*	XM_011248074.2	=
8,41	5,32	8,72	19,77	23,99	19,59	1,51	5,77E-02	+	*Zc3h7b*	XM_017316520.1	=
70,42	42,56	94,78	17,68	16,99	21,37	-1,88	4,98E-03	+	*Zcchc16*	XM_006528952.2	=
2,10	0,89	2,18	61,38	42,98	1,78	4,37	8,27E-02	+	*Zcchc2*	NM_001122676.1	=
0,00	0,00	0,00	2047,24	176,93	1757,92	12,81	5,19E-09	-	*Zdhhc20*	XR_001781166.1	=
224,92	349,31	19,61	0,00	0,00	0,00	-10,11	7,87E-05	-	*Zdhhc23*	XM_017317055.1	=
16,82	16,84	44,66	0,00	1,00	1,78	-4,76	2,88E-03	-	*Zdhhc23*	NM_001007460.1	=
6,31	1,77	2,18	299,60	23,99	210,17	5,71	1,62E-04	-	*Zdhhc23*	XM_011245939.1	=
59,91	60,29	72,99	11,44	7,00	5,34	-3,04	3,84E-10	-	*Zdhhc4*	XM_006504731.1	=
3,15	1,77	5,45	0,00	3601,65	3706,41	9,46	2,17E-03	-	*Zeb2*	XM_017318061.1	=
4,20	9,75	3,27	7,28	1035,61	339,29	6,32	3,02E-03	-	*Zfp217*	XM_006499432.3	=
107,21	127,67	108,94	224,70	374,86	183,45	1,19	9,54E-02	-	*Zfp219*	XM_006519503.2	=
0,00	108,16	150,33	0,00	1,00	0,89	-7,08	9,26E-02	-	*Zfp236*	XM_006526496.3	=
11,56	9,75	11,98	0,00	0,00	0,89	-4,99	9,50E-03	-	*Zfp27*	XM_017322132.1	=
982,73	1017,79	825,75	470,20	522,80	292,99	-1,14	2,47E-02	+	*Zfp280b*	XM_006513943.2	=
2,10	610,85	416,14	5,20	5,00	5,34	-6,05	8,92E-03	-	*Zfp280c*	XM_006541461.3	=
25,23	18,62	19,61	55,13	40,98	73,02	1,42	2,36E-02	+	*Zfp280d*	XM_017313342.1	=
7,36	1,77	11,98	482,68	514,81	4,45	5,57	2,07E-02	-	*Zfp329*	XM_006540315.3	=
0,00	0,00	2,18	2,08	82,97	90,83	6,38	7,22E-02	-	*Zfp37*	NM_001290353.1	=
84,08	66,49	84,97	18,72	24,99	29,39	-1,68	2,10E-05	+	*Zfp384*	XM_006506201.2	=
48,35	161,36	55,56	752,11	618,77	480,00	2,80	1,22E-03	+	*Zfp397*	XM_011246996.2	=
0,00	0,00	3,27	27,05	22,99	24,94	4,62	3,81E-04	-	*Zfp426*	XM_011242460.2	=
0,00	0,00	2,18	21,85	18,99	17,81	4,86	1,59E-03	-	*Zfp426*	XM_011242465.1	=
0,00	0,00	1,09	23,93	21,99	21,37	5,96	2,39E-04	-	*Zfp426*	XM_011242459.2	=
0,00	0,00	0,00	21,85	19,99	21,37	6,83	9,60E-06	-	*Zfp426*	XM_011242462.2	=
0,00	0,00	0,00	29,13	23,99	25,83	7,15	2,17E-06	-	*Zfp426*	XM_011242469.2	=
0,00	457,47	2004,46	0,00	0,00	0,00	-12,16	7,93E-02	-	*Zfp445*	XM_011242971.1	=
303,75	226,08	1761,53	8,32	13,00	0,89	-6,69	1,92E-05	-	*Zfp445*	NM_173364.5	=
1,05	0,00	0,00	937,28	1,00	81,93	9,88	9,20E-03	-	*Zfp445*	XM_006512121.3	=
1,05	338,67	473,88	10,40	1,00	8,91	-5,32	9,40E-02	+	*Zfp446*	XM_006540024.3	=
298,50	231,40	296,31	0,00	0,00	0,00	-10,58	3,51E-15	+	*Zfp46*	XM_006538732.1	=
2,10	0,89	2,18	3008,45	1525,43	1,78	9,79	4,52E-05	+	*Zfp462*	NM_172867.3	=
1787,84	1744,78	1992,47	920,64	851,68	810,39	-1,10	3,06E-16	-	*Zfp512b*	XM_006500647.3	=
0,00	0,00	0,00	0,00	3383,74	3766,97	13,65	2,86E-02	-	*Zfp512b*	NM_001164597.1	=
0,00	0,00	5,45	10,40	265,90	243,12	6,60	5,32E-02	+	*Zfp523*	XR_385316.3	=
5,26	12,41	19,61	0,00	0,00	0,00	-6,11	9,19E-03	-	*Zfp608*	XM_006525958.3	=
792,49	216,32	612,23	175,80	155,94	109,54	-1,88	9,22E-02	+	*Zfp808*	NM_001039239.2	=
1,05	3,55	10,89	155,00	93,96	8,91	4,06	8,13E-02	+	*Zfp827*	XM_011248477.2	=
31,53	11,53	46,84	0,00	0,00	0,00	-7,38	3,46E-04	-	*Zfp868*	XM_011242294.1	=
1,05	122,35	89,33	3,12	1,00	0,89	-5,42	4,45E-02	+	*Zfp90*	XM_006530850.3	=
1,05	296,12	119,83	5,20	5,00	0,89	-5,24	7,65E-02	+	*Zfp90*	XM_006530848.3	=
42,04	70,93	13,07	242,38	367,86	586,86	3,24	2,61E-03	+	*Zfp93*	XM_017322141.1	=
3,15	263,31	218,97	0,00	0,00	0,00	-9,82	4,24E-03	-	*Zfx*	XM_017318466.1	=

Additional file1: Table S1. Differentially expressed transcripts (*continued*)

Control1	Control2	Control3	PB1	PB2	PB3	log2FoldChange	P.adj	Strand	Gene Name	Transcript ID	Class Code
1226,58	2209,34	93,69	61,38	62,98	78,37	-4,12	1,17E-02	-	*Zfx*	XM_011247578.2	=
43,09	40,78	52,29	10,40	1,00	0,89	-3,49	4,03E-02	-	*Zfx*	XM_011247577.2	=
28,38	31,92	15,25	1,04	1,00	5,34	-3,32	1,04E-02	-	*Zfyve16*	XR_382730.3	=
10,51	8,87	7,63	0,00	0,00	0,00	-5,65	2,83E-03	-	*Zfyve28*	XR_868282.2	=
3547,30	445,06	2678,78	216,38	476,82	273,39	-2,79	7,12E-02	-	*Zfyve9*	XM_006502989.3	=
2,10	0,00	1,09	140,44	93,96	160,30	7,03	6,93E-11	+	*Zkscan1*	XM_011240958.2	=
119,82	143,62	181,93	2740,06	141,95	3276,28	3,79	3,39E-02	-	*Zmym5*	NM_144842.4	=
29,43	34,58	210,25	11,44	11,00	12,47	-2,97	9,45E-02	-	*Znhit3*	XM_006533672.1	=
309,01	15,96	895,47	18,72	27,99	28,50	-4,02	7,43E-02	-	*Zrsr2*	NM_009453.3	=
98,80	285,48	114,38	0,00	0,00	0,00	-9,86	7,99E-09	-	*Zscan26*	XM_006516711.3	=
0,00	0,00	0,00	81,14	48,98	73,91	8,52	7,39E-09	-	*Zyg11a*	XM_017320136.1	=
0,00	0,00	0,00	1867,28	0,00	2088,31	12,80	5,25E-02	-	*Nphs1*	XM_011250647.1	i
0,00	0,00	0,00	0,00	1623,39	2566,53	12,88	4,97E-02	+	*Tbce*	XM_017315601.1	i
505,56	704,83	1131,86	4327,50	3164,82	1846,97	2,00	1,45E-02	+	*4932438A13Rik*	XM_006535446.3	j
543,39	576,27	307,20	1146,37	991,63	881,63	1,08	9,15E-02	-	*9930021J03Rik*	XM_006527070.3	j
1841,44	0,00	815,94	0,00	0,00	0,00	-12,27	7,43E-02	+	*A830080D01Rik*	XM_006528900.3	j
2633,93	2677,45	2913,00	485,80	373,86	520,96	-2,57	1,18E-35	-	*Abcc5*	XM_006522212.3	j
1131,98	192,39	373,66	0,00	0,00	0,00	-11,62	3,33E-09	+	*Acaa1a*	XM_006511923.1	j
130,33	1158,75	2422,78	122,75	105,96	92,62	-3,53	3,65E-02	+	*Acaca*	XM_006531953.1	j
566,52	5850,49	4981,73	631,44	369,86	390,05	-3,03	5,55E-02	+	*Acaca*	XM_006531953.1	j
12,61	9,75	40,31	34,33	616,77	181,67	3,73	7,73E-02	-	*Acads*	NM_007383.3	j
5619,97	3324,65	0,00	0,00	0,00	0,00	-14,02	2,16E-02	-	*Actb*	NM_007393.5	j
2325,97	2209,34	2048,03	1102,68	818,69	935,95	-1,20	9,02E-09	+	*Agbl5*	XM_006503868.3	j
686,34	1251,84	612,23	311,04	314,88	172,76	-1,68	3,07E-02	-	*Aldh3a2*	XM_006532029.3	j
0,00	0,00	0,00	0,00	1533,43	460,41	11,81	9,78E-02	+	*Alms1*	XM_006506051.3	j
78,83	102,84	117,65	247,58	625,77	198,59	1,84	9,09E-02	+	*Amhr2*	XM_006520293.2	j
4,20	7,98	13,07	514,93	226,92	7,12	4,89	2,56E-02	+	*Ankhd1*	NM_175375.3	j
831,38	771,32	1126,42	0,00	1,00	0,00	-11,35	4,80E-17	+	*Apcdd1*	NM_133237.3	j
1298,05	4666,92	1856,30	0,00	0,00	0,00	-13,83	2,08E-16	-	*Arel1*	NM_178065.4	j
3970,87	5524,24	0,00	0,00	0,00	0,00	-14,11	2,03E-02	-	*Arhgap21*	XM_006497541.3	j
2381,68	2234,16	0,00	0,00	1,00	0,00	-12,11	1,10E-02	+	*Armt1*	XM_011243101.2	j
1,05	1,77	1,09	1391,88	2,00	2493,50	9,95	3,16E-05	+	*Ascc2*	NM_029291.1	j
0,00	1995,68	453,18	0,00	0,00	0,00	-12,15	7,97E-02	+	*Asxl3*	NM_001167777.1	j
54,65	319,17	3142,86	0,00	1,00	0,00	-11,71	9,47E-06	+	*Atl3*	NM_001163505.1	j
507,66	533,72	537,06	71,78	191,93	113,10	-2,07	7,80E-04	+	*Bcas3*	XM_006532537.3	j
148,20	8,87	89,33	5,20	6,00	1,78	-4,25	2,05E-02	+	*C1d*	NM_020558.3	j
6218,01	0,00	539,24	0,00	0,00	0,00	-27,39	8,24E-10	-	*Calr*	NM_007591.3	j
3,15	0,00	0,00	4637,50	522,80	0,00	10,68	7,75E-02	+	*Cbfa2t2*	XM_006498640.2	j
0,00	1,77	0,00	1877,68	5231,05	1,78	11,92	5,22E-04	+	*Cbfa2t2*	XM_006498640.2	j
0,00	0,00	1,09	4602,14	918,66	0,00	12,32	1,40E-02	+	*Cbx1*	XM_017314242.1	j
0,00	0,00	0,00	0,00	773,71	1340,26	11,90	9,31E-02	+	*Ccdc80*	XM_006522498.2	j
68,32	5,32	663,43	0,00	0,00	0,00	-10,42	5,05E-03	-	*Cdc40*	NM_027879.2	j
397,30	23,05	958,65	16,64	20,99	17,81	-4,64	1,12E-02	-	*Celf2*	NM_001110231.1	j
406,76	1676,51	1119,88	3451,60	4266,41	4704,70	1,96	7,52E-02	-	*Celf2*	NM_001110231.1	j
0,00	0,00	0,00	141,48	0,00	3961,11	26,02	7,90E-09	-	*Cfh*	NM_009888.3	j
0,00	2178,31	1806,19	0,00	0,00	0,00	-12,85	5,06E-02	+	*Cflar*	XM_011238420.2	j
218,62	418,46	386,73	6450,69	1459,45	830,87	3,09	4,33E-02	-	*Chd6*	NM_173368.3	j
3349,70	1282,87	0,00	0,00	0,00	0,00	-13,07	4,31E-02	+	*Clasp1*	XM_006529920.1	j
12,61	11,53	3,27	29,13	216,92	102,41	3,66	1,40E-02	-	*Col13a1*	XM_011243332.2	j
4731,83	5988,80	0,00	0,00	0,00	0,00	-28,01	2,93E-10	+	*Col16a1*	XM_006502641.2	j
0,00	0,00	0,00	0,00	2712,99	6529,41	25,55	1,70E-08	+	*Col16a1*	XM_006502641.2	j
6407,20	0,00	460,81	0,00	0,00	0,00	-27,41	7,97E-10	-	*Col6a3*	NM_001243008.1	j
28,38	16,84	20,70	509,73	51,98	73,02	3,27	8,21E-02	+	*Cs*	NM_026444.3	j
1365,31	0,00	830,11	0,00	0,00	0,00	-11,99	8,78E-02	+	*Csnk2a1*	XM_006498657.3	j
388,89	14,19	1443,43	45,77	8,00	21,37	-4,62	8,11E-02	+	*Csnk2a1*	XM_006498657.3	j
1,05	0,00	0,00	1073,55	0,00	47,20	10,02	8,51E-02	+	*Csnk2a1*	XM_006498657.3	j
229,13	1,77	568,66	1,04	0,00	0,00	-9,58	3,37E-03	-	*D5Ertd579e*	XM_011240743.2	j
0,00	0,00	0,00	1883,92	3585,66	0,00	13,27	3,78E-02	+	*Ddah1*	NM_026993.3	j
0,00	0,00	0,00	0,00	1367,49	4503,44	13,37	3,51E-02	-	*Dgkz*	XM_006498537.1	j
0,00	0,00	0,00	0,00	3389,73	2957,47	13,48	3,24E-02	+	*Dlk1*	NM_001190705.1	j
0,00	0,00	0,00	809,33	0,00	2415,13	12,51	6,41E-02	-	*Dock9*	XM_006518354.3	j
20002,54	0,00	18040,11	0,00	0,00	0,00	-29,75	1,44E-11	-	*Eif5a*	NM_001166589.1	j
0,00	0,00	0,00	4001,90	4144,45	0,00	13,84	2,48E-02	-	*Elmo2*	NM_207706.1	j
14092,48	5469,27	1193,96	0,00	0,00	0,00	-15,24	5,86E-13	-	*Eln*	NM_007925.4	j
2006,46	2116,25	0,00	0,00	0,00	0,00	-12,90	4,88E-02	+	*Epcam*	NM_008532.2	j
6096,09	2884,91	0,00	0,00	1,00	0,00	-13,07	5,82E-03	+	*Fam129a*	NM_022018.3	j
3,15	6,21	0,00	191,41	50,98	30,28	4,84	1,42E-02	-	*Fnbp1*	XM_006497685.1	j
0,00	0,00	0,00	0,00	19638,66	21804,79	29,26	3,51E-11	-	*Glg1*	NM_009149.2	j
0,00	0,00	0,00	3938,45	4129,46	0,00	13,83	2,50E-02	+	*Gli3*	NM_008130.2	j
1310,66	5,32	1381,33	7,28	10,00	8,01	-6,74	1,49E-03	+	*Gm11007*	XR_001783630.1	j
1,05	0,89	4,36	61,38	1,00	184,34	5,31	7,59E-02	-	*Gm13157*	NM_001127189.3	j
60,96	25,71	20,70	237,18	142,95	218,18	2,48	1,88E-03	+	*Gm14692*	NM_001163195.1	j
13709,90	0,00	8170,34	0,00	0,00	0,00	-28,98	5,61E-11	-	*Gm16867*	XR_874771.2	j
3401,20	0,00	3884,72	0,00	0,00	0,00	-13,73	2,71E-02	-	*Gm16867*	XR_874771.2	j
729,43	1500,08	11,98	20,81	1,00	0,00	-6,69	6,97E-02	+	*Gm21451*	XR_001781313.1	j
4,20	5,32	6,54	6,24	35,99	195,92	3,89	9,87E-02	+	*Gm9222*	XR_001780559.1	j
2,10	0,89	0,00	59,30	8627,78	9194,79	12,54	1,23E-08	+	*Golga4*	XR_379875.3	j
420,42	5627,08	0,00	0,00	0,00	0,00	-26,47	3,91E-09	+	*Gon4l*	XM_006502240.3	j
0,00	0,00	0,00	349,53	103,96	92,62	9,94	1,63E-07	+	*Gpc1*	XM_006529142.2	j
89,34	401,62	199,36	41,61	21,99	61,45	-2,46	6,44E-02	+	*Gprasp1*	NM_026081.5	j
0,00	0,00	0,00	91,54	0,00	1865,67	11,78	9,94E-02	-	*H2-K1*	XM_006523674.3	j
41834,96	0,00	10659,57	0,00	0,00	0,00	-30,00	8,93E-12	+	*Hba-a2*	NM_001083955.1	j
295967,39	419075,93	0,00	0,00	0,00	0,00	-30,00	8,93E-12	-	*Hbb-bs*	NM_001201391.1	j
0,00	0,00	0,00	992,41	1199,55	976,92	12,48	8,42E-22	+	*Hdhd2*	NM_001039201.1	j
2383,78	2122,46	1128,60	10,40	23,99	7,12	-7,09	2,51E-24	-	*Heatr5a*	XM_006516010.2	j

Additional file1: Table S1. Differentially expressed transcripts (*continued*)

Control1	Control2	Control3	PB1	PB2	PB3	log2FoldChange	P.adj	Strand	Gene Name	Transcript ID	Class Code
0,00	2383,11	2883,59	0,00	0,00	0,89	-12,30	9,80E-03	-	*Hip1*	XM_011240879.2	j
0,00	0,00	0,00	9259,40	7938,03	7851,86	15,46	2,13E-34	+	*Hmgcs1*	NM_145942.5	j
24594,58	48780,13	32204,22	0,00	0,00	0,00	-17,58	2,25E-37	+	*Hnrnpa1*	XR_383983.1	j
2138,89	5131,48	6165,88	40477,77	64473,92	40193,47	3,43	9,53E-08	+	*Hnrnpa1*	XR_383983.1	j
0,00	0,89	0,00	532,62	943,65	0,00	10,42	3,83E-02	+	*Immp1l*	NM_028260.2	j
175,53	52,31	290,86	0,00	0,00	2,67	-7,54	3,69E-05	+	*Ipo5*	XM_006519538.3	j
1685,88	1618,00	1214,66	97,78	330,88	59,67	-3,21	3,38E-03	-	*Kansl1*	XM_006534448.3	j
3625,07	11583,08	5215,95	0,00	0,00	0,00	-15,21	1,35E-21	+	*Kctd14*	XM_006507608.3	j
0,00	0,00	0,00	2185,60	205,92	2353,69	13,06	2,49E-09	-	*Kif1a*	XM_006529155.3	j
0,00	0,00	0,00	25015,27	0,00	25154,98	29,41	2,63E-11	+	*Lamp1*	NM_001317353.1	j
4071,77	3988,69	411,79	468,12	383,86	292,99	-2,89	7,75E-02	+	*Lgr4*	NM_172671.2	j
1157,21	2093,20	1088,29	432,75	455,83	415,88	-1,73	3,79E-04	+	*Lmo2*	NM_001142336.1	j
135,59	76,25	122,01	308,96	193,93	374,92	1,40	5,77E-02	+	*LOC101055907*	XM_011247886.2	j
0,00	0,00	0,00	0,00	2511,06	2216,55	13,06	4,35E-02	+	*Ltbp3*	NM_008520.2	j
0,00	4531,27	4077,54	0,00	0,00	0,00	-13,97	2,26E-02	-	*Macf1*	NM_001199136.1	j
536,04	1248,29	175,39	13134,39	4967,14	3106,19	3,44	2,42E-02	-	*Macf1*	NM_001199136.1	j
588,59	289,02	973,90	9401,92	10016,26	8943,66	3,94	1,59E-10	-	*Man2a2*	XM_006540599.3	j
3104,80	0,00	1538,20	0,00	0,00	0,00	-13,08	4,30E-02	-	*Metap2*	XM_006513896.3	j
0,00	0,00	0,00	0,00	1321,51	1313,54	12,21	7,68E-02	-	*Mfap1b*	NM_001081975.3	j
422,52	187,07	214,61	996,57	4021,50	1293,06	2,94	9,23E-03	+	*Mga*	XM_017318963.1	j
0,00	1,77	0,00	2,08	358,87	414,99	8,71	8,55E-03	+	*Mgat3*	XM_011245485.2	j
0,00	0,00	0,00	1969,22	0,00	3522,96	13,27	3,76E-02	+	*Midn*	XM_006513913.3	j
106,16	3974,51	3063,33	218,46	215,92	149,61	-3,61	9,66E-02	-	*Mki67*	NM_001081117.2	j
2846,24	0,00	1631,89	0,00	0,00	0,00	-13,02	4,47E-02	+	*Mrpl45*	NM_025927.4	j
163,96	113,48	116,56	10396,41	101,96	9925,92	5,70	3,38E-03	+	*Msl1*	XM_011249283.2	j
0,00	0,00	0,00	458,76	2025,24	867,38	12,56	1,46E-12	-	*Mxd4*	NM_010753.2	j
0,00	0,00	0,00	1169,26	0,00	1071,32	11,98	8,86E-02	+	*Myh8*	NM_177369.3	j
0,00	7,09	0,00	2,08	257,90	1259,22	7,73	9,16E-02	-	*N4bp3*	XM_006532765.3	j
3988,74	163,13	4033,97	183,09	138,95	116,66	-4,22	1,44E-02	-	*Necab3*	NM_021546.3	j
0,00	0,00	0,00	635,60	5343,00	0,00	26,55	3,41E-09	-	*Nf2*	NM_001252250.1	j
1538,74	2471,77	1012,03	0,00	0,00	0,00	-13,19	7,97E-19	-	*Nr3c1*	XM_006525665.3	j
120,87	11075,96	8310,87	87,38	129,95	116,66	-5,87	1,49E-03	+	*Nrd1*	XM_006502990.3	j
7,36	0,89	8,72	545,10	71,97	21,37	5,24	1,90E-02	+	*Nrxn2*	NM_001205234.1	j
0,00	649,86	2138,45	0,00	0,00	0,00	-12,34	7,15E-02	+	*Ocrl*	XM_011251008.2	j
70,42	8,87	34,86	212,21	374,86	625,16	3,41	1,18E-02	+	*Odf2l*	XM_006501679.3	j
5,26	5,32	2,18	5,20	153,94	215,51	4,87	1,72E-02	+	*Osbpl2*	XM_017317993.1	j
0,00	0,00	0,00	7023,87	0,00	6947,97	27,73	4,61E-10	-	*Pacsin2*	XM_011245633.2	j
0,00	0,00	0,00	0,00	3182,81	2286,01	13,27	3,78E-02	+	*Papln*	XM_011244005.2	j
3033,33	3431,04	3178,81	639,76	855,68	309,91	-2,42	4,77E-05	-	*Paxip1*	NM_018878.3	j
234,38	226,96	223,32	2316,67	2755,97	2385,75	3,45	4,23E-124	-	*Paxip1*	NM_018878.3	j
4313,51	5,32	432,48	12,48	3,00	3,56	-7,97	1,43E-03	+	*Pcbp2*	NM_001103165.1	j
0,00	7556,26	8039,62	0,00	0,00	0,00	-28,53	1,22E-10	+	*Pias2*	NM_001164168.1	j
19,97	21,28	26,15	556,54	936,65	21,37	4,49	1,72E-02	+	*Pkp4*	XM_017317535.1	j
44,14	53,19	61,01	2318,75	4336,38	16,92	5,40	1,99E-02	-	*Plec*	NM_001163542.1	j
60,96	46,10	64,27	4463,78	4788,21	48,98	5,76	2,65E-03	-	*Plec*	NM_001163542.1	j
75,68	58,51	91,51	5880,62	8470,84	60,56	6,00	2,24E-03	-	*Plec*	NM_001163542.1	j
96,70	43,44	101,31	10165,48	10223,18	72,13	6,41	1,23E-03	-	*Plec*	NM_001163542.1	j
444,59	390,09	406,34	22284,57	12452,35	309,91	4,82	1,39E-02	+	*Ppp1r9b*	NM_172261.3	j
0,00	0,00	0,00	406,74	4399,36	0,00	26,24	5,61E-09	+	*Ppp1r9b*	NM_172261.3	j
5993,09	0,00	8233,52	0,00	0,00	0,00	-28,41	1,51E-10	-	*Ppp6r1*	NM_172894.2	j
4580,48	0,00	6072,20	0,00	0,00	0,00	-28,01	2,95E-10	-	*Ppp6r1*	NM_172894.2	j
0,00	0,00	0,00	0,00	2818,95	2865,75	13,32	3,65E-02	-	*Ppp6r1*	NM_172894.2	j
0,00	0,00	0,00	5989,85	0,00	8582,99	27,66	5,20E-10	-	*Ppp6r1*	NM_172894.2	j
44,14	274,84	44,66	102,99	7148,33	7492,97	5,34	2,32E-02	+	*Psmc2*	NM_011188.3	j
2108,41	2198,70	1977,22	9,36	13,00	12,47	-7,49	1,25E-145	-	*Rffl*	XM_017314715.1	j
4630,93	5938,27	0,00	0,00	0,00	0,00	-28,00	2,97E-10	-	*Rnf187*	NM_022423.2	j
492,94	0,89	8479,72	0,00	0,00	0,00	-14,03	2,10E-03	-	*Rpl15*	NM_025586.3	j
9,46	6,21	0,00	432,75	212,92	5,34	5,37	9,48E-02	-	*Samd11*	XM_006538839.3	j
904,95	0,00	1108,99	0,00	0,00	0,00	-11,87	9,45E-02	+	*Sarnp*	XM_011243523.2	j
951,20	7,98	1122,06	6,24	4,00	0,00	-7,67	3,38E-03	+	*Sarnp*	XM_011243523.2	j
30,48	30,14	37,04	4,16	13,99	13,36	-1,62	9,51E-02	+	*Scaf8*	NM_134123.3	j
1009,01	1495,65	495,67	0,00	0,00	0,00	-12,45	3,47E-15	+	*Setd2*	XR_001778893.1	j
0,00	0,00	0,00	1331,54	0,00	2688,53	12,82	5,18E-02	+	*Sfswap*	XM_006504303.3	j
1227,63	1028,42	527,26	0,00	0,00	0,00	-12,34	1,20E-16	+	*Sirpa*	XM_017316564.1	j
0,00	0,00	0,00	16356,10	5576,92	1057,96	15,34	5,91E-12	-	*Soat1*	NM_009230.3	j
0,00	0,00	0,00	0,00	2313,14	2094,54	12,96	4,68E-02	-	*Spc24*	NM_026282.5	j
1042,64	1617,11	0,00	0,00	0,00	0,00	-12,27	7,43E-02	-	*Sppl2a*	XM_017319182.1	j
877,63	859,98	749,49	369,29	302,89	317,92	-1,33	1,47E-13	-	*Tefm*	XM_011249204.2	j
5360,36	894,55	2,18	0,00	0,00	0,00	-13,51	8,58E-04	-	*Thoc2*	XM_006541503.3	j
1919,22	166,68	0,00	0,00	0,00	0,89	-10,96	3,89E-02	-	*Thoc2*	XM_006541503.3	j
390,99	500,03	323,55	0,00	0,00	0,00	-11,14	4,37E-16	+	*Timm10b*	XR_001785479.1	j
26,28	6,21	5,45	120,67	87,97	59,67	2,83	2,85E-02	+	*Tle2*	NM_019725.2	j
3174,17	1,77	2624,31	4,16	6,00	1,78	-8,93	1,76E-04	+	*Tnks1bp1*	XM_006499213.3	j
6,31	12,41	7,63	112,35	775,71	72,13	5,18	4,49E-04	-	*Tns3*	XR_380972.3	j
2,10	83,34	306,12	0,00	2,00	2,67	-6,38	3,07E-02	-	*Tradd*	NM_001033161.2	j
32,58	43,44	18,52	2860,73	2234,17	16,03	5,76	8,02E-03	+	*Tsc1*	XM_011239142.2	j
318,47	418,46	411,79	8,32	0,00	0,00	-7,12	1,59E-03	-	*Tulp1*	XM_017317394.1	j
4200,00	597,55	2957,66	263,19	321,88	299,22	-3,13	6,09E-03	-	*U2surp*	NM_001114977.1	j
871,32	0,00	1696,16	0,00	0,00	0,00	-12,22	7,65E-02	+	*Uaca*	XM_017313609.1	j
186,04	218,10	249,47	239,26	3264,78	3268,27	3,37	3,94E-02	-	*Uba52*	NM_019883.3	j
7433,03	0,00	253,83	0,00	0,00	0,00	-27,56	6,11E-10	-	*Ubc*	NM_019639.4	j
1038,44	996,51	947,76	4097,61	1747,35	2017,07	1,40	7,75E-02	-	*Ubc*	NM_019639.4	j
21,02	22,16	3,27	22,89	725,73	987,60	5,22	1,18E-02	+	*Ubp1*	XR_001778854.1	j
24,17	21,28	3,27	23,93	1622,39	1151,46	5,84	5,05E-03	+	*Ubp1*	XR_001778854.1	j
44,14	1504,51	3014,31	142,52	65,98	19,59	-4,32	9,48E-02	+	*Uchl3*	XM_006519246.3	j

Additional file1: Table S1. Differentially expressed transcripts (*continued*)

Control1	Control2	Control3	PB1	PB2	PB3	log2FoldChange	P.adj	Strand	Gene Name	Transcript ID	Class Code
133,48	207,46	208,07	0,00	14,99	3,56	-4,89	5,61E-03	+	*Vprbp*	XR_379768.3	j
0,00	0,00	0,00	0,00	572,79	1755,25	12,04	8,58E-02	+	*Vps41*	NM_172120.4	j
0,00	2570,17	387,82	0,00	0,00	0,00	-12,42	6,76E-02	+	*Vps72*	NM_009336.2	j
9924,02	1,77	8781,48	3,12	4,00	0,00	-11,37	9,02E-05	+	*Wasf2*	XM_006538849.3	j
2766,36	3173,93	2318,20	0,00	0,00	0,00	-13,91	1,11E-26	-	*Wdr91*	XM_006505248.3	j
0,00	0,00	0,00	773,96	0,00	1191,54	11,79	9,90E-02	+	*Zfp395*	NM_199029.2	j
32,58	25,71	32,68	188,29	762,72	67,68	3,49	2,50E-02	+	*Zfp97*	NM_011765.5	j
124,02	1,77	229,86	1,04	0,00	0,00	-8,41	9,32E-03	+	*Zmat5*	XM_006514787.1	j
1109,91	675,57	1193,96	393,22	524,80	401,63	-1,17	3,41E-02	+	*Zmiz1*	NM_183208.4	j
0,00	0,00	0,00	135,23	273,90	39,18	9,66	4,61E-06	+	*Znrd1as*	XM_006525080.3	j
6289,49	7441,89	6989,45	544,06	428,84	1280,59	-3,20	9,47E-07	-	*Gm21596*	XM_003945339.3	k
177,63	117,03	327,90	1233,76	1983,26	1529,05	2,93	4,71E-06	-	*Gp1bb*	NM_001001999.1	k
0,00	0,00	0,00	394,26	0,00	2150,65	12,16	7,92E-02	+	*H2afj*	NM_177688.4	k
6,31	12,41	17,43	11,44	1894,29	1629,68	6,61	1,18E-03	+	*Hist3h2a*	NM_178218.4	k
4,20	83,34	340,98	6,24	1,00	8,01	-4,81	7,64E-02	-	*LOC108167523*	XR_001778519.1	k
0,00	494012,62	439930,36	0,00	0,00	0,00	-30,00	8,93E-12	-	*Mir675*	NR_030416.1	k
335,29	125,89	135,08	0,00	0,00	0,00	-10,11	1,32E-09	-	*Mydgf*	NM_080837.2	k
7,36	3,55	5,45	344,33	350,87	0,89	5,41	4,64E-02	-	*Rbm26*	NM_134077.4	k
0,00	0,00	0,00	19520,58	0,00	20671,13	28,22	2,10E-10	+	*Snord58b*	NR_028552.1	k
78,83	54,97	217,88	1100,60	1060,60	1361,63	3,33	5,44E-05	+	*Actr8*	NM_027493.3	m
536,04	1175,60	1967,42	0,00	0,00	0,00	-12,74	1,67E-14	-	*Atp5h*	XM_006534254.1	m
14,71	7,98	18,52	78,02	953,64	22,26	4,68	4,47E-02	+	*Clpp*	NM_017393.2	m
0,00	0,00	0,00	12896,17	0,00	28221,10	29,24	3,61E-11	+	*Eif5b*	NM_198303.2	m
14,71	13,30	54,47	264,23	5844,82	16,92	6,22	2,72E-02	-	*Irs1*	NM_010570.4	m
1942,34	615,28	1787,67	199,73	187,93	367,79	-2,52	5,41E-03	+	*Ly6e*	NM_001164037.1	m
201,80	40,78	159,05	0,00	0,00	0,00	-9,54	9,05E-07	+	*Nxf1*	NM_016813.2	m
2,10	315,62	520,72	1,04	1,00	0,00	-8,71	1,70E-03	-	*Ppp1r37*	NM_199149.3	m
7019,97	10797,57	0,00	0,00	6,00	3,56	-10,86	7,51E-03	+	*Prdx2*	NM_011563.6	m
0,00	3636,72	994,60	0,00	1,00	0,89	-11,25	3,38E-03	-	*Rab14*	NM_026697.3	m
0,00	0,00	3,27	143,56	4,00	131,80	6,45	5,92E-02	+	*S100a6*	NM_011313.2	m
4472,22	0,00	3899,98	0,00	0,00	0,00	-13,93	2,33E-02	+	*Sntb2*	NM_009229.4	m
1,05	0,89	1,09	101654,75	0,00	111924,41	16,11	3,28E-07	-	*Sparc*	NM_009242.5	m
2242,94	0,00	2153,70	0,00	0,00	0,00	-13,00	4,54E-02	+	*Spg21*	NM_138584.2	m
237,54	289,91	144,89	1439,73	7447,22	616,25	3,82	1,71E-02	-	*Zdhhc5*	NM_144887.4	m
97,75	120,57	88,24	286,07	960,64	586,86	2,58	6,59E-04	-	*Akap7*	XR_001779546.1	n
1048,95	687,98	226,59	33,29	42,98	88,16	-3,58	5,22E-04	+	*Ankrd31*	XM_006517797.1	n
653,75	445,95	269,08	18,72	32,99	86,38	-3,31	1,54E-03	+	*Ankrd31*	XM_006517797.1	n
506,61	686,21	252,74	3507,78	1545,42	3499,81	2,56	1,86E-03	+	*Ankrd31*	XM_006517797.1	n
1445,19	1078,07	0,00	0,00	0,00	0,00	-12,20	7,75E-02	+	*Dusp9*	XM_006528331.1	n
37,84	1031,97	10168,26	0,00	31,99	72,13	-6,75	8,93E-02	-	*Hmgn2*	NM_016957.3	n
213,36	202,14	168,85	942,48	1157,57	720,44	2,27	7,32E-13	+	*Ip6k2*	XM_006511851.3	n
141,89	166,68	247,29	1022,58	806,70	624,27	2,14	2,11E-06	+	*Mef2d*	XM_017319472.1	n
0,00	1409,65	1452,14	0,00	0,00	0,00	-12,38	6,97E-02	-	*Ndufb11*	XM_006527554.1	n
53,60	15,07	16,34	1748,69	47,98	1389,24	5,23	4,72E-03	+	*Uchl3*	XM_006519246.3	n
0,00	0,00	0,00	2071,17	2227,17	16,03	12,93	3,84E-05	+	*LOC102638448*	XR_877772.2	o
0,00	60523,67	55409,07	0,00	0,00	0,00	-30,00	8,93E-12	+	-	-	r
0,00	17,73	486,95	0,00	1,00	0,89	-8,05	9,79E-02	-	-	-	r
74,62	56,74	67,54	33,29	37,99	20,48	-1,12	8,26E-02	-	-	-	r
0,00	1086,05	3765,98	0,00	0,00	0,00	-13,14	4,11E-02	-	-	-	u
276,43	1239,43	1,09	4,16	1,00	0,00	-8,21	1,28E-02	-	-	-	u
50,45	46,99	68,63	957,04	1164,56	73,91	3,72	1,88E-02	+	-	-	u
0,00	7,09	0,00	100,91	847,68	12,47	7,06	8,16E-02	+	-	-	u
0,00	0,89	0,00	8,32	125,95	81,04	7,64	2,00E-03	-	-	-	u
0,00	12,41	0,00	2837,84	6395,61	16,92	9,53	1,27E-02	+	-	-	u
470,87	455,70	582,82	125,87	121,95	97,07	-2,13	3,17E-18	-	*Gm35533*	XR_380751.2	y

Additional file1: Table S2. Primers used in this work

gene	forward	reverse	function
Bax	TTTGCTACAGGGTTTCATCCAG	CCAGTTCATCTCCAATTCGCC	apoptosis
Bcl2	GTGTGGAGAGCGTCAACAGG	CACAAAGGCATCCCAGCCTC	apoptosis
Casp8	GGAACAGACTGTGATAAAGAGGCT	TTGTGGTCTGCGCTTTGGTA	apoptosis
Ddx4	ACAGGATGTCCCCGCATGGC	TCCCATGACTCGTCATCAACTGGA	germ cell marker
Kit	AGCGTCTTCCGGCACAACGG	AGCGTCTTCCGGCACAACGG	germ cell marker
Dazl	CAGTATGTTCAGGCATATCCTC	ATTCATTGGGCAAAATATCAGC	germ cell marker
Gadd45g	CCAAAGTCCTGAATGTGGACCC	CAGAACGCCTGAATCAACGTG	germ cell marker
Ccnd1	GGACGTCGTCAGGAGCAC	ACCGACGTGCGAGATGTG	cell cycle
Ccnd2	ACCCCTCACGACTTCATTGA	TCCAGTTGCAATCATCGACG	cell cycle
Ccne2	GAGGCATTATGACACCACCA	GAGGCATTATGACACCACCA	cell cycle
Cdk1	CCAGAGCTTGAAGATCCTGAAGA	GTACCGATCTCCAGAAGTGTTG	cell cycle
Ep300	CCTTCCACTCCGCTTTCTCA	ACCTTTAGCCTCCTTTGTATCCTC	hormonal signaling
Esr1	CACGTTTCTGTCCAGCACCTTGAAGT	AGAGATGCTCCATGCCTTTGTTACTCA	hormonal signaling
Esr2	GTCAGGCACATCAGTAACAAGG	TGAGCATTCAGCATCTCCA	hormonal signaling
Ezh2	CAAAGGATACAGACAGTGACAGAG	CCGAGAATTTGCTTCAGAGGAG	hormonal signaling
Foxl2	GCAAGGGAGGCGGGACAACAC	GAACGGGAACTTGGCTATGATGT	hormonal signaling
Foxo3	CAAACGGCTCACTTTGTCCC	TCATTCTGAACGCGCATGAA	hormonal signaling
Foxo4	CAACTTTGAGCCAGATCCCTGAG	CTAAAGTAGGGTCCCAAACTCCTG	hormonal signaling
Fshr	GGCTCGGGATCTGGATGTCA	TCTCCAGGTCCCCAAATCCA	hormonal signaling
Fst	GTCTGTGCCAGTGACAATGCC	TTCCTCCGTTTCTTCCGAGATG	hormonal signaling
Inha	GGCGTCTGCCTCGAAGACAT	GTTGGGATGGCCGGAATACA	hormonal signaling
Inhba	CAGGAGGGCCGAAATGAATG	CGGATGGTGACTTTGGTCCTG	hormonal signaling
Inhbb	GAGCGCGTCTCCGAGATCAT	CTCCTGCTGCCCTTCTCCAG	hormonal signaling
Kiss1	CGGACCCCAGGAACTCGTTA	GGCATGGCGACGACCTAC	hormonal signaling
Ldlr	AGGTGTGAAGATATTGACGAGTG	TGAAGAGCAGATAGCCTATGGA	hormonal signaling
Lhchr	TGCCTTTGACAACCTCCTCA	TCGAAACATCTGGGAGGGTC	hormonal signaling
Ncor1	GAGCCTCTGGATATGGTGTTCT	CATTTCCCAGTCTACACTTCACGAA	hormonal signaling
Scarb1	ATTTGGCCTGTTTGTTGGGA	ATAATCGATCTTGCTGAGTCCGT	hormonal signaling
Star	TCTCTGCTTGGTTCTCAACTGG	AAACACCTTGCCCACATCTG	hormonal signaling
Atm	ATTGGGATGCTGTTTTCAGG	TAGCCTGGGTGCTCTTTTGT	DNA repair
Brca1	CCCAAAGAAGTAATGACCGTG	GCTAACTATCCACTTTCCTCCTG	DNA repair
Ddb1	GACATCTCAGCTCGTATCCT	GTCACTCAGTAAACCTGTCTC	DNA repair
Fancg	GGGTGATTTGCACGTCCTCTG	GGGTGATTTGCACGTCCTCTG	DNA repair
Dmc1	GGCCCAGATGTTGTCACGACTC	TCAGTTCTCCTCTTCCCTTGCG	meiosis
Mis18	CGTTGAAAGTTACACCTTAGGGTC	GGCTGTCAGGACTTCTTCCA	meiosis
Mus81	GCATCATAAGCAGCCAGGA	CTGTCAAGAACAAGCCCAGT	meiosis
Rad51	ATTGGTTCCAATGGGTTTCA	GGCATGTAACAGCCAACGTA	DNA repair/ meiosis
Rnf168	AGATTGTTGGCTGAGGAGGA	TCTCATAGTTGCTGTTGATACTGG	meiosis
Sycp3	GGAAGATGTGGAAAAGAATAATGATA	AATAACATGGATTGAAGAGACTTTCG	meiosis
Xrcc5	TGTGGACGCAAGAACGCTAA	CTTCCGTAGCGGTACCCTTG	DNA repair
Parp1	CTCCCGGACCTGCTTCACAT	GAAGATCCAGTACCTGCTCTCC	DNA repair
H2afx	GGCCATCCGCAACGACG	TTCTTGCCGACCGCCG	DNA repair

Additional file1: Table S2. Primers used in this work (*continued*)

gene	forward	reverse	function
Hdac1	AGCCATCTTTAAGCCAGTCATGTC	GAAACTCTTCACGAACTCCACAC	histone deacetylase
Hdac11	GAGAAGCTGCACCCCTTTGA	GTTGAGATAGCGCCTCGTGT	histone deacetylase
Hdac2	TGTCAATTTTCCCATGAGAGATGGT	CGCGCTAGGCTGGTACATC	histone deacetylase
Hdac3	CCCCACCAATATGCAGGGTT	AGAGATGCGCCTGTGTAACG	histone deacetylase
Hdac5	GCATTCTACAACGATCCCTCTG	CACCACTGTCCTGAAGGCTG	histone deacetylase
Hdac6	CACCGCATTCAGAGGGTTCT	CCTTAAGGTGGGGCCAGAAG	histone deacetylase
Hdac8	ATGGAGATGGTGTAGAAGATGCCT	TGACACTGTAGTACCGTCCTTTCC	histone deacetylase
Sin3a	GTGTGGACACGTTGGTCAGA	TTGACACTTCCGGATACGCC	histone deacetylase
Sirt2	TAGACGAGCTGACCCTCGAA	CCAGGTTTGCATAGAGGCCA	histone deacetylase
Sirt3	GCTCAGACTGTGGGGTCCG	TCTCACTGCTTCCTCCACCT	histone deacetylase
Sirt7	ACTTGGTTGTCTACACGGGC	GCACTCACAGGCCTTCCTTT	histone deacetylase
Kat2b	GAGTACCTCTTCACCTGCGT	TGTTCACACCCTGTTCAATACTG	histone acetyltransferase
Kat5	GAGAGGTGAAACGGAAGGTGGA	ATCTGAGCTGTCCTGAGAATCCT	histone acetyltransferase
Kmt2a	GCACTTTGAACATCCTCAACC	CACAGTCTTCCTTAAAGTCCACTC	methyl transferase
Kmt2c	ATGCCTCCTCATTGCCTCAG	AAGAATTGCTCCATCTGTGACCA	methyl transferase
Kmt2d	CATCCCTGTCTTCCCAGATACCA	CACCTCACTTCCCTTGCCCT	methyl transferase
Kmt2e	CATCAAGGGTTAAGGGTTCAGCAC	GCTTCTTCGTAACGATCCATCCA	methyl transferase
Wdr5	CTGAAGATGTCGGCTCAGGG	GCACAGAACAGACCAGGAACT	methyl transferase
Sod1	GGACAATACACAAGGCTGTACCA	CAGTCACATTGCCCAGGTCTC	oxidative stress
Gpx1	TCTCTGAGGCACCACGATCC	TCTTGCCATTCTCCTGGTGTC	oxidative stress
Rpl37a	TGGGGCCTGGACCTACAA	GCAGGGCTTCTACTGGTCTT	housekeeping
Hormad1-met	TTTTGGCGGGAATAGTGGT	AAGCCGAAAATAAACATAATTTAC	germ cell marker
Dazl_met	GAGGTTTTACCACCCGAACT	CTCCAGGCCTCACCCTG	germ cell marker
Stk31_met	CCCAGGGTGTTCTTGTCCAC	CGGGCAGCTCACCGAGAAG	kinase
Ddx4_met	GCCGACGCGGCTTAAACG	CGGTCGCCTGATGCTATTTGT	germ cell marker
Rplp0_met	ACTGGTCTAGGACCCGAGAAG	TCAATGGTGCCTCTGGAGATT	housekeeping
*L1gf-met**	CTCCTTGGCTCCGGGACT	CAGGAAGGTGGCCGGTTGT	retroelement
*L1t_met**	CAGCGGTCGCCATCTTG	CACCCTCTCACCTGTTCAGACTAA	retroelement

* Barau J, Teissandier A, Zamudio N, Roy S, Nalesso V, Herault Y, et al. The DNA methyltransferase DNMT3C protects male germ cells from transposon activity. Science. 2016;354:909–12.
